# Integrated multi-omics strategies for identifying novel therapies in psoriasis

**DOI:** 10.1093/bioinformatics/btag347

**Published:** 2026-05-28

**Authors:** Shucheng Si, Xiaoxiao Wang, Siyan Zhan

**Affiliations:** Research Center of Clinical Epidemiology, Peking University Third Hospital, Beijing, 100191, China; Key Laboratory of Epidemiology of Major Diseases (Peking University), Ministry of Education, Beijing, 100191, China; Research Center of Clinical Epidemiology, Peking University Third Hospital, Beijing, 100191, China; Key Laboratory of Epidemiology of Major Diseases (Peking University), Ministry of Education, Beijing, 100191, China; Research Center of Clinical Epidemiology, Peking University Third Hospital, Beijing, 100191, China; Key Laboratory of Epidemiology of Major Diseases (Peking University), Ministry of Education, Beijing, 100191, China; Department of Epidemiology and Biostatistics, School of Public Health, Peking University, Beijing, 100191, China; Institute for Artificial Intelligence, Peking University, Beijing, 100191, China

## Abstract

**Motivation:**

Psoriasis is a chronic, immune-mediated disorder with an unmet need for effective treatments. To systematically prioritize therapeutic targets, we integrated proteome-wide Mendelian randomization (MR) with expression validation in blood/skin, genetic susceptibility analysis, differential gene expression (DGE) from bulk and single-cell RNA sequencing (scRNA-seq), colocalization, pathway enrichment, and protein-protein interaction analyses.

**Results:**

Proteome-wide MR identified 29 candidate protein targets (Bonferroni-corrected), all replicated in independent datasets. Fifteen targets showed significant expression associations in blood or skin. Eleven proteins—UBLCP1, IL23A, ASF1A, RARRES2, ICAM1, PRSS53, ICAM5, GCA, IL2RA, DBI, and NFKB1—exhibited consistent directional effects with their genes. Genetic susceptibility analysis confirmed 20 target-specific polygenic scores for psoriasis and five for psoriatic arthritis. DGE analysis identified 13 targets in bulk and 13 in scRNA-seq—primarily in keratinocytes and immune cells—with IL2RA, COMP, and A2ML1 dysregulated across both. Colocalization analysis implicated shared causal variants for psoriasis in ASF1A, CD8A, CTF1, IL7R, MMP12, RARRES2, XCL2, DBI, IL23A, IL2RA, SGSH, and TIMD4. Enrichment analyses highlighted involvement in cytotoxicity, immune regulation, and JAK-STAT signaling. Eighteen targets interacted with approved anti-psoriasis drugs. Notably, drugs targeting IL2RA, IL7R, CTF1, ICAM1, MMP12, NFKB1, CD8A, DDX58, IL12A, SGSH, and FAP are approved or in trials for other diseases, suggesting repurposing potential. Our integrative multi-omics approach prioritized 29 high-confidence targets, including 13 novel candidates (RARRES2, ASF1A, CTF1, DBI, B3GNT2, CD8A, TIMD4, CRTAM, SGSH, XCL2, DAPK2, A2ML1, and FAP). Several high-priority targets—such as IL2RA, IL23, MMP12, RARRES2, IL7R, and ICAM1—were supported across analytical layers. These findings provide a robust foundation for psoriasis drug development.

**Availability and implementation:**

The code used for the analyses in this manuscript has been archived in Zenodo at [DOI: 10.5281/zenodo.19692128].

## 1. Introduction

Psoriasis, a chronic and relapsing immune-mediated disease, affects approximately 100 million people worldwide, causing substantial physical and emotional distress (WHO 2016). Up to 30% of individuals with psoriasis develop psoriatic arthritis (PsA), resulting in painful joint deformities and functional disability (FitzGerald *et al*. 2021). Furthermore, psoriasis increases the risk of comorbidities, including cardiovascular diseases, metabolic syndrome, and malignancies (Takeshita *et al*. 2017, Wu *et al*. 2022, Zhang *et al*. 2022, Li *et al*. 2024). The profound impact of psoriasis on quality of life, along with its significant financial burden, underscores the urgent need for effective therapeutic strategies (Langley *et al*. 2005). Current standard treatments mainly include immunosuppressants and broad-spectrum anti-inflammatory agents, which generally provide symptomatic relief, while a definitive cure for psoriasis is still lacking. Therefore, identifying new therapeutic targets is essential to achieve better symptom control, reduce relapses, and improve patients’ quality of life.

The development of high-throughput omics technologies has opened unprecedented opportunities for therapeutic target discovery, and prioritizing genetically validated targets enhances the likelihood of clinical success (Nelson *et al*. 2015, Ochoa *et al*. 2022). Given the substantial genetic contribution to psoriasis [heritability about 66% (Grjibovski *et al*. 2007)], genetic insights offer a critical foundation for elucidating the multi-omics landscape and identifying potential disease-modifying agents. Previous studies have identified novel susceptibility alleles and potential therapeutic targets by integrating genome-wide association studies (GWAS) with single-omic data such as transcriptomics and proteomics (Liu *et al*. 2024a, 2024b, Dand *et al*. 2025). However, proteogenomic analyses of psoriasis revealed discordant patterns between mRNA and protein abundance (Swindell *et al*. 2015), highlighting the importance of identifying high-confidence differentially expressed genes (DEGs) through transcriptome-proteome integration. Although multi-omics approaches have revealed promising therapeutic targets for psoriatic arthritis (Cai *et al*. 2025), a systematic investigation focused specifically on psoriasis remains absent. Similarly, proteome-wide association studies (PWAS) using genetic methodologies have been applied to explore potential targets for inflammatory skin disorders, including psoriasis, but have not yielded high-priority targets specific to psoriasis (Li *et al*. 2025). Furthermore, several validated targets (e.g. IL-23 and IL-17) have progressed to clinical development; however, some potential adverse effects, particularly increased infection risks, (Azimi *et al*. 2025, Balogh *et al*. 2020) have also been reported. These limitations highlight both the urgent need for novel target discovery and the imperative for systematic, comprehensive evaluation of candidate targets.

Despite some research dedicated to identifying therapeutic targets for psoriasis, current investigations lag behind comparable studies of other common diseases in the population-based biobanking era (Zhou *et al*. 2022). Specifically, critical gaps persist in: (i) comprehensive multi-omics data mining, (ii) establishing robust population- and individual-level associations, and (iii) multi-level evidence validation of potential targets. We hypothesized that ideal therapeutic targets for psoriasis should demonstrate robust validation across multiple biological levels. A systematic pipeline can provide crucial multi-omic insights into psoriasis, facilitating target identification. To provide credible evidence, we integrated the top three largest proteome-wide GWAS datasets to date (covering 2500+ proteins), blood- and skin-specific gene expression profiles, a large-scale cohort, and single-cell RNA sequencing resources. Using a multidimensional analytical framework incorporating Mendelian randomization (MR, utilizing quantitative trait loci [QTLs] as instrumental variables to mitigate confounding and reverse causality), colocalization analysis, genetic susceptibility analysis, gene-set enrichment, and protein-protein interaction (PPI) network analysis, we systematically constructed a multi-omics landscape of psoriasis to prioritize potential therapeutic targets.

## 2. Materials and methods

### 2.1 Study design

**Figure 1:**
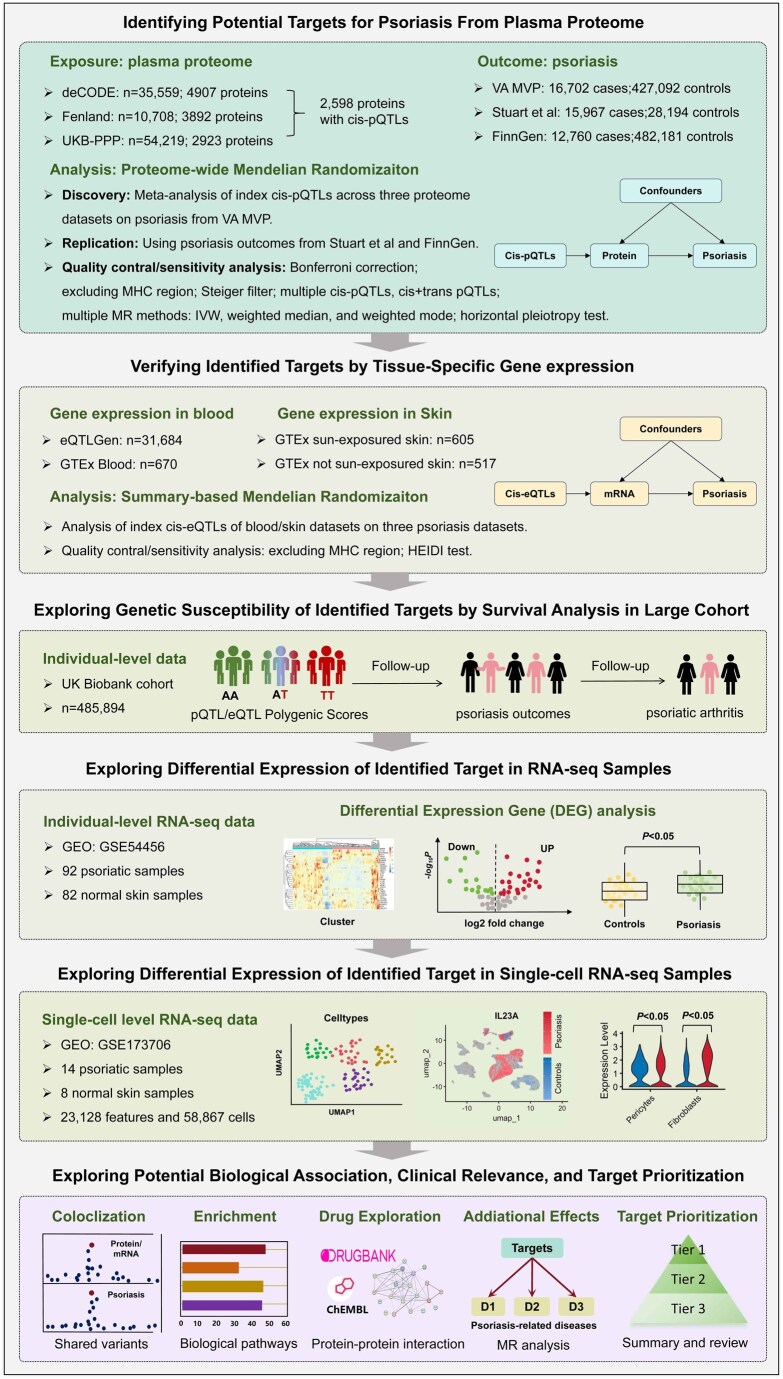
Overview of the study design.

This study used a multi-stage pipeline to identify therapeutic targets for psoriasis (Fig. 1). First, we applied multiple PWAS analyses to identify potential protein targets associated with psoriasis. Then, we validated the coding genes of identified protein targets using tissue-specific gene expression data and constructed target-specific polygenic scores (PGS) to assess their associations with psoriasis and its potential progression to PsA through survival analysis in a large cohort. Furthermore, we examined the differential expression of the identified targets in both bulk RNA-seq and single-cell RNA-seq samples. Subsequently, we conducted colocalization analysis, enrichment analysis, PPI analysis, and additional MR analysis with psoriasis-related diseases to confirm the biological and clinical relevance of these targets. Finally, we prioritized these potential psoriasis targets based on the combined evidence scores (each analysis counting as one line of evidence) from the pipeline and literature review to support future drug development prioritization.

The pipeline followed six steps: (i) Identifying potential protein targets for psoriasis through proteome-wide association analyses by two-sample Mendelian randomization, aligning with replication and sensitivity analysis. (ii) Verifying the coding genes of identified protein targets by tissue-specific gene expression analysis in both blood and skin tissues. (iii) Leveraging individual-level survival analysis to assess genetic susceptibility to psoriasis outcomes using target-specific pQTL/eQTL-derived polygenic scores in the UK Biobank cohort. (iv) Exploring the differential gene expression of identified targets in skin RNA-sequence samples. (v) Exploring differential gene expression of identified targets in skin single-cell RNA-sequence samples. (vi) Exploring potential biological associations and target prioritization by colocalization analysis, enrichment analysis, protein-protein interaction analysis, MR analysis for psoriasis-related diseases, and literature review.

### 2.2 Data sources

#### 2.2.1 Psoriasis genome-wide association data

We accessed genome-wide association data for psoriasis from three large GWAS datasets: (i) A GWAS including 17 702 psoriasis cases and 427 092 controls of European ancestry from the Veterans Affairs Million Veteran Program (VA MVP) (Verma *et al*. 2024). (ii) A GWAS comprising 15 967 psoriasis cases and 28 194 controls of European ancestry from Stuart *et al.* (2022). (iii) A cohort of 12 760 psoriasis cases and 482 181 controls from FinnGen Release 12 (Kurki *et al*. 2023).

#### 2.2.2 Proteome genome-wide association data

To maximize proteome coverage, we considered plasma proteins measured in the three largest proteomic cohorts: (i) 4907 plasma proteins measured in 35 559 Icelanders using the SomaScan platform from the deCODE consortium (Ferkingstad *et al*. 2021). (ii) 3892 plasma proteins from 10 708 European-descent participants in the Fenland study, also using the SomaScan platform (Pietzner *et al*. 2021). (iii) 2923 plasma proteins from 54 219 participants in the UK Biobank Pharma Proteomics Project (UKB-PPP) using the Olink platform (Sun 2023). pQTL summary statistics were obtained from a previously published GWAS of UKB-PPP and deCODE (Eldjarn *et al*. 2023), and from an independent GWAS of the Fenland study (Pietzner *et al*. 2021). These datasets were integrated based on UniProt IDs and gene symbols. Proteins with available pQTL results that could be harmonized with the outcome data were retained, yielding 2598 unique proteins for PWAS. The pQTL data represent the effect of each additional allele on a 1-SD change in protein levels. Table 1, available as supplementary data at *Bioinformatics* online provides the detailed information on these pQTLs.

**Table 1:** Summary of the findings for candidate targets.^a^^,^^b^

Targets	Overall analysis pipeline and findings											Previous evidence with psoriasis			Potential drug or clinical trial phase
	PWAS (principal analysis)	MR sensitivity analysis	SMR by Blood mRNA	SMR by Skin mRNA	Genetic susceptibility for psoriasis	Genetic susceptibility for psoriatic arthritis	Bulk RNA-seq in skin tissue	Single-cell RNA-seq in skin tissue	Coloc	PPI with/or psoriasis drug targets	Evidence Tier 1–3 (scores)	GWAS/genotype studies	Observational studies	MR studies	
IL2RA	**+**	**+/**−	**+**		**+/**−		**↑**	**↑**	**√**	**√**	Tier 1(7)			√	Approved
IL23	−	−	−		−	−	**↑**		**√**	**√**	Tier 1(6)	√	√		Approved^c^
MMP12	**+**	**+**			**+**	**+**	**↑**		**√**	**√**	Tier 1(6)		√	√	Phase 1
RARRES2	−	−	−	−	−			**↑**	**√**	**√**	Tier 1(6)				
IL7R	**+**	**+**			**+**	−	**↑**	**↓**	**√**	**√**	Tier 1(5)		√		Phase 2
ICAM1	−	**+/**−	−		**+/**−			**↓**		**√**	Tier 1(5)		√	√	Approved
ASF1A	−	−	−						**√**	**√**	Tier 2(4)				
COMP	**+**	**+**					**↑**	**↑**		**√**	Tier 2(4)		√		
CTF1	−	−			−				**√**	**√**	Tier 2(4)				Phase 1
DBI	**+**	**+**	**+**					**↑**	**√**		Tier 2(4)				
ICAM5	**+**	**+**	−	**+**	**+/**−					**√**	Tier 2(4)			√	
PRSS53	**+**	**+**	−	**+**	**+/**−		**↑**				Tier 2(4)	√			
TNFAIP3	−	−	**+**		**+/**−			**↓**		**√**	Tier 2(4)	√	√	√	
DDX58	**+**	**+**			**+/**−		**↑**			**√**	Tier 2(4)	√	√		Phase 2
IL12	−	−	**+**		−	−				**√**	Tier 2(4)		√		Approved/Phase 3^c^
IL12B	−	−			−	−	**↑**			**√**	Tier 2(4)	√	√	√	Approved/Phase 3^c^
UBLCP1	−	−		−	−			**↓**			Tier 2(4)			√	
B3GNT2	−	−			−	−		**↓**			Tier 2(4)				
NFKB1	**+**	**+**	**+**		**+**			**↓**		**√**	Tier 2(4)	√	√	√	Approved
CD8A	−	**+/**−				**+**	**↑**		**√**	**√**	Tier 3(3)				Phase 2
TIMD4	−	−	**+**				**↑**		**√**	**√**	Tier 3(3)				
CRTAM	−	−			−					**√**	Tier 3(3)				
GCA	**+**	**+**	**+**		**+**			**↓**			Tier 3(3)	√			
SGSH	−	−	**+**	**+**	**+/**−				**√**		Tier 3(3)				Phase 2/3
XCL2	−	−			−	**+**	**↑**		**√**		Tier 3(3)				
DAPK2	**+**	**+**								**√**	Tier 3(2)				
LBP	−	−					**↑**			**√**	Tier 3(2)		√		
A2ML1	−	−					**↑**	**↑**			Tier 3(1)				
FAP	−	−						**↑**			Tier 3(1)				Phase 3

a Total evidence score (ranged 0–9) was counted by summing the significant results of MR sensitivity analysis, blood/skin mRNA SMR analysis, survival analysis for genetic susceptibility of QTL PGS, bulk RNA-seq, single-cell RNA-seq, colocalization, and PPI analysis, where the direction was consistent with PWAS (count = 1 if the direction was consistent or not direction-dependent, count = 0 if opposite). + and − indicated the associated direction of the target with psoriasis, where + represented a positive association, − represented a negative association, and +/− represented that both associations were observed. ↑ and ↓ indicated the up-regulated or down-regulated gene expression (mRNA) in psoriasis cases compared to healthy controls.

b √ indicated passed the analysis.

c The target was the current psoriasis drug target.

#### 2.2.3 Transcriptome genome-wide association data

We used transcriptomic data (eQTLs) from two major GWAS sources: (i) blood-derived expression data from 31 684 European-ancestry individuals from the eQTLGen Consortium (Võsa *et al*. 2021). (ii) tissue-specific eQTLs from GTEx v8, including whole blood (*n *= 670), sun-exposed skin (*n *= 517), and non-sun-exposed skin (*n *= 605) samples from European-ancestry individuals (The GTEx Consortium 2020). Only eQTLs of significant coding genes in the PWAS were utilized. The eQTL data are presented as the effect of each additional allele on a 1-SD change in gene expression, with corresponding associations detailed in Table 2, available as supplementary data at *Bioinformatics* online.

#### 2.2.4 UK Biobank cohort

The UK Biobank cohort includes over 500 000 participants aged 37–73 years, recruited between 2006 and 2010 across 22 assessment centers in the UK (Sudlow *et al*. 2015). At recruitment, participants completed a touchscreen questionnaire covering sociodemographic data, lifestyle factors, medical history, and physical measurements, and provided blood samples for genome-wide genotyping. Psoriasis was defined according to the first occurrence record with field ID 131742 (ICD-10 codes L40). Further details can be found online at www.ukbiobank.ac.uk. We further defined incident psoriatic arthritis among individuals with psoriasis as a potential progression of the disease, corresponding to ICD-10 codes L40.5, M07.0, M07.1, M07.2, M07.3, and M09.0. Ethical approval was obtained from the North West Multi-center Research Ethics Committee, and all participants provided informed consent.

#### 2.2.5 Bulk RNA-sequence and single-cell RNA-sequence samples

We obtained skin bulk RNA-seq and single-cell RNA-seq samples from the GEO database with accession IDs GSE54456 (Li *et al*. 2014, Tsoi *et al*. 2015) and GSE173706 (Merleev *et al*. 2022, van Straalen 2024). The bulk RNA-seq profiled the transcriptomes of 92 lesional psoriatic samples and 82 normal skin samples. The single-cell RNA-seq samples analyzed transcription changes in normal skin from healthy donors (*n* = 8) and psoriatic skin from psoriasis patients (*n* = 14). A total of 23 128 gene features and 58 867 cells were assessed from the 22 samples.

### 2.3. Statistical analysis

#### 2.3.1 Proteome-wide association analysis

We aimed to identify potential protein targets for psoriasis by conducting two-sample MR with three large plasma proteomics datasets. The MR method should satisfy three assumptions: (i) the IV is associated with the exposure, (ii) the IV affects the outcome only through the exposure, and (iii) the IV is not associated with the confounders.

In the discovery phase, we used the index *cis*-pQTLs as instrumental variable (IV) for each protein, followed by the previous study (Yuan *et al*. 2023), with the outcome based on data from VA MVP, which provided the largest psoriasis GWAS sample size without sample overlap with exposure data. For each protein, odds ratios (ORs) and 95% confidence intervals (CIs) were calculated using the Wald ratio method (Pierce and Burgess 2013). MR estimators represent the effect of per-SD increases in genetically predicted protein levels and their association with psoriasis risk. To adjust for multiple comparisons, the associations that passed the Bonferroni correction level (*P *< 1.89 × 10^−5^) were considered significant. For proteins available across multiple datasets, a fixed-effect meta-analysis model was applied to derive 'combined’ effect estimates. In the replication phase, two independent psoriasis datasets (Stuart *et al.* and FinnGen) were used to validate the findings. Proteins that met multiple testing criteria and replicated in at least one external dataset were considered potential targets.

The strength of the IVs was assessed using *F*-statistics (all > 10), indicating no evidence of weak instrument bias (Bowden *et al*. 2016). To mitigate reverse causality and pleiotropy, we applied the Steiger filter to exclude pQTLs, which explained more variance for psoriasis than for the corresponding proteins. Additionally, proteins encoded by genes located within the major histocompatibility complex (MHC) region (chromosome 6, GRCh37 position 25–33 Mb) were excluded from the analysis. Sensitivity analyses were performed using both cis- and trans-pQTLs. A further sensitivity analysis was conducted using multiple cis-pQTLs identified by clumping the GWAS summary statistics at *r*^2^ < 0.1 and *P *< 5 × 10^−6^, followed by application of the inverse-variance weighted, weighted median, and weighted mode methods. Horizontal pleiotropy was evaluated using the intercept of the MR-Egger regression.

#### 2.3.2 Tissue-specific gene expression association analysis

To validate the coding genes of identified protein targets, we performed summary-based Mendelian Randomization (SMR) to examine the association between gene expression (mRNA) and psoriasis risk in blood samples (eQTLGen and GTEx blood tissue) and skin samples (GTEx sun-exposed and no-sun-exposed skin tissues). SMR utilized the top *cis*-eQTL for each gene as an instrumental variable, applying a default significance threshold of *P *< 5 × 10^−8^ (Zhu *et al*. 2016). Linkage was assessed using the HEIDI test, where a *P-*value < .01 indicated a linkage scenario rather than gene expression influencing the outcome. The main results are reported as ORs per 1-SD increase in gene expression. Genes showing significant associations in at least one dataset were considered successfully validated. We further assessed whether the same variants identified as index plasma pQTLs showed concordant effect directions with those in GTEx skin eQTLs for their corresponding genes.

#### 2.3.3 Survival analysis of genetic susceptibility using target-specific polygenic scores

For the analysis of individual genetic susceptibility, we extracted multiple cis-pQTLs and cis-eQTLs for each identified target in UK Biobank participants, using thresholds of *P *< 5 × 10^−8^, *r*^2^ < 0.01, and a cis-window of ±1000 kb. Based on these variants, we constructed target-specific weighted PGSs, with QTL effect sizes (β) used as weights. These target-specific PGSs reflect genetically predicted protein abundance and gene expression levels. Follow-up for psoriasis was defined from birth until psoriasis diagnosis or censoring (December 2022), whichever came first. For PsA, follow-up began at psoriasis onset and continued until PsA diagnosis or censoring. Cox proportional hazards models were used to estimate hazard ratios (HRs) and 95% CIs, adjusted for sex and the top five genetic principal components (PCs).

#### 2.3.4 Differential expression gene analysis in bulk RNA-sequence samples

We aimed to identify DEGs between psoriatic and normal skin samples. Gene expression counts were normalized using the calcNormFactors function from the edgeR package, and transformed to log2 counts using the voom function from the limma package. DEGs were identified by fitting a linear model using the lmFit function and applying the empirical Bayes method with the eBayes function, both from the limma package. Genes with an absolute log_2_ fold change (|log2FC|) ≥1 and FDR-adjusted *P-*value < .05 were viewed as significant.

#### 2.3.5 Single cell-type expression analysis

Single-cell RNA-seq data were processed using the Seurat package following the standard Seurat pipeline for quality control, normalization, and transformation. Low-quality cells were excluded based on the following criteria: <500 or >6000 detected genes, >10% mitochondrial gene expression, or total UMI counts <1000 or >30 000 per cell. Data were normalized and scaled using the NormalizeData, FindVariableFeatures, and ScaleData functions. Principal component analysis (PCA) was performed, and the top 30 PCs were used for cell clustering and visualization via uniform manifold approximation and projection (UMAP) plots. Cell types were annotated based on known marker genes and reference annotations from a previous study (Yuan *et al*. 2024). DEG analysis was conducted using the Wilcoxon rank-sum test (i) to compare the gene expression levels of candidate targets in a given cell type versus all other cell types, (ii) to compare the expression levels of candidate targets in enriched cell types between psoriatic and normal skin samples. The genes with |log2FC| ≥0.25 and FDR-adjusted *P-*value < .05 were viewed as significant.

#### 2.3.6 Colocalization analysis

To evaluate whether the target proteins and coding genes share a common causal variant with psoriasis, we conducted a Bayesian colocalization analysis. This method estimates posterior probabilities for five hypotheses (PPH): (H_0_) no association with either trait; (H_1_) association with trait 1 (core proteins/genes) only; (H_2_) association with trait 2 (psoriasis) only; (H_3_) distinct causal variants associated with each trait; and (H_4_) a shared causal variant associated with both traits (Foley *et al*. 2021). The ‘coloc.abf’ algorithm was used with default settings. Strong colocalization was defined as PPH_4_ > 0.8, and moderate colocalization as PPH_4_ > 0.5.

#### 2.3.7 Enrichment analysis

Gene Ontology (GO) and Kyoto Encyclopedia of Genes and Genomes (KEGG) enrichment analyses were performed to annotate functions of identified protein-coding genes. These analyses assessed the biological relevance of candidate genes across three GO categories: biological processes (BP), cellular components (CC), and molecular functions (MF), and KEGG pathways. Enrichment was considered statistically significant at a *q*-value < 0.05. Significantly enriched GO terms and KEGG pathways were identified to highlight relevant biological processes and mechanisms.

#### 2.3.8 Drug and clinical relevance exploration

We queried the DrugBank (https://go.drugbank.com/) and ChEMBL (https://www.ebi.ac.uk/chembl/) databases to identify the current psoriasis drug targets and their corresponding actions. We then explored potential druggability by analyzing PPIs between our identified targets and existing anti-psoriasis drug targets using the STRING database (https://string-db.org/) (Szklarczyk *et al*. 2019). A PPI between a potential target and a drug target was defined based on a combined score >0.4, which incorporates co-expression, experimentally determined interaction, database-annotated, and automated text-mining associations. To assess the concordance between the directional effects of the identified targets and the modes of action of their interacting approved drug targets in the PPI network, we annotated both the drug target actions and the effect directions of the identified targets by integrating bulk and Single-cell RNA sequencing (scRNA-seq) expression, protein MR, and mRNA SMR results. To investigate potential biological roles, side effects, and off-target implications, we conducted additional two-sample MR analyses linking the targets to 22 psoriasis-related conditions, including PsA, cardiovascular diseases, metabolic disorders, and autoimmune diseases. Finally, we calculated a composite evidence score based on the full analysis pipeline, which prioritized the identified targets. A corresponding literature review was also conducted to evaluate the novelty of our findings.

All analyses were conducted using the R software (4.4.2) and the SMR software.

## 3. Results

### 3.1 Plasma proteome associations with psoriasis

**Figure 2:**
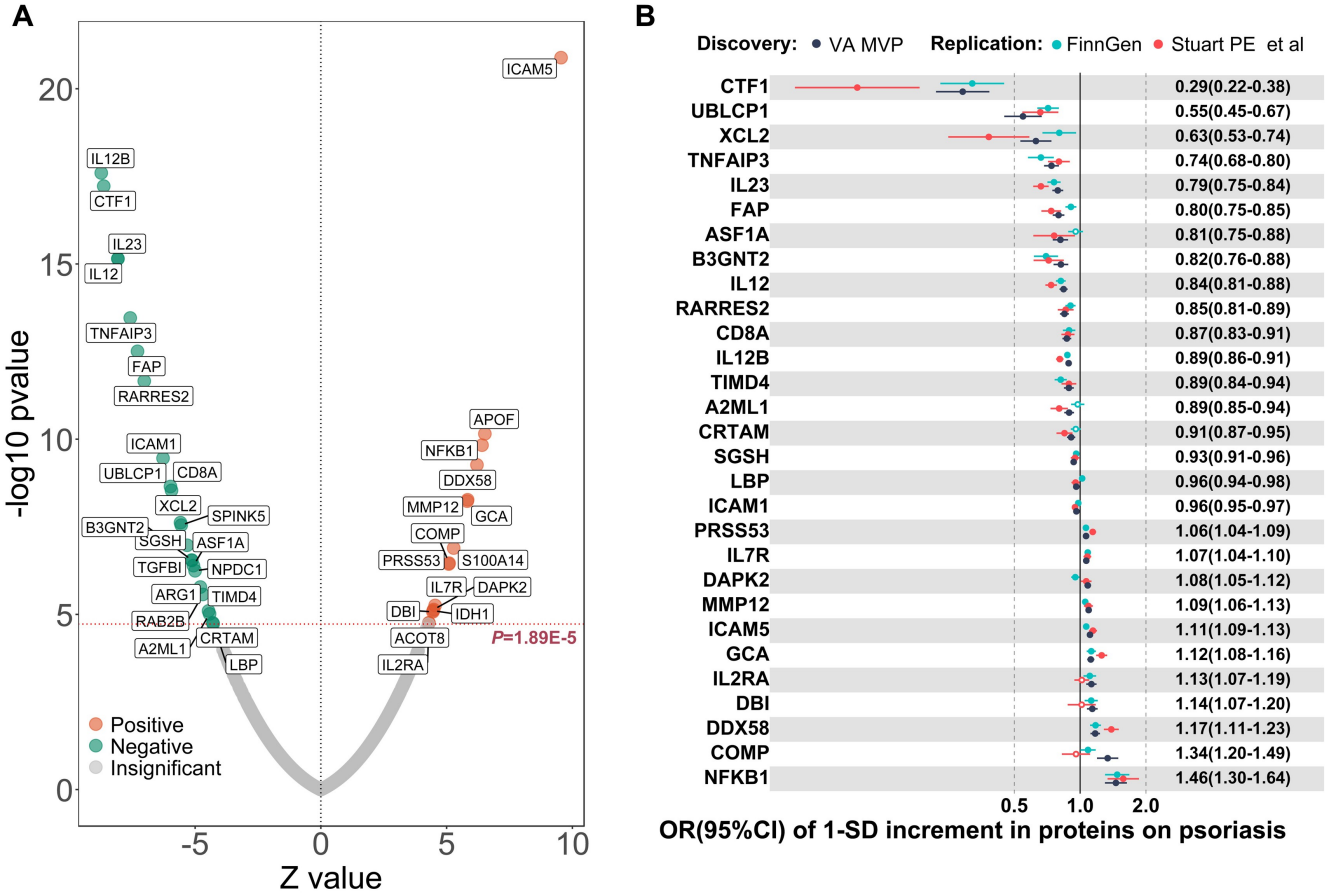
Identified proteins associated with psoriasis in proteome-wide association analysis. (A) Volcano plot of individual proteome-wide association analysis. This plot displays the association of the plasma proteome with psoriasis outcomes. Each point represented a protein. Points above the red line indicate passed the Bonferroni correction. The color of the points represents the direction of associations. (B) Forest plot of identified proteins in A. This forest plot presents odds ratios (ORs) and 95% confidence intervals (CIs) for a 1-SD increase in proteins associated with psoriasis. The OR (95% CI) was the combined effects if multiple protein data that are available. Only 29 proteins that passed the replication analysis are shown.

**Figure 3:**
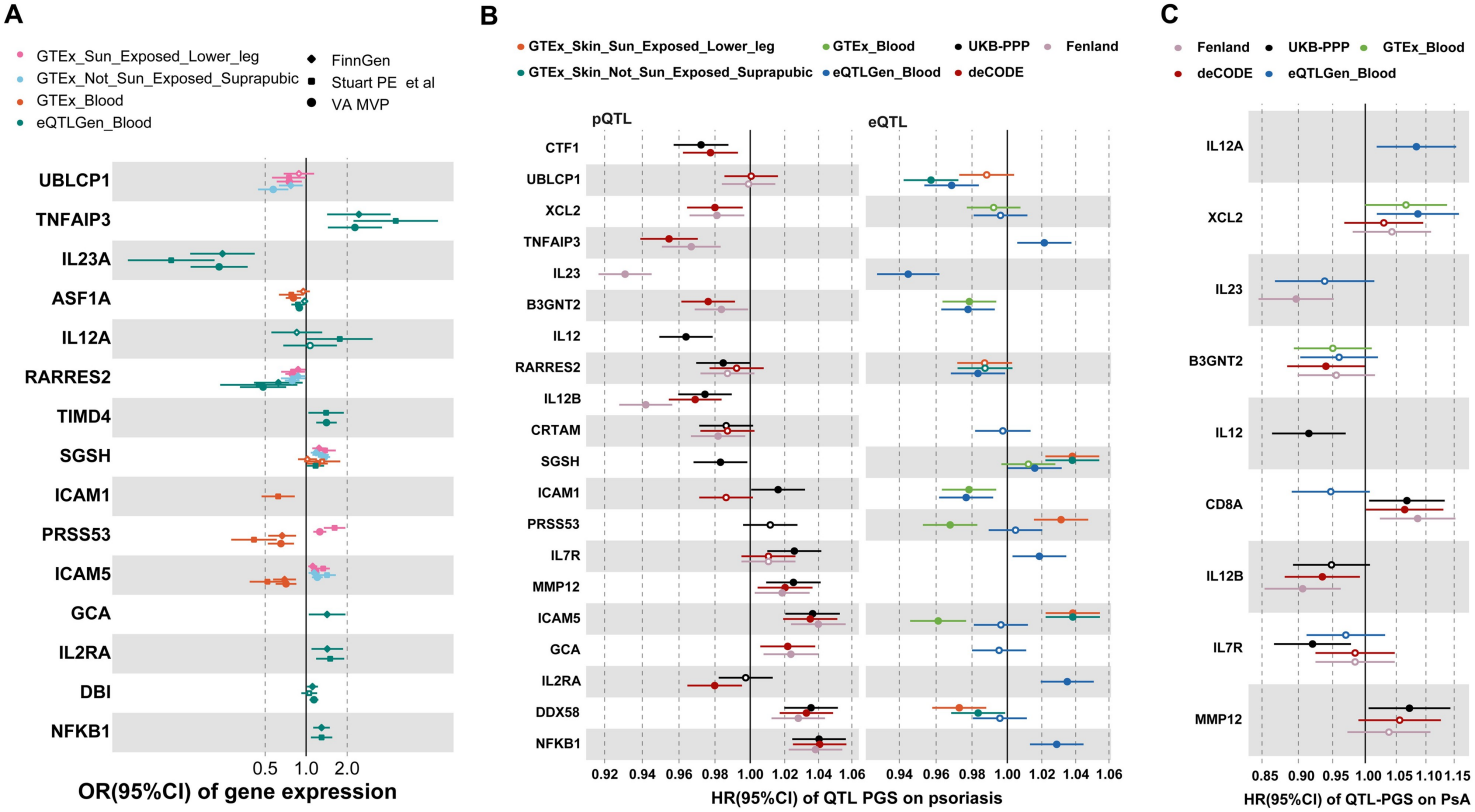
Associations of coding gene expression and QTL PGS with psoriasis. (A) Forest plot of coding gene expression in blood and skin (sun-exposed and unexposed). Only genes significant in any one of the three outcome data are shown. (B) Individual-level survival analysis of target-specific polygenic scores (PGSs) for psoriasis. The analysis focused on PGSs constructed from pQTLs and eQTLs corresponding to the identified targets. Only targets with at least one significant PGS association are shown. (C) Individual-level survival analysis of target-specific PGSs for psoriatic arthritis. Only targets with at least one significant PGS association are shown.

Proteome-wide MR analysis identified 38 of 2647 proteins significantly associated with psoriasis (*P *< 1.89 × 10^−5^) in the discovery dataset [Veterans Affairs Million Veteran Program (VA MVP)] after Bonferroni correction (Fig. 2A). Of these, 29 potential targets were verified (*P *< .05) in replication datasets from either Stuart *et al.* or FinnGen (Fig. 2B, detailed pQTLs and results are in Tables 1 and 2, available as supplementary data at *Bioinformatics* online and Fig. 1, available as supplementary data at *Bioinformatics* online). Specifically, 18 proteins were negatively associated with psoriasis (e.g. CTF1, UBLCP1, XCL2, TNFAIP3, IL23, FAP, ASF1A, B3GNT2, IL12, RARRES2, CD8A, IL12B, TIMD4, A2ML1, CRTAM, SGSH, LBP, and ICAM1), while 11 were positively associated (e.g. PRSS53, IL7R, DAPK2, MMP12, ICAM5, GCA, IL2RA, DBI, DDX58, COMP, and NFKB1). Sensitivity analysis, using multiple MR methods or incorporating both cis- and trans-pQTLs, confirmed the majority of these results (Figs 2 and 3, available as supplementary data at *Bioinformatics* online).

### 3.2 Gene expression and genetic susceptibility associations with psoriasis and psoriatic arthritis

At the transcriptome level, 15 protein-coding genes of the 29 potential targets demonstrated significant associations in either blood or skin tissue (*P *< .05 and *P*_HEIDI_ > .01). Among these, 11 genes (UBLCP1, IL23A, ASF1A, RARRES2, ICAM1, PRSS53, ICAM5, GCA, IL2RA, DBI, and NFKB1) showed consistent direction with their corresponding proteins (Fig. 3A, Table 3, available as supplementary data at *Bioinformatics* online). Notably, for PRSS53 and ICAM5, the association directions differed between blood- and skin-derived expression, with the skin results aligning with the protein direction.

Target-specific PGS survival analysis in the UK Biobank cohort (*n* = 485 894; 16 098 incident psoriasis cases) identified 20 targets whose PGSs were significantly associated with incident psoriasis (*P *< .05), including CTF1, UBLCP1, XCL2, TNFAIP3, IL23, B3GNT2, IL12, RARRES2, IL12B, CRTAM, SGSH, ICAM1, PRSS53, IL7R, MMP12, ICAM5, GCA, IL2RA, DDX58, and NFKB1 (Fig. 3B). For each target, at least one PGS showed a significant association in a direction consistent with that of the corresponding protein target, although seven targets (TNFAIP3, SGSH, ICAM1, PRSS53, ICAM5, IL2RA, and DDX58) showed associations in both directions across pQTL- or eQTL-based PGSs derived from different databases. Eight targets were also significantly associated with incident PsA, including XCL2, IL23, B3GNT2, IL12, CD8A, IL12B, IL7R, and MMP12, of which five (IL23, B3GNT2, IL12, IL12B, and MMP12) were directionally consistent with their corresponding protein targets (Fig. 3C). For IL12, a significant association was also observed for the PGS based on its coding gene IL12A. Comparison of plasma pQTLs with GTEx skin eQTLs showed concordant effect directions between plasma and skin for COMP, FAP, ICAM1, PRSS53, RARRES2, and ICAM5. In contrast, DDX58, NFKB1, and SGSH showed opposite effect directions, consistent with the findings from the genetic susceptibility analysis (Table 4, available as supplementary data at *Bioinformatics* online).

### 3.3 Differential expression genes in bulk RNA-seq and scRNA-seq samples

**Figure 4:**
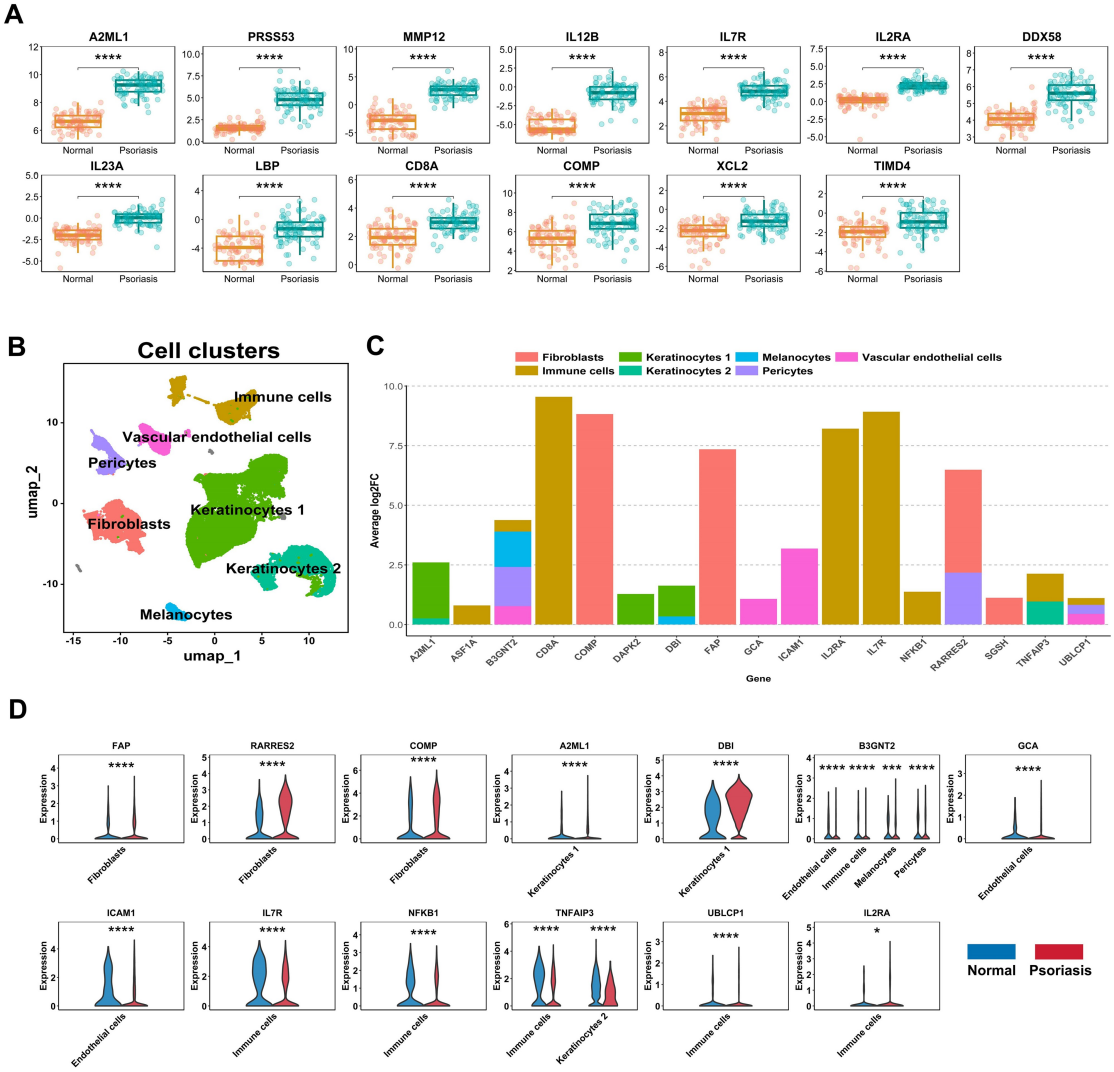
Results of differential gene expression in bulk- and single-cell RNA-seq samples. (A) Box plot of 13 differential expression genes (DEGs) in psoriasis and normal samples. The vertical axis represents scaled gene expression. Genes with |log2FC| ≥1 and FDR-adjusted *P* < .05 were considered significant. (B) UMAP visualization of identified cell types. The cell types were characterized by the expression of marker gene: keratinocytes (KRT14, KRT5, KRT10, KRT1, SBSN, and KLK7); fibroblasts (DCN and LUM); vascular endothelial cells (CLDN5 and VWF); immune cells (KLA-DPB1, CD3D, and CD3E); Melanocytes (MLANA and DCT); pericytes (ACTA2 and TAGLN). (C) Significant cell-specific enrichment for 20 coding genes. Only significant results are shown, which represent that the expression of a specific gene in a specific cell type was different from the expression in other cell types. (D) Violin and scatter plot of 16 differential expression genes between psoriasis and healthy controls. Only significant results are shown, which represent that the expression of a specific gene in a specific cell type was different in psoriasis and healthy controls. |Log2FC|>0.25 and an FDR-adjusted *P* < .05 was considered significant. *P < .05; **P < .01; ***P < .001; ****P < .0001.

**Figure 5:**
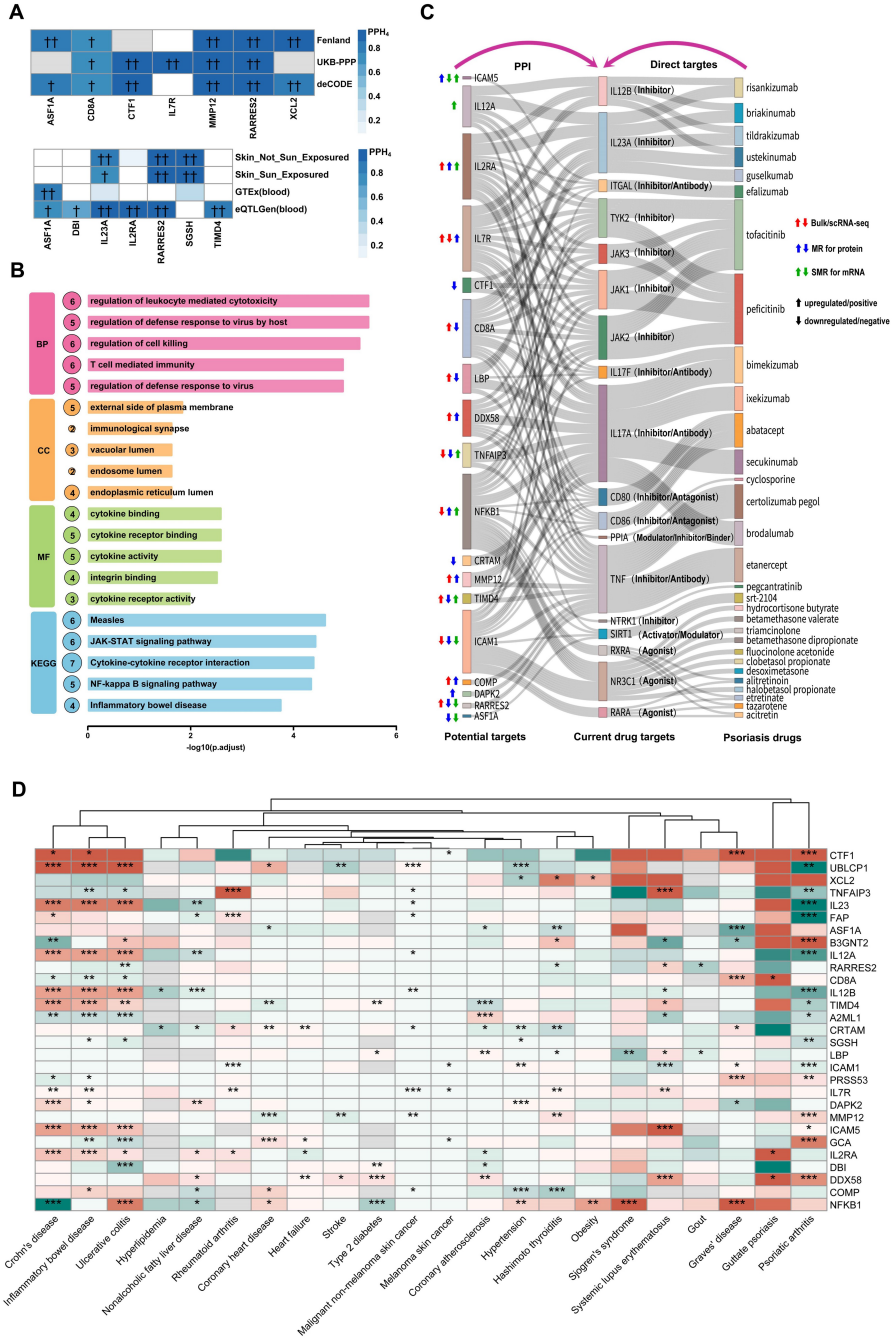
Bioinformatic analysis, drug identification, and clinical relevance for identified targets. (A) Colocalization analysis of protein and gene expression with psoriasis, respectively. The color represents the posterior probabilities (PP) that colocalized for a shared causal variant. Only proteins/genes that passed the colocalization analysis with PPH_4_ > 0.5 in at least one dataset are shown. †† indicates PPH_4_ > 0.8, while † indicates 0.5 < PPH_4_ < 0.8. (B) The top enriched GO terms in enrichment analysis for each category of biological process (BP), cellular component (CC), and molecular function (MF), and the top KEGG pathways. (C) Interaction between identified targets and current targets of anti-psoriasis drugs. This panel illustrates protein-protein interactions (PPI) among potential targets identified in this study. It includes targets directly mapped to approved anti-psoriasis drugs (IL23A and IL12B), as well as other novel targets not directly mapped to these drugs. The links between the targets represent the PPI (combined score > 0.4), and the links between targets and drugs represent the known drug target (from DrugBank). (D) Associations of 29 protein targets with 22 psoriasis-related diseases. The color represented the beta estimators by MR analysis. The positive and negative targets represented the directions with psoriasis. *P < .05; **P < .01; ***P < .001.

In bulk RNA-seq datasets, hierarchical clustering based on the identified potential targets revealed distinct expression patterns between psoriasis and control samples (Fig. 4, available as supplementary data at *Bioinformatics* online). Specifically, 13 genes showed upregulated expression in the skin tissue of psoriasis samples (Fig. 4A), including A2ML1, PRSS53, MMP12, IL12B, IL7R, IL2RA, DDX58, IL23A, LBP, CD8A, COMP, XCL2, and TIMD4. scRNA-seq samples allowed us to identify seven cellular clusters, representing six major cell types: keratinocytes (group 1 and 2), fibroblasts, endothelial cells, immune cells, melanocytes, and pericytes (Fig. 4B). Among the 29 potential targets, 17 genes exhibited cell-type-specific enrichment (Fig. 4C, Fig. 5, available as supplementary data at *Bioinformatics* online); for instance, RARRES2 and COMP were predominantly expressed in fibroblasts, while A2ML1 and DBI were mainly enriched in keratinocytes. DEG analysis identified 13 genes that were significantly differentially expressed between psoriasis and normal samples in at least one cell type, including FAP, RARRES2, COMP, A2ML1, DBI, B3GNT2, GCA, ICAM1, IL7R, NFKB1, TNFAIP3, UBLCP1, and IL2RA (Fig. 4D). Overall, 11 targets presented the same regulated direction with the plasma protein, including IL2RA, MMP12, IL7R, ICAM1, COMP, DBI, PRSS53, TNFAIP3, DDX58, UBLCP1, and B3GNT2 in either bulk- or sc-RNA seq. Detailed results are provided in Table 5, available as supplementary data at *Bioinformatics* online.

### 3.4 Shared causal variants with psoriasis in colocalization

Colocalization analysis further identified shared causal variants between these potential targets and psoriasis (Fig. 5A). For proteins, CTF1 (loci rs12934900 and rs2305880), IL7R (rs11742270), MMP12 (rs201707806, rs17368814, rs2276109), RARRES2 (rs3735167), XCL2 (rs61801329, rs10753774), and ASF1A (rs147886588) demonstrated strong colocalization with psoriasis (PPH_4_>0.8) (Fig. 6, available as supplementary data at *Bioinformatics* online). Regarding gene expression, ASF1A (rs147886588), IL23A (rs2695782, rs2643623), IL2RA (rs61839660), RARRES2 (rs11769348, rs57367026, rs1047207), SGSH (rs9914372), and TIMD4 (rs953569) also showed strong colocalization (Fig. 7, available as supplementary data at *Bioinformatics* online). The CD8A and DBI showed moderate colocalization. Detailed results are available in Table 6, available as supplementary data at *Bioinformatics* online.

### 3.5 Pathway enrichment

By enrichment analysis, we identified 385 GO terms and 35 KEGG pathways (Tables 7 and 8, available as supplementary data at *Bioinformatics* online). Significant enrichment was observed in biological processes related to cytotoxicity, cell killing, immunity, and KEGG pathways such as JAK-STAT and RIG-I-like receptor signaling pathways (Fig. 5B).

### 3.6 Drug and clinical relevance exploration

Extensive PPIs were observed between the identified protein targets (potential targets) and current psoriasis drug targets (Fig. 5C). 18 identified targets (including ICAM5, IL12A, IL2RA, IL7R, CTF1, CD8A, LBP, DDX58, TNFAIP3, NFKB1, CRTAM, MMP12, TIMD4, ICAM1, COMP, DAPK2, RARRES2, and ASF1A) interacted with current anti-psoriasis drug targets. Notably, IL23 (encoded by IL23A and IL12B) is directly targeted by existing anti-psoriasis drugs like risankizumab, briakizumab, tildrakizumab, guselkumab, and ustekinumab. We found that, except for CTF1, and CRTAM, all targets showed effect directions consistent with the expected therapeutic effects of their interacting drug targets, although some targets displayed associations in both directions across different analyses. Notably, the protective associations observed for CD8A, NFKB1, ICAM1, and ASF1A may be related to their interaction with SIRT1, whose approved interacting compound SRT-2104 acts as an activator, whereas the remaining interacting drug targets were linked to inhibitory effects. Detailed results are available in Table 9, available as supplementary data at *Bioinformatics* online.

In the MR analysis of 22 psoriasis-related clinical conditions (Fig. 5D), most targets were also associated with PsA, with consistent directions of association except for CTF1 and B3GNT2. Additionally, CD8A, IL12RA, and DDX58 showed significant associations with guttate psoriasis. These targets were also strongly linked to cardiovascular diseases, metabolic disorders, and certain autoimmune diseases. However, some targets exhibited different effects compared to psoriasis, potentially indicating off-target effects, as observed in inflammatory bowel disease and related conditions.

In summary, this study identified 29 potential protein targets associated with psoriasis and characterized their comprehensive multi-omic profiles. We calculated a comprehensive evidence score for the 29 potential targets, based on the number of significant and consistent findings across our multiple analyses (Table 1). Specifically, 11 targets showed consistent causal associations at both the protein and mRNA levels (IL2RA, IL23, RARRES2, ICAM1, ASF1A, DBI, ICAM5, PRSS53, UBLCP1, NFKB1, and GCA); 12 targets showed evidence of shared causal variants (IL2RA, IL23, MMP12, RARRES2, IL7R, ASF1A, CTF1, DBI, CD8A, TIMD4, SGSH, and XCL2); 20 targets were supported by population-level genetic susceptibility analysis, of which five (IL23, B3GNT2, IL12, IL12B, and MMP12) were also consistently associated with psoriasis progression; 11 targets showed consistent expression patterns in either bulk RNA-seq or scRNA-seq analyses, including three targets supported in both datasets (IL2RA, COMP, and A2ML1); and 18 targets showed interactions with existing anti-psoriasis drug targets.

## 4. Discussion

Overall, we established a three-tier evidence framework to prioritize these targets and identified high-priority proteins. High-priority proteins (score ≥ 5) included IL2RA, IL23, MMP12, RARRES2, IL7R, and ICAM1. Additional high-priority targets (score = 4) confirmed through multiple analyses were ASF1A, COMP, CTF1, DBI, ICAM5, PRSS53, TNFAIP3, DDX58, IL12, IL12B, UBLCP1, B3GNT2, and NFKB1. Secondary-priority targets included CD8A, TIMD4, CRTAM, GCA, SGSH, XCL2, DAPK2, LBP, A2ML1, and FAP. Remarkably, a literature review revealed that only eight targets had previous MR evidence, while 13 targets were reported for the first time in the context of psoriasis, including RARRES2, ASF1A, CTF1, DBI, B3GNT2, CD8A, TIMD4, CRTAM, SGSH, XCL2, DAPK2, A2ML1, and FAP. In terms of drug exploration, drugs targeting IL2RA, MMP12, IL7R, ICAM1, CTF1, DDX58, NFKB1, CD8A, SGSH, and FAP are either approved or in clinical trials for other diseases, highlighting promising opportunities for drug repositioning in psoriasis treatment.

We identified robust associations for several high-priority targets, indicating their promise as future therapeutic options. For instance, IL2RA demonstrated consistent positive associations throughout our pipeline, suggesting its potential for developing inhibitors to control psoriasis. Although the understanding of IL2RA’s precise role in psoriasis pathogenesis and underlying mechanisms remains limited, numerous related drugs have been approved or are in clinical trials for conditions such as organ transplant rejection, chronic lymphocytic leukemia, and solid tumors/cancers. These diverse clinical applications highlight the multifaceted roles of this target, thereby opening up possibilities for psoriasis treatment. IL7R, another cytokine, also showed suggestive evidence of association with psoriasis, supported by its differential gene expression in distinguishing non-lesional from normal and lesional samples (Xie *et al*. 2014). Multiple lines of evidence have shown IL-7's significant role in inflammatory diseases, including atherosclerosis, rheumatoid arthritis, psoriasis, multiple sclerosis, and inflammatory bowel disease, where it mediates inflammation. IL7R and CD8A have suggestive evidence of association with psoriasis, supported by their DEGs in distinguishing non-lesional from normal and lesional samples (Xie *et al*. 2014) and by the established roles of CD8+ T cells (Dong *et al*. 2024, Saw and Song 2025).

RARRES2, previously identified as a potential therapeutic target for atopic dermatitis and acne (Li *et al*. 2025). This gene encodes chemerin, a multifunctional adipokine that regulates immune functions, including inflammation and dendritic cell chemotaxis. Previous studies have reported that Chemerin expression is primarily localized in fibroblasts, mast cells, and endothelial cells within the skin (Wittamer *et al*. 2003, Zabel *et al*. 2005, Yoshimura and Oppenheim 2011). Our scRNA-seq analysis revealed cell-specific enrichment of RARRES2 in fibroblasts and pericytes, with significantly higher expression in fibroblasts from psoriasis compared to healthy samples. Its potential mechanism in psoriasis may involve chemerin-mediated modulation of immune responses and skin barrier function, with its role in recruiting immune cells to inflammatory sites suggesting involvement in early psoriasis development (Albanesi *et al*. 2009). NFKB1 also emerged as a risk factor, with genetic variation correlating with disease severity (Nikamo *et al*. 2015). The transcription factor NF-κB is recognized as a key regulator in the pathogenesis of psoriasis. The activation of the NF-κB signaling pathway induces the transcription of pro-inflammatory cytokines, chemokines, and growth factors, which contribute to both the initiation and persistence of psoriasis. Previous scRNA sequence of circulating immune cells revealed that TNFAIP3 may serve as a potential biomarker for psoriatic arthritis (Garrido *et al*. 2025). TNFAIP3 may act as a negative regulator of IL-17 and NF-κB signaling and is negatively associated with psoriasis (Francis *et al*. 2024). The finding implies that TNFAIP3 may exert synergistic effects with drugs targeting IL17, potentially enhancing psoriasis treatment. MMP12 (Matrix Metalloproteinase 12) was also found to be upregulated in psoriatic skin lesions, consistent with previous findings. As an elastolytic enzyme secreted by inflammatory macrophages, it has been shown to suppress angiogenesis (Mao *et al*. 2024). In addition, DDX58, encoding the RNA sensor RIG-I, was linked to psoriasis susceptibility, with RIG-I activation triggering IL-23 production and psoriasis-like symptoms in models (Zhu *et al*. 2017).

Other potential targets, such as UBLCP1 (ubiquitin-like domain containing CTD phosphatase 1), is a promising gene that may link NF-κB1 signaling to immune cell development and growth control (Rouillard 2016, Bui *et al*. 2021). SGSH (N-sulfoglucosamine sulfohydrolase) deficiency impairs heparan sulfate degradation, leading to lysosomal accumulation and compromised skin barrier function, which may exacerbate psoriasis via enhanced inflammation (Magat *et al*. 2022). GCA is a protein-coding gene that encodes grancalcin and is mainly expressed in neutrophils and monocytes/macrophages, which unveiled potential genetic variants that regulate innate immune responses and bridge the realms of psoriasis and lung function (Tanimura *et al*. 2025). COMP (Cartilage oligomeric matrix protein) has been demonstrated to be an indicator for disease activity (bone and cartilage involvement) in patients with psoriasis (Skoumal *et al*. 2008, Bartosinska *et al*. 2015).

From the perspective of biological association and clinical relevance, our colocalization analysis revealed shared genetic loci between several protein/gene targets and psoriasis, indicating potential common genetic structures and pathogenic mechanisms. The enrichment analysis identified several key protein-coding genes involved in critical pathways of psoriasis, such as leukocyte-mediated cytotoxicity, regulation of cell killing, and leukocyte-mediated immunity (Liu *et al*. 2024a, 2024b). Dysregulation of these pathways may trigger excessive immune responses and tissue damage, characteristic of psoriasis (Greb *et al*. 2016). These genes modulate immune cell reactions and cytokine production, highlighting their value as therapeutic targets. We also identified shared molecular pathways between psoriasis and inflammatory bowel disease. This overlap suggests shared therapeutic targets for both diseases, but also highlights the potential for overlapping side effects. Clinically, more than half of the identified targets interact with currently approved anti-psoriasis targets, underscoring their significance for drug discovery. Targeting multiple pathways could enhance treatment outcomes, reduce drug resistance, and minimize side effects, paving the way for innovative, multi-target strategies for psoriasis. Our PPI analysis highlights potential functional connections between novel targets identified in this study and existing anti-psoriasis drug targets. The majority of novel targets exhibit concordant directions with their interacting drug targets, suggesting potential synergistic therapeutic relevance. These results provide an additional layer of evidence for prioritizing targets with both genetic and mechanistic support, and illustrate how integrating expression directionality with PPI networks can guide the selection of high-priority targets for further experimental validation.

Our study possesses several key strengths. First, it represents, to our knowledge, the inaugural implementation of a comprehensive pipeline integrating multi-omics data and multi-level validation for exploring therapeutic targets in psoriasis precision medicine. Second, the robustness of our findings is enhanced by multiple replicated exposures and outcomes that consistently confirmed our results. Third, our study provides a complete chain of evidence, spanning from genetic variation to multi-omics data, functional interpretation, and clinical potential. The comprehensive evidence score, which ranks potential targets, serves as a crucial reference for prioritizing future target development.

However, certain limitations warrant consideration. Despite confirming robust associations through multi-level analyses, inherent limitations of methods such as Mendelian randomization and cohort studies persist. Therefore, future research using diverse designs and methodologies is crucial for further validating our findings. While our multi-omics approach significantly enhances target credibility, additional experimental validation is essential to confirm clinical translatability. Moreover, further population-level evidence is needed, ideally from independent replication cohorts. Future research should also encompass broader experimental studies to investigate potential intervention effects. Another limitation is the tissue-specificity gap between plasma protein levels and local skin biology. To address this, we evaluated cross-tissue consistency from multiple perspectives, including comparison of the effect directions of plasma pQTLs with those of the corresponding skin eQTLs, comparison of SMR results for blood and skin gene expression, and comparison of PGS associations constructed from pQTLs and eQTLs derived from different tissues. We further summarized the direction of effect across all analyses to provide an integrated view of their overall concordance. Overall, several targets showed concordant patterns across plasma, blood, and skin, supporting partial cross-tissue relevance, whereas a smaller subset showed discordant directions across analyses. However, these assessments were limited by the restricted availability of skin-specific QTL resources and the relatively small sample size of GTEx, which reduced both the number of genes that could be examined and the statistical power for cross-tissue comparison. Consequently, plasma-based signals may not fully reflect local skin biology, and some associations may represent systemic rather than skin-specific effects. Future studies with larger and more comprehensive tissue-specific datasets are needed to validate these targets and clarify the relationship between circulating proteins, blood-based molecular signals, and local disease biology.

In summary, our study successfully reveals a comprehensive multi-omic profile of psoriasis and identifies a promising set of potential therapeutic targets. By leveraging these validated protein and gene targets, we offer novel insights into the complex pathology of psoriasis and significantly expand the existing pool of drug discovery targets. This integrated pipeline holds immense promise, and its extension to other complex diseases could further enhance its applicability in precision medicine and accelerate drug development.

## Supplementary Material

btag347_Supplementary_Data

## Data Availability

The pQTL summary data were acquired from previously published studies and can be found in these studies. The SMR-formatted eQTL summary data of GTEx (V8) were acquired from the Yang Lab website (https://yanglab.westlake.edu.cn/software/smr/#eQTLsummarydata). The eQTL summary data of eGTLGen are available at https://eqtlgen.org/phase1.html. The summary-level data of psoriasis were acquired from the FinnGen consortium (finngen_R12_L12_PSORIASIS, https://www.finngen.fi/en), MRC IEU OpenGWAS platform (ebi-a-GCST90019016, https://gwas.mrcieu.ac.uk/), and GWAS Catalog (GCST90476190, https://www.ebi.ac.uk/gwas/). The entire GWAS summary statistics for all proteins are available at https://www.decode.com/summarydata/ and synapse.org/Synapse:syn51824537. The information for drug targets was acquired from the DrugBank (https://go.drugbank.com/) and ChEMBL databases (https://www.ebi.ac.uk/chembl/). The UK Biobank resource could be acquired by submitting an application to the UK Biobank official website (https://www.ukbiobank.ac.uk/). The code used for the analyses in this manuscript has been archived in Zenodo at [DOI: 10.5281/zenodo.19,692,128].
