# Supplementary material for: Integrated multi-omics strategies for identifying novel therapies in psoriasis
Source: Bioinformatics. 2026 May 28;42(6):btag347. doi: 10.1093/bioinformatics/btag347 (PMC13275128; doi:10.1093/bioinformatics/btag347)
Supplement: btag347_Supplementary_Data [file btag347_supplementary_data.zip › Supplement Figures.docx]

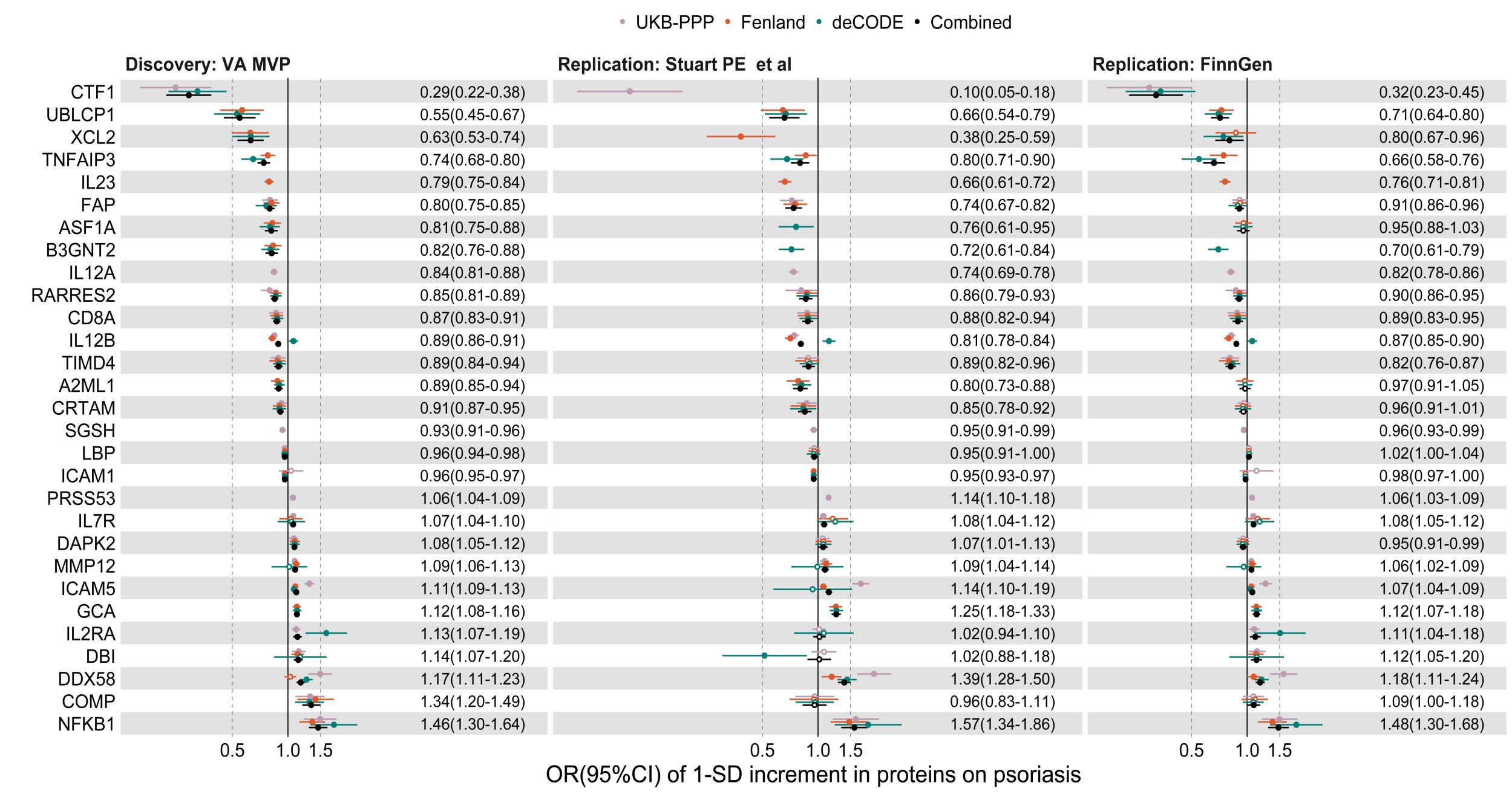


Figure S1. The results of PWAS analysis in the discovery and replication datasets.

The OR (95% CI) was the combined effect of multiple protein data that are available. All of the MR analyses used the index pQTL and the Wald Ratio method. Only proteins that passed the replication analysis are shown. The solid point indicates significant results (*P*<0.05).


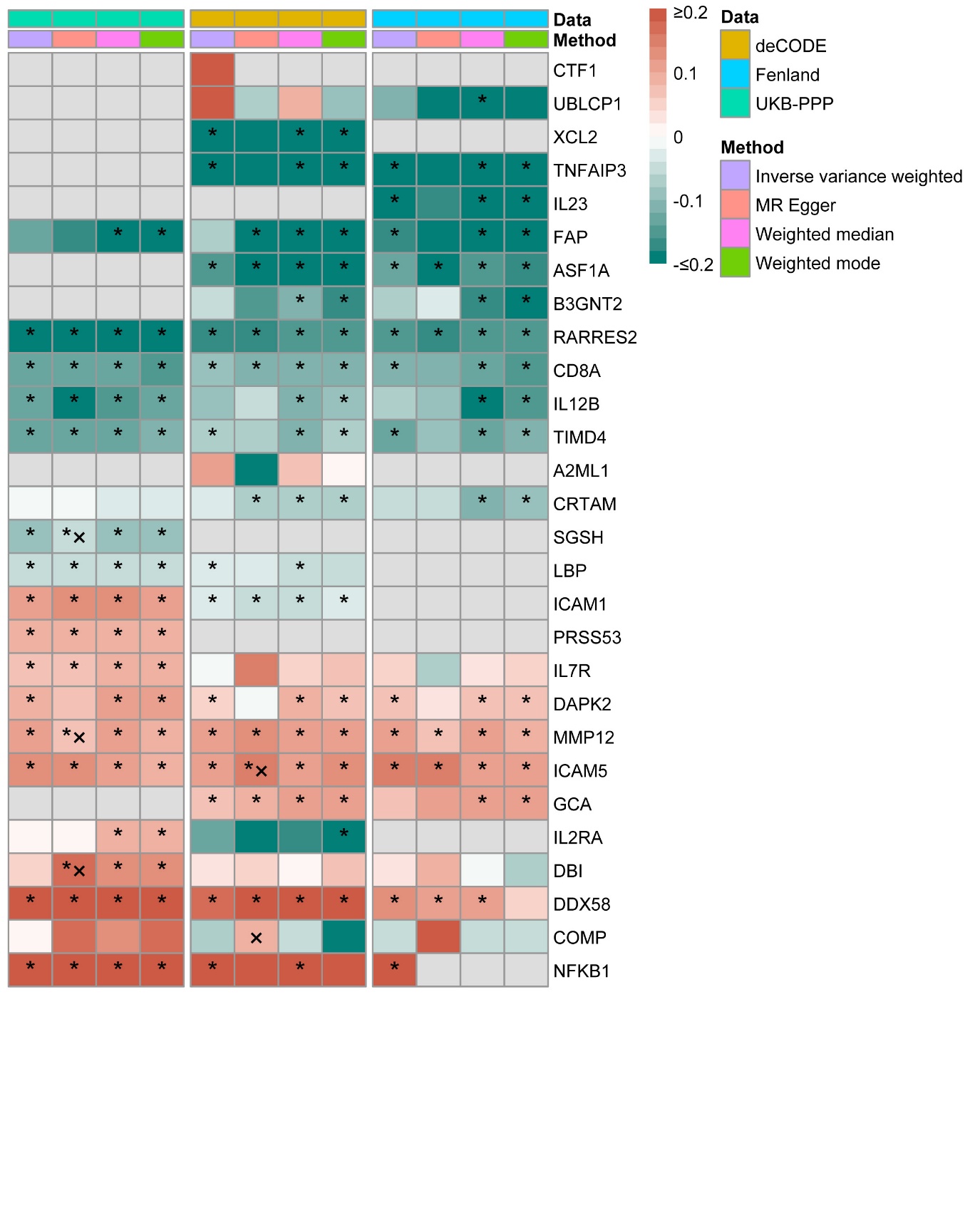


Figure S2. The results of sensitivity analysis using multiple cis-pQTLs and different MR methods.

* *P*<0.05; ×Egger test failed.

MR Egger regression showed consistent results, although some of the results presented horizontal pleiotropy.


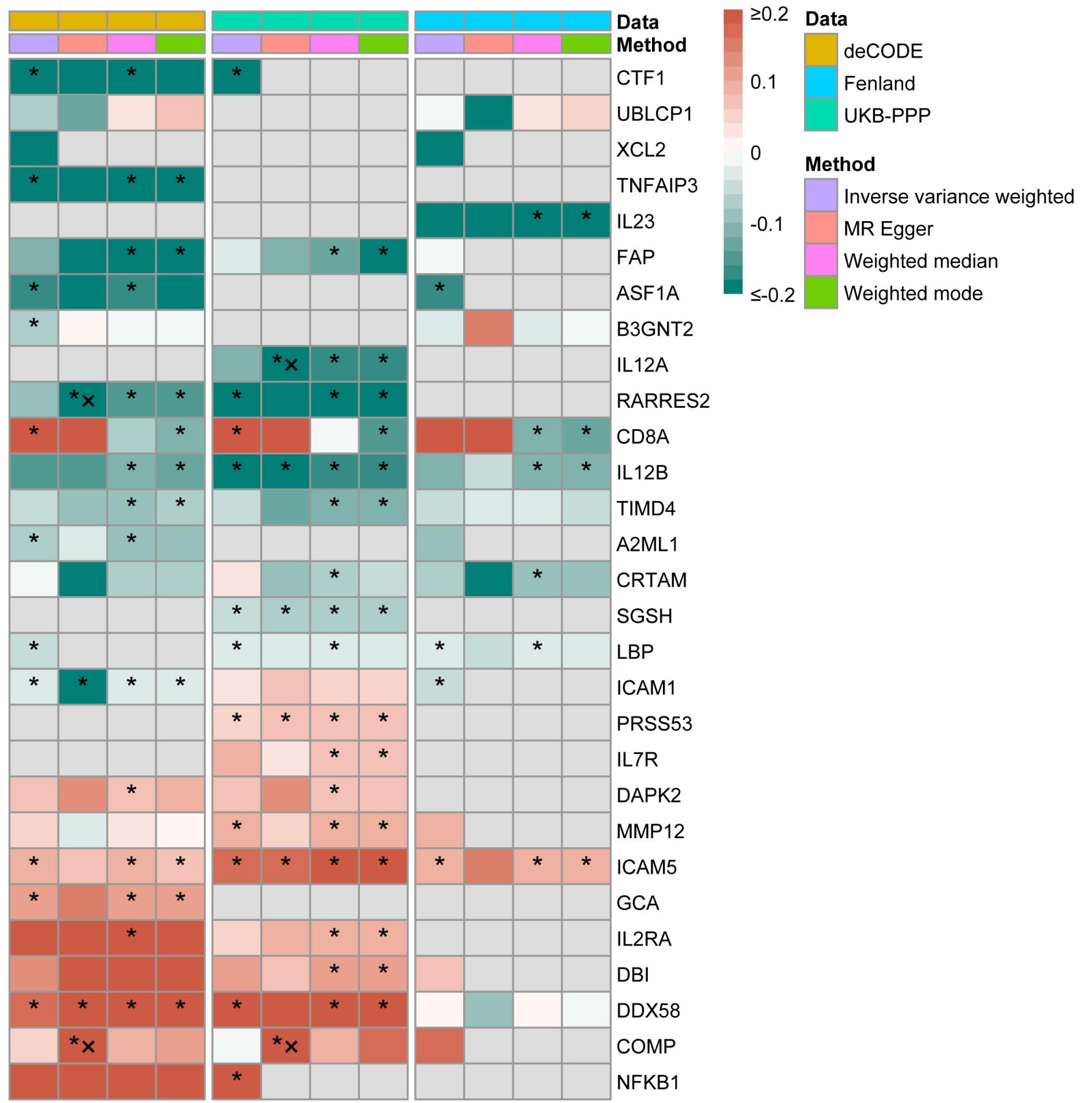


Figure S3. The results of sensitivity analysis using cis+trans pQTLs and different MR methods.

* *P*<0.05; ×Egger test failed.

MR Egger regression showed consistent results, although some of the results presented horizontal pleiotropy.


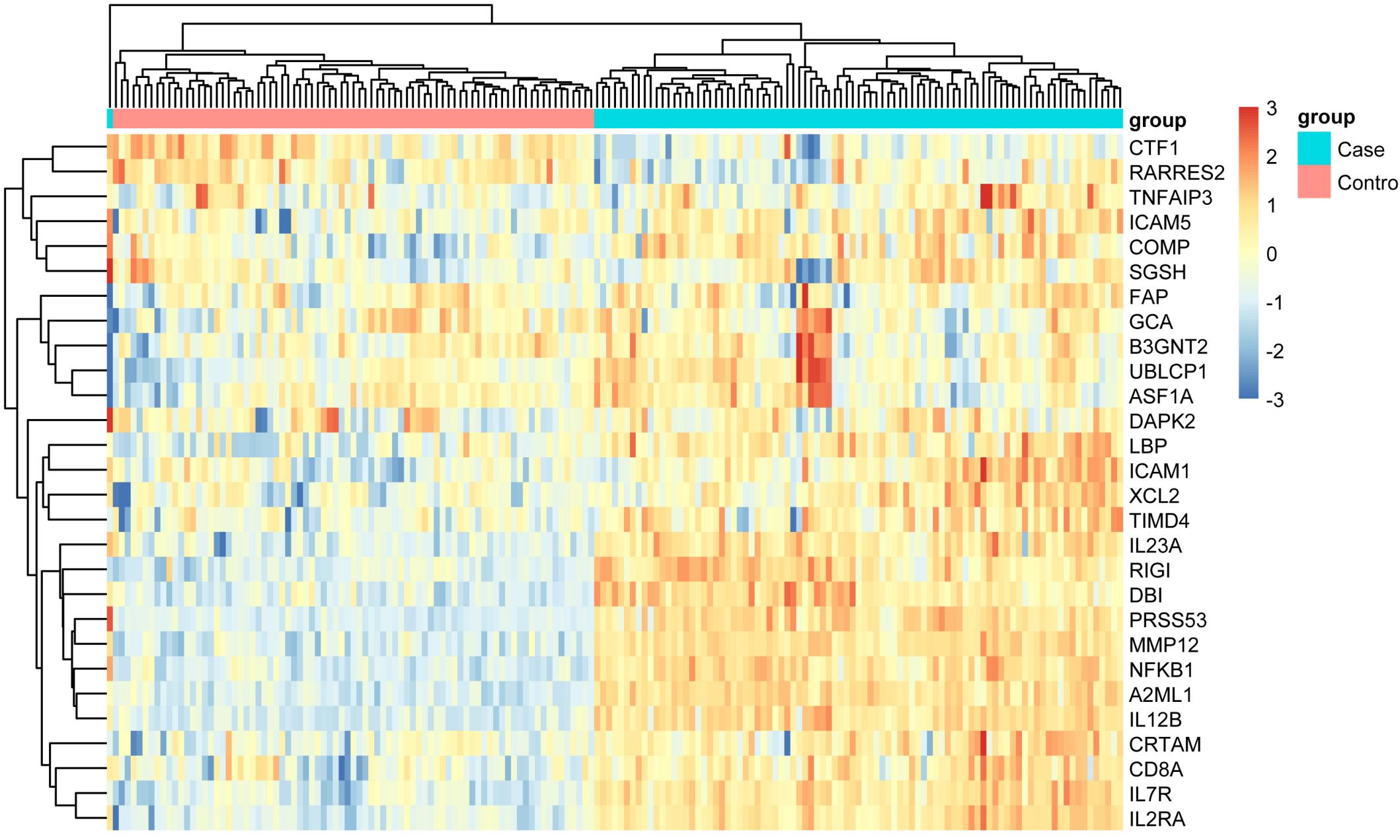


Figure S4. Hierarchical clustering analysis based on identified potential targets.


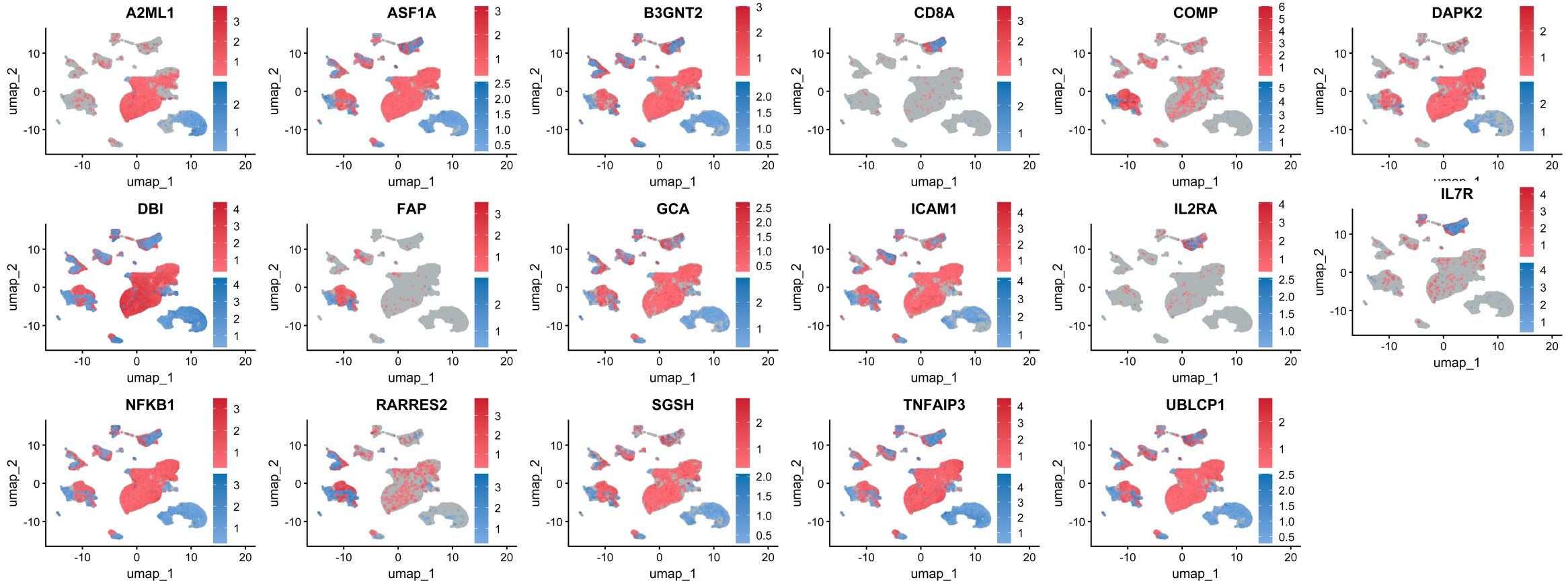


Figure S5. UMAP plot for the expression of each target gene of 17 coding genes across different cell types.

The cells of psoriasis cases are marked with red and the healthy samples are marked with blue.
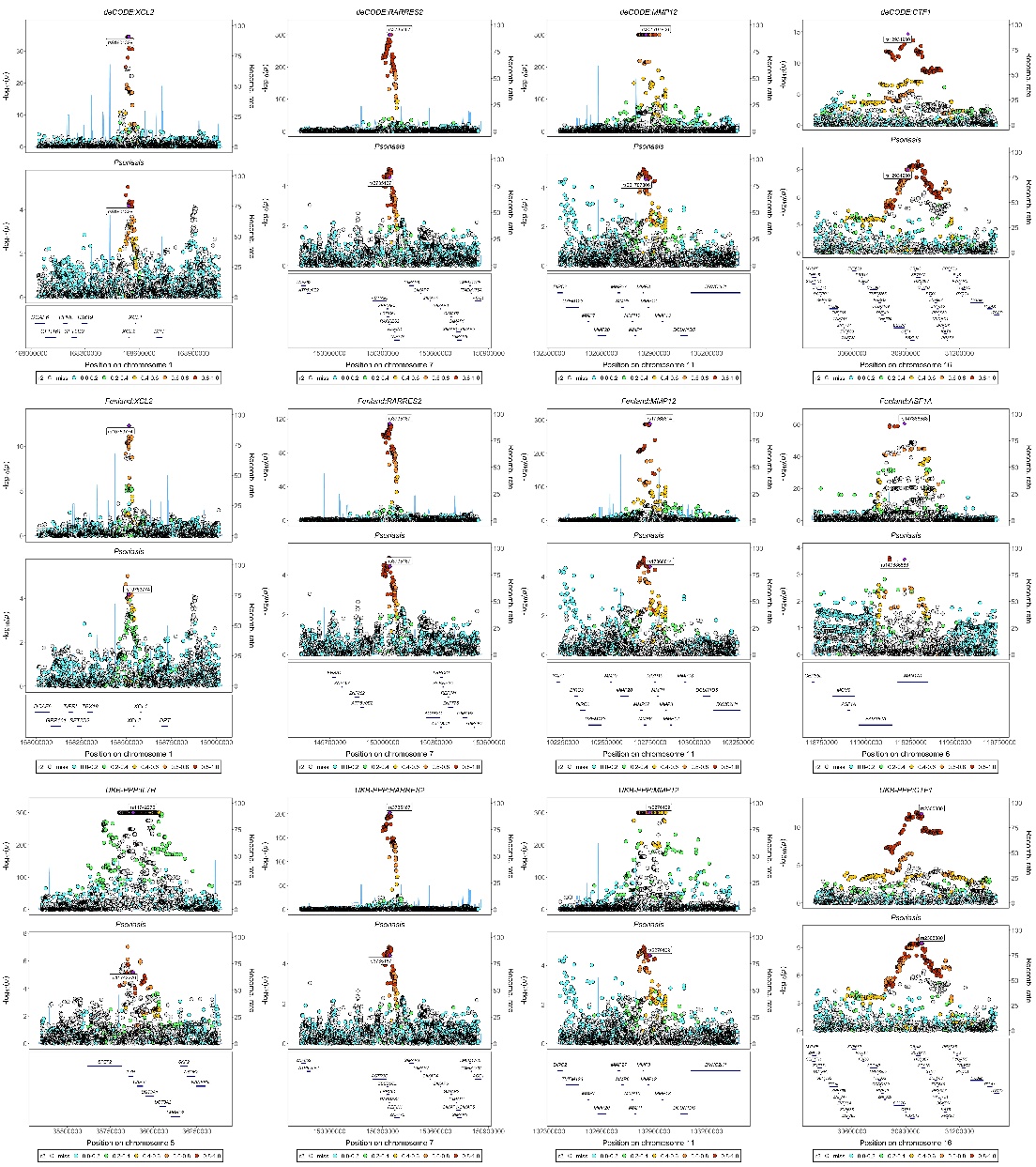


Figure S6. The regional colocalization plot for the potential protein targets.


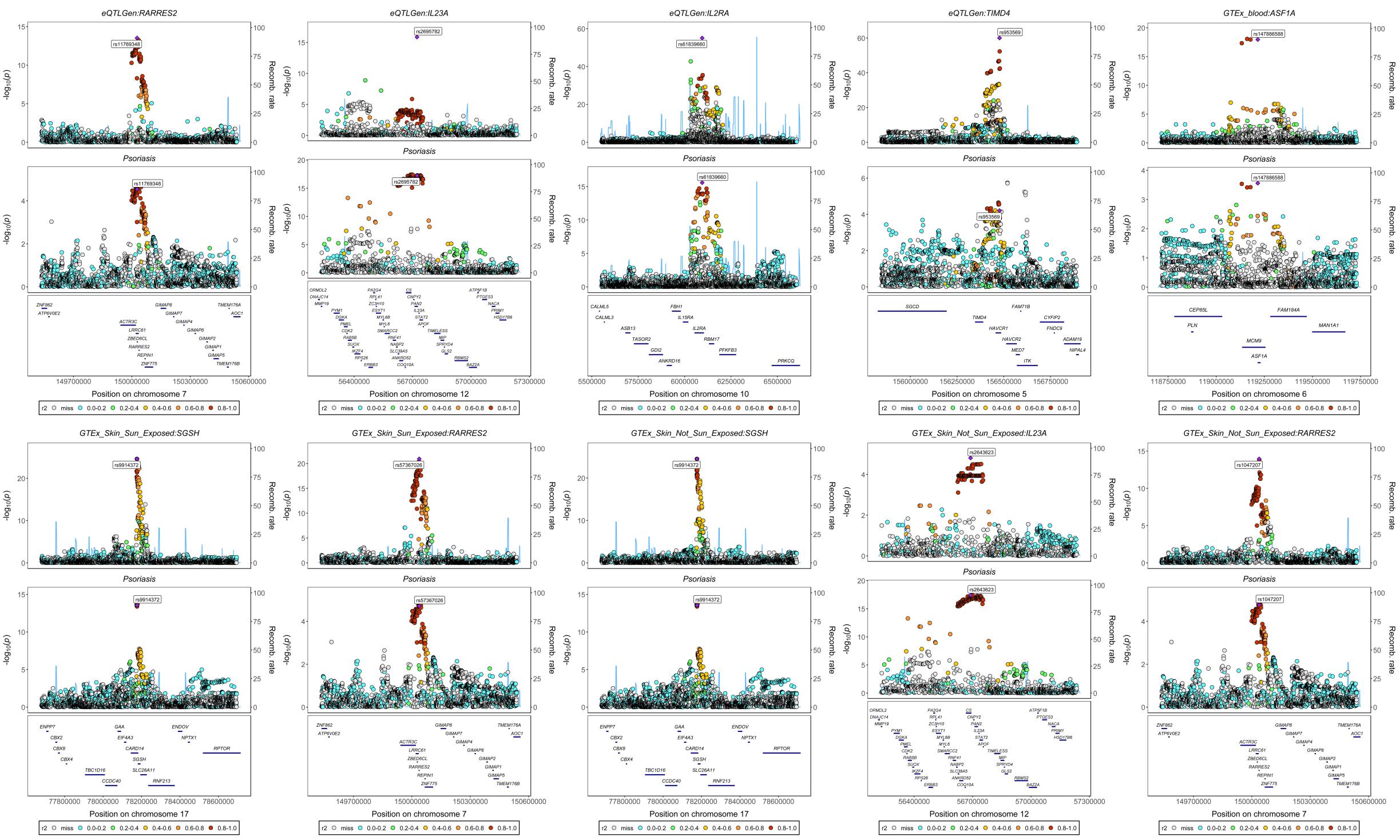


Figure S7. The regional colocalization plot for the gene expression.
