# Supplementary material for: Integrated multi-omics strategies for identifying novel therapies in psoriasis
Source: Bioinformatics. 2026 May 28;42(6):btag347. doi: 10.1093/bioinformatics/btag347 (PMC13275128; doi:10.1093/bioinformatics/btag347)
Supplement: btag347_Supplementary_Data [file btag347_supplementary_data.zip › Supplement Tables.docx]

**Table S1. The pQTLs and the genetic associations with blood proteome.**

| **Protein** | **Fullname** | **SNP** | **chr** | **pos** | **A1** | **A2** | **eaf** | **beta** | **pval** | **data** |
| --- | --- | --- | --- | --- | --- | --- | --- | --- | --- | --- |
| A2ML1 | Alpha-2-macroglobulin-like protein 1 | rs1558526 | 12 | 8857224 | A | G | 0.2182 | -0.37 | 0 | deCODE |
| CD8A | T-cell surface glycoprotein CD8 alpha chain | rs3020726 | 2 | 86789383 | G | A | 0.1952 | 0.4 | 0 | deCODE |
| DAPK2 | Death-associated protein kinase 2 | rs55986634 | 15 | 63983446 | A | G | 0.4172 | 0.33 | 0 | deCODE |
| GCA | Grancalcin | rs17783344 | 2 | 162352383 | G | T | 0.0955 | -0.57 | 0 | deCODE |
| IL12B | Interleukin-12 subunit beta | rs3213120 | 5 | 159316100 | T | C | 0.025 | -1.16 | 0 | deCODE |
| LBP | Lipopolysaccharide-binding protein | rs2232613 | 20 | 38369011 | T | C | 0.0987 | -1.03 | 0 | deCODE |
| ICAM1 | Intercellular adhesion molecule 1 | rs5498 | 19 | 10285007 | G | A | 0.4663 | -1.2 | 0 | deCODE |
| ICAM5 | Intercellular adhesion molecule 5 | rs281439 | 19 | 10289434 | G | C | 0.2087 | -0.86 | 0 | deCODE |
| RARRES2 | Retinoic acid receptor responder protein 2 | rs3735167 | 7 | 150342466 | T | C | 0.2609 | 0.34 | 2.7542E-313 | deCODE |
| DDX58 | Probable ATP-dependent RNA helicase DDX58 | rs1133071 | 9 | 32455676 | G | A | 0.2749 | 0.3 | 4.8978E-262 | deCODE |
| TIMD4 | T-cell immunoglobulin and mucin domain-containing protein 4 | rs4704826 | 5 | 156965071 | C | A | 0.358 | 0.27 | 1.2589E-218 | deCODE |
| COMP | Cartilage oligomeric matrix protein | rs12974746 | 19 | 18795739 | G | A | 0.0377 | -0.62 | 1.9498E-181 | deCODE |
| CRTAM | Cytotoxic and regulatory T-cell molecule | rs2370794 | 11 | 122844074 | G | A | 0.3272 | 0.25 | 8.5114E-179 | deCODE |
| FAP | Prolyl endopeptidase FAP | rs10490425 | 2 | 162210947 | T | A | 0.0157 | 0.89 | 7.5858E-158 | deCODE |
| B3GNT2 | ""N-acetyllactosaminide beta-1,3-N-acetylglucosaminyltransferase 2"""""" | rs4073090 | 2 | 62336639 | G | A | 0.447 | 0.2 | 5.6234E-138 | deCODE |
| UBLCP1 | Ubiquitin-like domain-containing CTD phosphatase 1 | rs13171129 | 5 | 159306365 | A | C | 0.0303 | -0.56 | 7.4131E-128 | deCODE |
| ASF1A | Histone chaperone ASF1A | rs147531464 | 6 | 118839328 | T | C | 0.1642 | 0.24 | 2.8184E-114 | deCODE |
| TNFAIP3 | Tumor necrosis factor alpha-induced protein 3 | rs6932056 | 6 | 137921300 | C | T | 0.025 | 0.47 | 1.90546E-79 | deCODE |
| IL7R | Interleukin-7 receptor subunit alpha | rs6451229 | 5 | 35866116 | G | A | 0.345 | 0.14 | 2.69153E-65 | deCODE |
| MMP12 | Macrophage metalloelastase | rs74926032 | 11 | 102877643 | G | A | 0.0616 | 0.28 | 1.54882E-62 | deCODE |
| XCL2 | Cytokine SCM-1 beta | rs61801328 | 1 | 168543240 | A | T | 0.1884 | 0.12 | 3.89045E-35 | deCODE |
| IL2RA | Interleukin-2 receptor subunit alpha | rs7093069 | 10 | 6021356 | T | C | 0.2431 | -0.11 | 1.86209E-34 | deCODE |
| NFKB1 | Nuclear factor NF-kappa-B p105 subunit | rs230508 | 4 | 102558122 | A | G | 0.3431 | 0.08 | 4.46684E-24 | deCODE |
| DBI | Acyl-CoA-binding protein | rs4849777 | 2 | 119365632 | C | A | 0.2504 | 0.09 | 6.91831E-24 | deCODE |
| CTF1 | Cardiotrophin-1 | rs12934900 | 16 | 30912281 | A | T | 0.3565 | -0.06 | 2.13796E-15 | deCODE |
| CD8A | T-cell surface glycoprotein CD8 alpha chain | rs3020726 | 2 | 86789383 | G | A | 0.16087 | 0.353 | 0 | UKB-PPP |
| CRTAM | Cytotoxic and regulatory T-cell molecule | rs2370794 | 11 | 122844074 | G | A | 0.33384 | 0.326 | 0 | UKB-PPP |
| DAPK2 | Death-associated protein kinase 2 | rs55986634 | 15 | 63983446 | A | G | 0.42851 | 0.378 | 0 | UKB-PPP |
| DBI | Acyl-CoA-binding protein | rs11680074 | 2 | 119384247 | A | G | 0.20393 | -0.319 | 0 | UKB-PPP |
| ICAM5 | Intercellular adhesion molecule 5 | rs75407602 | 19 | 10286554 | T | C | 0.0486 | -0.943 | 0 | UKB-PPP |
| IL12A |  | rs4244437 | 5 | 159346109 | G | A | 0.32189 | -0.552 | 0 | UKB-PPP |
| IL12B | Interleukin-12 subunit beta | rs4244437 | 5 | 159346109 | G | A | 0.32189 | -0.568 | 0 | UKB-PPP |
| IL2RA | Interleukin-2 receptor subunit alpha | rs7093069 | 10 | 6021356 | T | C | 0.18662 | -0.514 | 0 | UKB-PPP |
| IL7R | Interleukin-7 receptor subunit alpha | rs11742270 | 5 | 35881341 | A | G | 0.27467 | -0.852 | 0 | UKB-PPP |
| LBP | Lipopolysaccharide-binding protein | rs2232613 | 20 | 38369011 | T | C | 0.08258 | -1.199 | 0 | UKB-PPP |
| MMP12 | Macrophage metalloelastase | rs17368814 | 11 | 102877965 | G | A | 0.1239 | -0.81 | 0 | UKB-PPP |
| PRSS53 | Serine protease 53 | rs4468641 | 16 | 31085555 | C | A | 0.38489 | 0.891 | 0 | UKB-PPP |
| SGSH | N-sulphoglucosamine sulphohydrolase | rs7503034 | 17 | 80210594 | T | C | 0.33041 | -0.9 | 0 | UKB-PPP |
| TIMD4 | T-cell immunoglobulin and mucin domain-containing protein 4 | rs12657266 | 5 | 156968992 | C | T | 0.36441 | 0.234 | 1.7378E-262 | UKB-PPP |
| FAP | Prolyl endopeptidase FAP | rs16846386 | 2 | 162215216 | A | G | 0.01017 | 1.074 | 1E-239 | UKB-PPP |
| COMP | Cartilage oligomeric matrix protein | rs12974746 | 19 | 18795739 | G | A | 0.03119 | -0.605 | 1.9953E-218 | UKB-PPP |
| RARRES2 | Retinoic acid receptor responder protein 2 | rs3735167 | 7 | 150342466 | T | C | 0.25938 | 0.226 | 5.0119E-203 | UKB-PPP |
| ICAM1 | Intercellular adhesion molecule 1 | rs139053442 | 19 | 10283720 | C | G | 0.00156 | -2.19 | 1.9953E-157 | UKB-PPP |
| DDX58 | Antiviral innate immune response receptor RIG-I | rs11795343 | 9 | 32523739 | C | T | 0.4018 | -0.152 | 5.6234E-113 | UKB-PPP |
| NFKB1 | Nuclear factor NF-kappa-B p105 subunit | rs2272676 | 4 | 102502169 | T | G | 0.34989 | 0.117 | 1.05439E-63 | UKB-PPP |
| CTF1 | Cardiotrophin-1 | rs2054213 | 16 | 30960489 | A | G | 0.37437 | -0.049 | 9.72747E-13 | UKB-PPP |
| LBP | Lipopolysaccharide-binding protein | rs2232613 | 20 | 36997655 | T | C | 0.0761 | -1.2145 | 0 | Fenland |
| ICAM1 | Intercellular adhesion molecule 1 | rs5498 | 19 | 10395683 | A | G | 0.5758 | 1.1941 | 0 | Fenland |
| ICAM5 | Intercellular adhesion molecule 5 | rs281439 | 19 | 10400110 | C | G | 0.7742 | 0.6908 | 0 | Fenland |
| MMP12 | Macrophage metalloelastase | rs17368814 | 11 | 102748695 | A | G | 0.8773 | 0.6627 | 2.4E-289 | Fenland |
| IL12B | Interleukin-12 subunit beta | rs4244437 | 5 | 158773117 | A | G | 0.687 | 0.4884 | 5.85E-286 | Fenland |
| GCA | Grancalcin | rs17783344 | 2 | 163208893 | T | G | 0.8561 | 0.586 | 6.54E-207 | Fenland |
| IL23 | Interleukin-23 | rs4244437 | 5 | 158773117 | A | G | 0.687 | 0.4083 | 2.86E-197 | Fenland |
| DAPK2 | Death-associated protein kinase 2 | rs4436737 | 15 | 64283076 | A | G | 0.4214 | 0.3351 | 2.48E-150 | Fenland |
| DDX58 | Probable ATP-dependent RNA helicase DDX58 | rs7025666 | 9 | 32533829 | T | C | 0.7363 | 0.3629 | 1.67E-132 | Fenland |
| DBI | Acyl-CoA-binding protein | rs6733335 | 2 | 120142939 | C | G | 0.7976 | 0.3549 | 5.27E-125 | Fenland |
| RARRES2 | Retinoic acid receptor responder protein 2 | rs3735167 | 7 | 150039555 | T | C | 0.2593 | 0.3405 | 5.54E-115 | Fenland |
| CD8A | T-cell surface glycoprotein CD8 alpha chain | rs3020726 | 2 | 87016506 | A | G | 0.8415 | -0.3692 | 1.7E-100 | Fenland |
| A2ML1 | Alpha-2-macroglobulin-like protein 1 | rs1558526 | 12 | 9009820 | A | G | 0.2595 | -0.3039 | 9.5E-90 | Fenland |
| TNFAIP3 | Tumor necrosis factor alpha-induced protein 3 | rs59693083 | 6 | 138186532 | A | G | 0.9179 | -0.4334 | 3.47E-77 | Fenland |
| CRTAM | Cytotoxic and regulatory T-cell molecule | rs2370794 | 11 | 122714782 | A | G | 0.6724 | -0.255 | 1.62E-76 | Fenland |
| FAP | Prolyl endopeptidase FAP | rs16846387 | 2 | 163073082 | T | C | 0.9898 | -1.1219 | 3.69E-64 | Fenland |
| ASF1A | Histone chaperone ASF1A | rs147886588 | 6 | 119215902 | C | G | 0.8292 | -0.2808 | 3.3E-61 | Fenland |
| B3GNT2 | N-acetyllactosaminide beta-1,3-N-acetylglucosaminyltransferase 2 | rs112392179 | 2 | 62567995 | D | I | 0.5835 | -0.2155 | 1.84E-56 | Fenland |
| TIMD4 | T-cell immunoglobulin and mucin domain-containing protein 4 | rs12657266 | 5 | 156396003 | T | C | 0.6349 | -0.217 | 1.05E-54 | Fenland |
| IL7R | Interleukin-7 receptor subunit alpha | rs6451229 | 5 | 35866218 | A | G | 0.5932 | -0.1647 | 4.83E-34 | Fenland |
| COMP | Cartilage oligomeric matrix protein | rs12974746 | 19 | 18906548 | A | G | 0.9708 | 0.4862 | 2.78E-30 | Fenland |
| NFKB1 | Nuclear factor NF-kappa-B p105 subunit | rs62328536 | 4 | 103399049 | A | C | 0.3439 | 0.1476 | 1.71E-28 | Fenland |
| UBLCP1 | Ubiquitin-like domain-containing CTD phosphatase 1 | rs71593333 | 5 | 158690030 | A | G | 0.0113 | -0.6262 | 2.09E-21 | Fenland |
| XCL2 | Cytokine SCM-1 beta | rs10753774 | 1 | 168514008 | T | C | 0.2987 | 0.1019 | 5.25E-13 | Fenland |

**Table S2. The pQTLs and the genetic associations with both exposure and outcome.**

| **Protein** | **SNP** | **EA** | **NEA** | **beta.exposure** | **beta.outcome** | **se.exposure** | **se.outcome** | **pval.exposure** | **pval.outcome** | **dat** |
| --- | --- | --- | --- | --- | --- | --- | --- | --- | --- | --- |
| DDX58 | rs1133071 | G | A | 0.3 | 0.1089 | 0.008675315 | 0.0181 | 4.8978E-262 | 1.62401E-09 | Iceland |
| GCA | rs17783344 | G | T | -0.57 | -0.1311 | 0.013500797 | 0.0241 | 1E-300 | 5.21399E-08 | Iceland |
| IL12B | rs3213120 | T | C | -1.16 | -0.159 | 0.02635702 | 0.0482 | 1E-300 | 0.000962299 | Iceland |
| TNFAIP3 | rs6932056 | C | T | 0.47 | -0.1814 | 0.024903432 | 0.0499 | 1.90546E-79 | 0.000277198 | Iceland |
| TIMD4 | rs4704826 | C | A | 0.27 | -0.0289 | 0.008554809 | 0.0166 | 1.2589E-218 | 0.0817203 | Iceland |
| CD8A | rs3020726 | G | A | 0.4 | -0.0481 | 0.010103941 | 0.0228 | 1E-300 | 0.0348602 | Iceland |
| A2ML1 | rs1558526 | A | G | -0.37 | 0.075 | 0.009440737 | 0.0223 | 1E-300 | 0.000763502 | Iceland |
| DBI | rs4849777 | C | A | 0.09 | -0.06 | 0.008930424 | 0.0242 | 6.91831E-24 | 0.0131199 | Iceland |
| ASF1A | rs147531464 | T | C | 0.24 | -0.0657 | 0.010563209 | 0.0268 | 2.8184E-114 | 0.0140501 | Iceland |
| UBLCP1 | rs13171129 | A | C | -0.56 | 0.2244 | 0.023280053 | 0.075 | 7.4131E-128 | 0.00277799 | Iceland |
| LBP | rs2232613 | T | C | -1.03 | 0.0555 | 0.013107357 | 0.044 | 1E-300 | 0.2077 | Iceland |
| RARRES2 | rs3735167 | T | C | 0.34 | -0.0474 | 0.008985807 | 0.0225 | 1E-300 | 0.0349704 | Iceland |
| IL2RA | rs7093069 | T | C | -0.11 | -0.0079 | 0.008985717 | 0.0209 | 1.86209E-34 | 0.7055 | Iceland |
| ICAM1 | rs5498 | G | A | -1.2 | 0.0644 | 0.006868397 | 0.0162 | 1E-300 | 6.69206E-05 | Iceland |
| DAPK2 | rs55986634 | A | G | 0.33 | 0.0223 | 0.007938434 | 0.017 | 1E-300 | 0.1877 | Iceland |
| MMP12 | rs74926032 | G | A | 0.28 | -0.0023 | 0.016776478 | 0.0464 | 1.54882E-62 | 0.9602 | Iceland |
| IL7R | rs6451229 | G | A | 0.14 | 0.0301 | 0.008203798 | 0.0163 | 2.69153E-65 | 0.0641195 | Iceland |
| ICAM5 | rs114170067 | T | C | 0.16 | -0.0107 | 0.018025731 | 0.0401 | 6.91831E-19 | 0.7888 | Iceland |
| CRTAM | rs2370794 | G | A | 0.25 | -0.0463 | 0.008768487 | 0.0208 | 8.5114E-179 | 0.02573 | Iceland |
| B3GNT2 | rs4073090 | G | A | 0.2 | -0.0662 | 0.007998933 | 0.0162 | 5.6234E-138 | 4.66498E-05 | Iceland |
| COMP | rs12974746 | G | A | -0.62 | 0.0248 | 0.021585225 | 0.0752 | 1.9498E-181 | 0.742 | Iceland |
| NFKB1 | rs230508 | A | G | 0.08 | 0.0501 | 0.007904502 | 0.017 | 4.46684E-24 | 0.00316898 | Iceland |
| CTF1 | rs2054213 | A | G | -0.049 | 0.115 | 0.00686822 | 0.0164 | 9.72747E-13 | 2.71706E-12 | UKB-PPP |
| ICAM5 | rs75407602 | T | C | -0.943 | -0.5033 | 0.015118818 | 0.052 | 1E-300 | 3.53102E-22 | UKB-PPP |
| IL2RA | rs7093069 | T | C | -0.514 | -0.0079 | 0.008558224 | 0.0209 | 1E-300 | 0.7055 | UKB-PPP |
| FAP | rs16846386 | A | G | 1.074 | -0.3504 | 0.032483092 | 0.0799 | 1E-239 | 0.00001149 | UKB-PPP |
| LBP | rs2232613 | T | C | -1.199 | 0.0555 | 0.011697592 | 0.044 | 1E-300 | 0.2077 | UKB-PPP |
| RARRES2 | rs3735167 | T | C | 0.226 | -0.0474 | 0.007433474 | 0.0225 | 5.0119E-203 | 0.0349704 | UKB-PPP |
| TIMD4 | rs12657266 | C | T | 0.234 | -0.0285 | 0.006760895 | 0.0166 | 1.7378E-262 | 0.0853906 | UKB-PPP |
| COMP | rs12974746 | G | A | -0.605 | 0.0248 | 0.019177968 | 0.0752 | 1.9953E-218 | 0.742 | UKB-PPP |
| IL12B | rs4244437 | G | A | -0.568 | 0.1686 | 0.006565434 | 0.0169 | 1E-300 | 1.98518E-23 | UKB-PPP |
| CRTAM | rs2370794 | G | A | 0.326 | -0.0463 | 0.006912935 | 0.0208 | 1E-300 | 0.02573 | UKB-PPP |
| CD8A | rs3020726 | G | A | 0.353 | -0.0481 | 0.008738532 | 0.0228 | 1E-300 | 0.0348602 | UKB-PPP |
| IL7R | rs11742270 | A | G | -0.852 | -0.0568 | 0.006575562 | 0.0183 | 1E-300 | 0.001869 | UKB-PPP |
| DBI | rs11680074 | A | G | -0.319 | -0.0238 | 0.00803586 | 0.025 | 1E-300 | 0.3406 | UKB-PPP |
| DDX58 | rs11795343 | C | T | -0.152 | -0.1062 | 0.00672909 | 0.0164 | 5.6234E-113 | 1.02499E-10 | UKB-PPP |
| IL12A | rs4244437 | G | A | -0.552 | 0.1686 | 0.00659951 | 0.0169 | 1E-300 | 1.98518E-23 | UKB-PPP |
| MMP12 | rs17368814 | G | A | -0.81 | -0.0667 | 0.009375665 | 0.0254 | 1E-300 | 0.00854594 | UKB-PPP |
| NFKB1 | rs2272676 | T | G | 0.117 | 0.0552 | 0.006943742 | 0.0171 | 1.05439E-63 | 0.00126701 | UKB-PPP |
| SGSH | rs7503034 | T | C | -0.9 | 0.048 | 0.005919489 | 0.0206 | 1E-300 | 0.0199802 | UKB-PPP |
| DAPK2 | rs55986634 | A | G | 0.378 | 0.0223 | 0.006425796 | 0.017 | 1E-300 | 0.1877 | UKB-PPP |
| PRSS53 | rs4468641 | C | A | 0.891 | 0.1181 | 0.005538383 | 0.0164 | 1E-300 | 5.92789E-13 | UKB-PPP |
| IL23 | rs4244437 | A | G | 0.4083 | -0.1686 | 0.0136 | 0.0169 | 2.86E-197 | 1.98518E-23 | Fenland |
| DDX58 | rs7025666 | T | C | 0.3629 | 0.0617 | 0.0148 | 0.0225 | 1.67E-132 | 0.00623893 | Fenland |
| GCA | rs17783344 | T | G | 0.586 | 0.1311 | 0.0191 | 0.0241 | 6.54E-207 | 5.21399E-08 | Fenland |
| IL12B | rs4244437 | A | G | 0.4884 | -0.1686 | 0.0135 | 0.0169 | 5.85E-286 | 1.98518E-23 | Fenland |
| TNFAIP3 | rs59693083 | A | G | -0.4334 | 0.0662 | 0.0233 | 0.0303 | 3.47E-77 | 0.0286497 | Fenland |
| TIMD4 | rs12657266 | T | C | -0.217 | 0.0285 | 0.0139 | 0.0166 | 1.05E-54 | 0.0853906 | Fenland |
| CD8A | rs3020726 | A | G | -0.3692 | 0.0481 | 0.0173 | 0.0228 | 1.7E-100 | 0.0348602 | Fenland |
| A2ML1 | rs1558526 | A | G | -0.3039 | 0.075 | 0.0151 | 0.0223 | 9.5E-90 | 0.000763502 | Fenland |
| XCL2 | rs10753774 | T | C | 0.1019 | -0.098 | 0.0141 | 0.0222 | 5.25E-13 | 1.02499E-05 | Fenland |
| UBLCP1 | rs71593333 | A | G | -0.6262 | 0.2748 | 0.0659 | 0.0873 | 2.09E-21 | 0.00164999 | Fenland |
| LBP | rs2232613 | T | C | -1.2145 | 0.0555 | 0.0227 | 0.044 | 1E-300 | 0.2077 | Fenland |
| RARRES2 | rs3735167 | T | C | 0.3405 | -0.0474 | 0.0149 | 0.0225 | 5.54E-115 | 0.0349704 | Fenland |
| ICAM1 | rs5498 | A | G | 1.1941 | -0.0644 | 0.0075 | 0.0162 | 1E-300 | 6.69206E-05 | Fenland |
| DAPK2 | rs4436737 | A | G | 0.3351 | 0.0228 | 0.0128 | 0.017 | 2.48E-150 | 0.1785 | Fenland |
| MMP12 | rs17368814 | A | G | 0.6627 | 0.0667 | 0.0182 | 0.0254 | 2.4E-289 | 0.00854594 | Fenland |
| FAP | rs16846387 | T | C | -1.1219 | 0.3169 | 0.0663 | 0.084 | 3.69E-64 | 0.000160398 | Fenland |
| IL7R | rs6451229 | A | G | -0.1647 | -0.0301 | 0.0135 | 0.0163 | 4.83E-34 | 0.0641195 | Fenland |
| CRTAM | rs2370794 | A | G | -0.255 | 0.0463 | 0.0138 | 0.0208 | 1.62E-76 | 0.02573 | Fenland |
| COMP | rs12974746 | A | G | 0.4862 | -0.0248 | 0.0425 | 0.0752 | 2.78E-30 | 0.742 | Fenland |
| ICAM5 | rs281440 | A | G | 0.8329 | 0.0554 | 0.0143 | 0.0193 | 1E-300 | 0.00421502 | Fenland |
| NFKB1 | rs62328536 | A | C | 0.1476 | 0.0575 | 0.0133 | 0.0174 | 1.71E-28 | 0.000971404 | Fenland |

**Table S3. The results of SMR analysis in tissue-specific gene expression data.**

| **Gene** | **topSNP** | **A1** | **A2** | **b_SMR** | **se_SMR** | **p_SMR** | **p_HEIDI** | **eQTL data** | **tissue** | **outcome data** |
| --- | --- | --- | --- | --- | --- | --- | --- | --- | --- | --- |
| TNFAIP3 | rs498361 | C | T | 0.824705 | 0.233141 | 0.000404132 | 0.2384535 | eQTLGen | Blood | VA MVP |
| IL23A | rs2695782 | C | T | -1.47059 | 0.246273 | 2.35265E-09 | 0.0379137 | eQTLGen | Blood | VA MVP |
| ASF1A | rs147531464 | T | C | -0.114722 | 0.032409 | 0.000400411 | 0.6112356 | eQTLGen | Blood | VA MVP |
| ASF1A | rs147531464 | T | C | -0.215021 | 0.0655042 | 0.001028701 | 0.5951578 | GTEx | Blood | VA MVP |
| IL12A | rs4680536 | G | A | 0.0667191 | 0.23116 | 0.7728661 | 0.5533491 | eQTLGen | Blood | VA MVP |
| RARRES2 | rs11769348 | C | T | -0.727609 | 0.198647 | 0.000249453 | 0.7852941 | eQTLGen | Blood | VA MVP |
| TIMD4 | rs953569 | G | T | 0.344195 | 0.0884307 | 9.93161E-05 | 0.1299822 | eQTLGen | Blood | VA MVP |
| SGSH | rs9894254 | T | C | 0.187273 | 0.090973 | 0.03953726 | 0.01391461 | GTEx | Blood | VA MVP |
| PRSS53 | rs7199949 | C | G | -0.426239 | 0.112198 | 0.000145279 | 0.5935567 | GTEx | Blood | VA MVP |
| ICAM5 | rs73510898 | A | G | -0.340176 | 0.0893024 | 0.000139382 | 0.1703636 | GTEx | Blood | VA MVP |
| DBI | rs12612942 | G | A | 0.131892 | 0.0377733 | 0.000480013 | 0.2762953 | eQTLGen | Blood | VA MVP |
| TNFAIP3 | rs498361 | C | T | 1.51454 | 0.364362 | 3.22886E-05 | 0.1588241 | eQTLGen | Blood | Stuart PE et al |
| IL23A | rs2695782 | C | T | -2.28074 | 0.373824 | 1.05339E-09 | 0.0329171 | eQTLGen | Blood | Stuart PE et al |
| ASF1A | rs147531464 | T | C | -0.138328 | 0.0564958 | 0.01434687 | 0.1721943 | eQTLGen | Blood | Stuart PE et al |
| ASF1A | rs147531464 | T | C | -0.250564 | 0.106057 | 0.0181494 | 0.765557 | GTEx | Blood | Stuart PE et al |
| IL12A | rs4680536 | G | A | 0.568246 | 0.282909 | 0.04458153 | 0.4861327 | eQTLGen | Blood | Stuart PE et al |
| RARRES2 | rs11769348 | C | T | -0.801038 | 0.332583 | 0.01601675 | 0.1123983 | eQTLGen | Blood | Stuart PE et al |
| TIMD4 | rs953569 | G | T | 0.337511 | 0.15292 | 0.02730664 | 0.911631 | eQTLGen | Blood | Stuart PE et al |
| SGSH | rs114747872 | A | G | 0.159322 | 0.0732586 | 0.0296465 | 0.06419896 | eQTLGen | Blood | Stuart PE et al |
| SGSH | rs9894254 | T | C | 0.27161 | 0.155453 | 0.08060029 | 0.3573759 | GTEx | Blood | Stuart PE et al |
| ICAM1 | rs281437 | T | C | -0.473189 | 0.143075 | 0.000942092 | 0.01177429 | GTEx | Blood | Stuart PE et al |
| PRSS53 | rs7199949 | C | G | -0.881739 | 0.196183 | 6.97456E-06 | 0.9257878 | GTEx | Blood | Stuart PE et al |
| ICAM5 | rs73510898 | A | G | -0.6547 | 0.152885 | 1.84981E-05 | 0.6035105 | GTEx | Blood | Stuart PE et al |
| IL2RA | rs61839660 | T | C | 0.406411 | 0.121109 | 0.00079149 | 0.2313399 | eQTLGen | Blood | Stuart PE et al |
| DBI | rs12612942 | G | A | 0.0515576 | 0.0681309 | 0.4492031 | 0.4571053 | eQTLGen | Blood | Stuart PE et al |
| NFKB1 | rs11097787 | T | C | 0.261017 | 0.0919416 | 0.00452629 | 0.09600293 | eQTLGen | Blood | Stuart PE et al |
| TNFAIP3 | rs498361 | C | T | 0.891453 | 0.27321 | 0.001102862 | 0.07135045 | eQTLGen | Blood | FinnGen |
| IL23A | rs2695782 | C | T | -1.41415 | 0.278445 | 3.79968E-07 | 0.05053459 | eQTLGen | Blood | FinnGen |
| ASF1A | rs147531464 | T | C | -0.0263717 | 0.0309833 | 0.3946805 | 0.6233908 | eQTLGen | Blood | FinnGen |
| ASF1A | rs147531464 | T | C | -0.0477693 | 0.0563731 | 0.3967848 | 0.5740266 | GTEx | Blood | FinnGen |
| IL12A | rs4680536 | G | A | -0.158383 | 0.219151 | 0.4698577 | 0.5744237 | eQTLGen | Blood | FinnGen |
| RARRES2 | rs11769348 | C | T | -0.469786 | 0.209518 | 0.02494699 | 0.1355429 | eQTLGen | Blood | FinnGen |
| SGSH | rs9894254 | T | C | 0.0216515 | 0.0811613 | 0.7896451 | 0.03132772 | GTEx | Blood | FinnGen |
| PRSS53 | rs7199949 | C | G | -0.406035 | 0.120078 | 0.000721134 | 0.3828808 | GTEx | Blood | FinnGen |
| ICAM5 | rs73510898 | A | G | -0.364979 | 0.0988991 | 0.00022389 | 0.5279828 | GTEx | Blood | FinnGen |
| GCA | rs16846633 | T | C | 0.354363 | 0.157778 | 0.02470711 | 0.1143542 | eQTLGen | Blood | FinnGen |
| IL2RA | rs61839660 | T | C | 0.35751 | 0.135803 | 0.008474195 | 0.8459845 | eQTLGen | Blood | FinnGen |
| DBI | rs12612942 | G | A | 0.10714 | 0.0470039 | 0.0226441 | 0.3500222 | eQTLGen | Blood | FinnGen |
| NFKB1 | rs11097787 | T | C | 0.260113 | 0.0715972 | 0.000280133 | 0.01641456 | eQTLGen | Blood | FinnGen |
| UBLCP1 | rs4921468 | A | G | -0.55681 | 0.130863 | 2.09129E-05 | 0.01299191 | Not_Sun_Exposed_Suprapubic | Skin | VA MVP |
| UBLCP1 | rs11574790 | A | G | -0.285813 | 0.108231 | 0.008271786 | 0.02412686 | Sun_Exposed_Lower_leg | Skin | VA MVP |
| RARRES2 | rs1047207 | G | A | -0.234816 | 0.0636035 | 0.000222618 | 0.4691675 | Not_Sun_Exposed_Suprapubic | Skin | VA MVP |
| RARRES2 | rs57367026 | G | T | -0.23257 | 0.0608413 | 0.000132073 | 0.851569 | Sun_Exposed_Lower_leg | Skin | VA MVP |
| SGSH | rs9904453 | C | T | 0.310147 | 0.0482412 | 1.28363E-10 | 0.08174606 | Not_Sun_Exposed_Suprapubic | Skin | VA MVP |
| PRSS53 | rs59061704 | C | G | 0.231163 | 0.0556445 | 3.26308E-05 | 0.01792051 | Sun_Exposed_Lower_leg | Skin | VA MVP |
| ICAM5 | rs901886 | C | T | 0.1888 | 0.0467421 | 5.36354E-05 | 0.2896143 | Not_Sun_Exposed_Suprapubic | Skin | VA MVP |
| ICAM5 | rs901886 | C | T | 0.154106 | 0.0360081 | 1.87101E-05 | 0.0915376 | Sun_Exposed_Lower_leg | Skin | VA MVP |
| UBLCP1 | rs11574790 | A | G | -0.289658 | 0.146222 | 0.04759765 | 0.1576562 | Sun_Exposed_Lower_leg | Skin | Stuart PE et al |
| RARRES2 | rs1047207 | G | A | -0.221041 | 0.10443 | 0.03429102 | 0.03786972 | Not_Sun_Exposed_Suprapubic | Skin | Stuart PE et al |
| RARRES2 | rs57367026 | G | T | -0.221967 | 0.102145 | 0.02977635 | 0.04002578 | Sun_Exposed_Lower_leg | Skin | Stuart PE et al |
| SGSH | rs9904453 | C | T | 0.244056 | 0.070427 | 0.000529476 | 0.7465572 | Not_Sun_Exposed_Suprapubic | Skin | Stuart PE et al |
| SGSH | rs9904453 | C | T | 0.319783 | 0.0922593 | 0.000527999 | 0.7201896 | Sun_Exposed_Lower_leg | Skin | Stuart PE et al |
| PRSS53 | rs59061704 | C | G | 0.481041 | 0.0925719 | 2.03178E-07 | 0.1446883 | Sun_Exposed_Lower_leg | Skin | Stuart PE et al |
| ICAM5 | rs901886 | C | T | 0.353263 | 0.0757331 | 3.0925E-06 | 0.02770375 | Not_Sun_Exposed_Suprapubic | Skin | Stuart PE et al |
| ICAM5 | rs901886 | C | T | 0.288346 | 0.0571352 | 4.49442E-07 | 0.02406091 | Sun_Exposed_Lower_leg | Skin | Stuart PE et al |
| UBLCP1 | rs4921468 | A | G | -0.258868 | 0.102477 | 0.01153369 | 0.1000021 | Not_Sun_Exposed_Suprapubic | Skin | FinnGen |
| UBLCP1 | rs11574790 | A | G | -0.122182 | 0.13133 | 0.3521918 | 0.4392596 | Sun_Exposed_Lower_leg | Skin | FinnGen |
| RARRES2 | rs1047207 | G | A | -0.135727 | 0.0655327 | 0.03834618 | 0.1568624 | Not_Sun_Exposed_Suprapubic | Skin | FinnGen |
| RARRES2 | rs57367026 | G | T | -0.135595 | 0.0641217 | 0.03445957 | 0.1240037 | Sun_Exposed_Lower_leg | Skin | FinnGen |
| SGSH | rs9904453 | C | T | 0.166907 | 0.0447502 | 0.000191671 | 0.1969254 | Not_Sun_Exposed_Suprapubic | Skin | FinnGen |
| SGSH | rs9904453 | C | T | 0.218695 | 0.0586207 | 0.00019096 | 0.1476663 | Sun_Exposed_Lower_leg | Skin | FinnGen |
| ICAM5 | rs901886 | C | T | 0.136413 | 0.0487456 | 0.005134436 | 0.1193415 | Not_Sun_Exposed_Suprapubic | Skin | FinnGen |
| ICAM5 | rs901886 | C | T | 0.111345 | 0.0387305 | 0.004041828 | 0.154204 | Sun_Exposed_Lower_leg | Skin | FinnGen |

**Table S4. The comparison of index pQTLs in blood with eQTLs in blood and skin tissue.**

| **Target** | **SNP** | **Data** | **EA** | **pQTLs** | **eQTLGen_blood** | **GTEx_Blood** | **Skin_sun_exposed** | **Skin_not_sun_exposed** |
| --- | --- | --- | --- | --- | --- | --- | --- | --- |
|  |  |  |  | **beta** | **beta** | **beta** | **beta** | **beta** |
| COMP | rs12974746 | UKB-PPP | G | -0.605 |  |  | -0.396522 | -0.412216 |
| COMP | rs12974746 | Fenland | A | 0.4862 |  |  | 0.396522 | 0.412216 |
| COMP | rs12974746 | deCODE | G | -0.62 |  |  | -0.396522 | -0.412216 |
| **DDX58** | rs11795343 | UKB-PPP | C | -0.152 | 0.0316379 |  | 0.167258 | 0.130596 |
| **DDX58** | rs7025666 | Fenland | T | 0.3629 |  |  | -0.165145 | -0.195123 |
| FAP | rs10490425 | deCODE | T | 0.89 |  |  | 0.409733 | 0.36343 |
| FAP | rs16846386 | UKB-PPP | A | 1.074 |  |  | 0.237397 | 0.279566 |
| FAP | rs16846387 | Fenland | T | -1.1219 |  |  | -0.389805 | -0.390935 |
| ICAM1 | rs5498 | deCODE | G | -1.2 | -0.070589 | -0.0465617 |  | -0.0661633 |
| ICAM1 | rs5498 | Fenland | A | 1.1941 | 0.070589 | 0.0465617 |  | 0.0661633 |
| ICAM5 | rs281439 | deCODE | G | -0.86 | -0.180218 |  | -0.244966 | -0.214012 |
| ICAM5 | rs281439 | Fenland | C | 0.6908 | 0.180218 |  | 0.244966 | 0.214012 |
| ICAM5 | rs75407602 | UKB-PPP | T | -0.943 | -0.0959934 |  | -0.178356 |  |
| **NFKB1** | rs2272676 | UKB-PPP | T | 0.117 | 0.132075 | 0.0831056 |  | -0.0702051 |
| **NFKB1** | rs230508 | deCODE | A | 0.08 | 0.127329 | 0.0750722 |  | -0.0634238 |
| **NFKB1** | rs62328536 | Fenland | A | 0.1476 | 0.133544 | 0.0824955 |  | -0.0655941 |
| PRSS53 | rs4468641 | UKB-PPP | C | 0.891 | -0.154039 | -0.129899 | 0.2317 | 0.0822901 |
| RARRES2 | rs3735167 | deCODE | T | 0.34 | 0.0658357 |  | 0.232799 | 0.219002 |
| RARRES2 | rs3735167 | UKB-PPP | T | 0.226 | 0.0658357 |  | 0.232799 | 0.219002 |
| RARRES2 | rs3735167 | Fenland | T | 0.3405 | 0.0658357 |  | 0.232799 | 0.219002 |
| **SGSH** | rs7503034 | UKB-PPP | T | -0.9 | 0.0361697 |  | 0.17488 | 0.222391 |

**Table S5. The results of single-cell analysis for DEGs.**

| **Gene** | **cell type** | **logFC** | **p.value** | **Psoriasis _Mean** | **Normal_Mean** | **adjusted_pvalue** | **qval** |
| --- | --- | --- | --- | --- | --- | --- | --- |
| FAP | Fibroblasts | 0.421951067 | 9.60549E-09 | 0.35931208 | 0.267942164 | 2.27039E-08 | 4.34161E-09 |
| RARRES2 | Fibroblasts | 0.846063794 | 3.85464E-81 | 1.405835769 | 0.781623387 | 5.01103E-80 | 9.58247E-81 |
| SGSH | Fibroblasts | -0.003059143 | 0.798870464 | 0.101944582 | 0.102163101 | 0.798870464 | 0.158876807 |
| COMP | Fibroblasts | 0.651522463 | 7.34107E-33 | 1.57414369 | 1.001749314 | 6.36226E-32 | 1.21664E-32 |
| A2ML1 | Keratinocytes 1 | 0.638406256 | 6.48982E-17 | 0.301258929 | 0.193177859 | 2.41051E-16 | 4.60956E-17 |
| DAPK2 | Keratinocytes 1 | -0.445932873 | 0.785049006 | 0.080354782 | 0.10982105 | 0.798870464 | 0.156569965 |
| DBI | Keratinocytes 1 | 0.767877971 | 7.8237E-116 | 2.140081136 | 1.256415538 | 2.0342E-114 | 3.8899E-115 |
| B3GNT2 | Melanocytes | -0.433782663 | 0.000152078 | 0.279859061 | 0.378376157 | 0.000247126 | 4.72574E-05 |
| DBI | Melanocytes | -0.0316375 | 0.028802327 | 2.134635001 | 2.181985577 | 0.037443026 | 0.006975056 |
| B3GNT2 | Vascular endothelial cells | -0.804844014 | 1.14551E-09 | 0.176728225 | 0.309483549 | 2.97832E-09 | 5.69537E-10 |
| GCA | Vascular endothelial cells | -0.546472242 | 2.82699E-05 | 0.120440092 | 0.17636447 | 5.65398E-05 | 1.0812E-05 |
| ICAM1 | Vascular endothelial cells | -0.754095279 | 1.89213E-10 | 0.590809427 | 0.997130158 | 5.46616E-10 | 1.04528E-10 |
| UBLCP1 | Vascular endothelial cells | -0.00746238 | 0.408203497 | 0.152736265 | 0.15353353 | 0.482422314 | 0.088401927 |
| B3GNT2 | Pericytes | -0.29179541 | 6.16288E-05 | 0.285204007 | 0.349360292 | 0.000114453 | 2.17181E-05 |
| RARRES2 | Pericytes | -0.029949161 | 0.792176126 | 1.058642245 | 1.080869456 | 0.798870464 | 0.157755486 |
| UBLCP1 | Pericytes | -0.131475367 | 0.033823967 | 0.140736174 | 0.154259697 | 0.041877293 | 0.008008098 |
| ASF1A | Immune cells | -0.230368829 | 0.001722292 | 0.151043976 | 0.177368083 | 0.002487755 | 0.000475728 |
| B3GNT2 | Immune cells | -0.913866709 | 3.62398E-18 | 0.116384857 | 0.220163455 | 1.88447E-17 | 3.60363E-18 |
| CD8A | Immune cells | -0.118874961 | 0.000677848 | 0.340969667 | 0.370340668 | 0.001036708 | 0.000198247 |
| IL7R | Immune cells | -0.456060583 | 2.46675E-17 | 1.06718073 | 1.464320597 | 1.06892E-16 | 2.04408E-17 |
| NFKB1 | Immune cells | -0.524302785 | 2.07157E-14 | 0.557839346 | 0.802744179 | 6.73259E-14 | 1.28746E-14 |
| TNFAIP3 | Immune cells | -0.561178582 | 1.39974E-29 | 1.064173068 | 1.570635258 | 9.09828E-29 | 1.73984E-29 |
| UBLCP1 | Immune cells | -0.384859673 | 8.84489E-05 | 0.112565638 | 0.147286376 | 0.000153311 | 2.93174E-05 |
| IL2RA | Immune cells | 0.711869635 | 0.023244118 | 0.20637803 | 0.125610182 | 0.031807741 | 0.005775138 |
| A2ML1 | Keratinocytes 2 | -0.258546373 | 0.553774905 | 0.193825299 | 0.232064198 | 0.626006415 | 0.1160775 |
| TNFAIP3 | Keratinocytes 2 | -0.643283994 | 1.95699E-05 | 0.813991205 | 1.271918798 | 4.24015E-05 | 7.85673E-06 |

**Table S6. The results of colocalization for the identified targets.**

| **Targets** | **PP.H0.abf** | **PP.H1.abf** | **PP.H2.abf** | **PP.H3.abf** | **PP.H4.abf** | **Shared variant** | **data** |
| --- | --- | --- | --- | --- | --- | --- | --- |
| A2ML1 | 4.09897E-83 | 4.48828E-84 | 0.508806934 | 0.055277312 | 0.435915754 | rs1558526 | Fenland |
| ASF1A | 4.6936E-55 | 7.13051E-56 | 0.154702701 | 0.022679812 | 0.822617488 | rs147886588 | Fenland |
| B3GNT2 | 2.8365E-52 | 7.20732E-50 | 0.003876204 | 0.984899783 | 0.011224013 | rs112392179 | Fenland |
| CD8A | 6.25547E-94 | 6.14443E-95 | 0.265770895 | 0.025396506 | 0.708832599 | rs3020726 | Fenland |
| COMP | 2.56985E-23 | 1.25729E-22 | 0.13408011 | 0.655773054 | 0.210146837 | rs12974746 | Fenland |
| CRTAM | 2.42378E-69 | 7.39278E-70 | 0.701863958 | 0.213991996 | 0.084144046 | rs2370794 | Fenland |
| DAPK2 | 1.0544E-143 | 2.4103E-144 | 0.717299567 | 0.163844879 | 0.118855554 | rs4436737 | Fenland |
| DBI | 1.3075E-118 | 3.8219E-119 | 0.479809583 | 0.139870774 | 0.380319644 | rs6733335 | Fenland |
| DDX58 | 1.677E-132 | 3.6259E-125 | 4.6236E-08 | 0.99965503 | 0.000344924 | rs7045087 | Fenland |
| FAP | 1.45567E-58 | 1.3178E-49 | 1.10462E-09 | 0.999999889 | 1.10332E-07 | rs6746339 | Fenland |
| GCA | 9.1198E-206 | 8.2561E-197 | 1.10462E-09 | 0.999999958 | 4.09654E-08 | rs17783344 | Fenland |
| ICAM5 | 0 | 0 | 1.35151E-21 | 1 | 1.68392E-18 | rs281439 | Fenland |
| IL12B | 0 | 7.7194E-278 | 1.32212E-73 | 1 | 7.03988E-62 | rs4244437 | Fenland |
| IL12B | 6.115E-263 | 4.6252E-190 | 1.32212E-73 | 1 | 7.10831E-62 | rs4244437 | Fenland |
| IL7R | 1.4351E-29 | 3.99235E-28 | 0.034690487 | 0.965066877 | 0.000242635 | rs6451229 | Fenland |
| MMP12 | 9.2081E-281 | 3.3074E-280 | 0.020384917 | 0.072312119 | 0.907302964 | rs17368814 | Fenland |
| NFKB1 | 5.49238E-26 | 3.05163E-23 | 0.001779208 | 0.988538806 | 0.009681986 | rs62328536 | Fenland |
| RARRES2 | 7.9813E-110 | 2.3104E-109 | 0.023470091 | 0.067031282 | 0.909498627 | rs3735167 | Fenland |
| TIMD4 | 2.08201E-49 | 5.56482E-49 | 0.261192955 | 0.698079596 | 0.040727449 | rs12657266 | Fenland |
| TNFAIP3 | 1.38079E-78 | 1.68094E-69 | 8.21427E-10 | 0.999986607 | 1.33924E-05 | rs59693083 | Fenland |
| UBLCP1 | 7.88798E-86 | 5.96616E-13 | 1.32212E-73 | 1 | 8.28976E-13 | rs918519 | Fenland |
| XCL2 | 1.65317E-08 | 1.98945E-08 | 0.048115571 | 0.057008424 | 0.894875968 | rs10753774 | Fenland |
| CD8A | 0 | 0 | 0.221003304 | 0.053502213 | 0.725494483 | rs3020726 | UKB-PPP |
| COMP | 2.6714E-209 | 1.3452E-208 | 0.118878561 | 0.598413026 | 0.282708413 | rs12974746 | UKB-PPP |
| CRTAM | 0 | 0 | 0.57087774 | 0.320856137 | 0.108266123 | rs2370794 | UKB-PPP |
| CTF1 | 8.97774E-14 | 6.31538E-09 | 6.36308E-07 | 0.043978906 | 0.956020451 | rs2305880 | UKB-PPP |
| DAPK2 | 0 | 0 | 0.561169186 | 0.31114161 | 0.127689204 | rs55986634 | UKB-PPP |
| DBI | 0 | 0 | 0.372435659 | 0.205555391 | 0.42200895 | rs72833218 | UKB-PPP |
| DDX58 | 0 | 0 | 4.65692E-08 | 0.985602784 | 0.014397169 | rs7045087 | UKB-PPP |
| FAP | 8.2678E-232 | 7.4848E-223 | 1.10462E-09 | 0.999999776 | 2.23335E-07 | rs16846386 | UKB-PPP |
| ICAM1 | 0 | 0 | 1.35151E-21 | 1 | 6.63112E-20 | rs5030377 | UKB-PPP |
| ICAM5 | 0 | 0 | 1.35151E-21 | 1 | 2.61041E-18 | rs281439 | UKB-PPP |
| IL12B | 0 | 0 | 1.32212E-73 | 1 | 1.23374E-61 | rs4244437 | UKB-PPP |
| IL2RA | 0 | 0 | 2.45612E-10 | 0.999999896 | 1.03738E-07 | rs12722497 | UKB-PPP |
| IL7R | 0 | 0 | 0.004279448 | 0.116740454 | 0.878980098 | rs11742270 | UKB-PPP |
| LBP | 0 | 0 | 0.498568881 | 0.361022651 | 0.140408468 | rs2232613 | UKB-PPP |
| MMP12 | 0 | 0 | 0.014370055 | 0.056812343 | 0.928817602 | rs2276109 | UKB-PPP |
| NFKB1 | 3.13755E-60 | 1.67217E-57 | 0.001829461 | 0.975003912 | 0.023166628 | rs2272676 | UKB-PPP |
| PRSS53 | 0 | 0 | 1.36075E-05 | 0.957180651 | 0.042805742 | rs7199949 | UKB-PPP |
| RARRES2 | 3.5627E-197 | 1.0783E-196 | 0.018938696 | 0.056753309 | 0.924307995 | rs3735167 | UKB-PPP |
| SGSH | 0 | 0 | 2.90878E-09 | 0.9999632 | 3.67974E-05 | rs7503034 | UKB-PPP |
| TIMD4 | 5.1656E-256 | 1.606E-255 | 0.230836479 | 0.717625557 | 0.051537964 | rs12657266 | UKB-PPP |
| A2ML1 | 0 | 0 | 0.466776179 | 0.117838819 | 0.415385003 | rs1558526 | deCODE |
| ASF1A | 1.5555E-108 | 5.8119E-109 | 0.160405481 | 0.059153815 | 0.780440704 | rs3951094 | deCODE |
| B3GNT2 | 2.5008E-133 | 6.358E-131 | 0.00379149 | 0.963900649 | 0.032307861 | rs4073090 | deCODE |
| CD8A | 0 | 0 | 0.259233914 | 0.048447341 | 0.692318745 | rs3020726 | deCODE |
| COMP | 6.8626E-173 | 3.3823E-172 | 0.13343402 | 0.657429253 | 0.209136727 | rs12974746 | deCODE |
| CRTAM | 1.1808E-171 | 5.4809E-172 | 0.627658211 | 0.291269836 | 0.081071953 | rs2370794 | deCODE |
| CTF1 | 1.29737E-15 | 9.36238E-11 | 1.09587E-06 | 0.078160262 | 0.921838642 | rs12934900 | deCODE |
| DAPK2 | 0 | 0 | 0.629095135 | 0.27757036 | 0.093334505 | rs55986634 | deCODE |
| DBI | 2.6109E-211 | 1.1799E-211 | 0.44583663 | 0.201136165 | 0.353027206 | rs72833218 | deCODE |
| DDX58 | 1.1318E-261 | 2.2738E-254 | 4.92693E-08 | 0.989810189 | 0.010189761 | rs1133071 | deCODE |
| FAP | 6.7302E-156 | 6.0927E-147 | 1.10462E-09 | 0.99999985 | 1.49254E-07 | rs16846600 | deCODE |
| GCA | 0 | 0 | 1.10462E-09 | 0.999999958 | 4.09654E-08 | rs17783344 | deCODE |
| ICAM1 | 0 | 0 | 1.35151E-21 | 1 | 1.73259E-19 | rs5498 | deCODE |
| ICAM5 | 0 | 0 | 1.35151E-21 | 1 | 1.82065E-18 | rs281439 | deCODE |
| IL12B | 0 | 0 | 1.32212E-73 | 1 | 7.29041E-62 | rs4244437 | deCODE |
| IL2RA | 1.5875E-49 | 6.46347E-40 | 2.45612E-10 | 0.999999928 | 7.17005E-08 | rs12722497 | deCODE |
| IL7R | 4.72114E-60 | 1.29164E-58 | 0.035253694 | 0.964496829 | 0.000249476 | rs6451229 | deCODE |
| LBP | 0 | 0 | 0.711124196 | 0.14805951 | 0.140816295 | rs2232613 | deCODE |
| MMP12 | 0 | 0 | 0.020965738 | 0.081162881 | 0.897871381 | rs201707806 | deCODE |
| NFKB1 | 9.33435E-22 | 4.97322E-19 | 0.001834969 | 0.977626773 | 0.020538258 | rs230501 | deCODE |
| RARRES2 | 0 | 0 | 0.017016796 | 0.048793236 | 0.934189968 | rs3735167 | deCODE |
| TIMD4 | 2.6549E-212 | 7.8876E-212 | 0.242379578 | 0.720075612 | 0.03754481 | rs4704826 | deCODE |
| TNFAIP3 | 2.79648E-81 | 3.40436E-72 | 8.21178E-10 | 0.999681618 | 0.000318381 | rs2230926 | deCODE |
| UBLCP1 | 4.0532E-192 | 3.0657E-119 | 1.32212E-73 | 1 | 4.31431E-70 | rs13158921 | deCODE |
| XCL2 | 7.55697E-31 | 1.0631E-30 | 0.05905587 | 0.082220321 | 0.858723809 | rs61801329 | deCODE |
| DAPK2 | 7.98265E-90 | 2.30795E-90 | 0.760878872 | 0.219967134 | 0.019153994 | rs332280 | eQTLGen |
| ICAM1 | 1.42237E-85 | 1.12989E-67 | 1.25885E-18 | 1 | 1.26404E-20 | rs281438 | eQTLGen |
| ICAM5 | 3.9385E-76 | 3.12865E-58 | 1.25885E-18 | 1 | 1.31978E-15 | rs281440 | eQTLGen |
| RARRES2 | 1.83866E-10 | 4.9558E-10 | 0.023784962 | 0.063195405 | 0.913019632 | rs11769348 | eQTLGen |
| DDX58 | 2.6737E-142 | 5.382E-135 | 4.96784E-08 | 0.99999995 | 3.33628E-10 | rs7022554 | eQTLGen |
| NFKB1 | 5.2895E-106 | 2.3605E-103 | 0.00222188 | 0.991550189 | 0.006227931 | rs10013613 | eQTLGen |
| CRTAM | 3.9918E-223 | 1.221E-223 | 0.760122895 | 0.232490835 | 0.00738627 | rs10444340 | eQTLGen |
| IL23A | 1.75672E-24 | 9.18814E-12 | 8.80376E-15 | 0.045091132 | 0.954908868 | rs2695782 | eQTLGen |
| ASF1A | 0 | 0 | 0.192491573 | 0.036013958 | 0.771494469 | rs147531464 | eQTLGen |
| GCA | 1.31715E-23 | 1.1924E-14 | 1.10462E-09 | 0.999999936 | 6.32808E-08 | rs16846633 | eQTLGen |
| TNFAIP3 | 1.42428E-15 | 1.73387E-06 | 8.21292E-10 | 0.999813725 | 0.000184541 | rs583522 | eQTLGen |
| IL2RA | 2.2292E-61 | 8.88531E-52 | 3.91524E-13 | 0.000561127 | 0.999438873 | rs61839660 | eQTLGen |
| XCL2 | 0 | 0 | 0.472970742 | 0.52288847 | 0.004140788 | rs11580138 | eQTLGen |
| TIMD4 | 2.85648E-55 | 7.44777E-55 | 0.049289341 | 0.127690183 | 0.823020476 | rs953569 | eQTLGen |
| PRSS53 | 1.6363E-100 | 1.15373E-95 | 1.41824E-05 | 0.999985682 | 1.36023E-07 | rs17708472 | eQTLGen |
| CD8A | 8.4825E-98 | 8.7905E-99 | 0.869602699 | 0.090077624 | 0.040319676 | rs1515950 | eQTLGen |
| DBI | 1.4266E-134 | 3.9816E-135 | 0.241261159 | 0.066642842 | 0.692095999 | rs12612942 | eQTLGen |
| UBLCP1 | 3.3227E-131 | 2.1023E-58 | 1.58053E-73 | 1 | 2.17316E-26 | rs4921213 | eQTLGen |
| A2ML1 | 0.14722838 | 0.01940354 | 0.726937023 | 0.095793931 | 0.010637125 | rs7306169 | eQTLGen |
| IL7R | 9.6057E-261 | 2.5536E-259 | 0.031447064 | 0.835866089 | 0.132686847 | rs10058453 | eQTLGen |
| IL12A | 5.45973E-09 | 2.54465E-09 | 0.67949128 | 0.316690792 | 0.00381792 | rs4680536 | eQTLGen |
| B3GNT2 | 0 | 0 | 0.004654955 | 0.993680456 | 0.001664589 | rs11888817 | eQTLGen |
| SGSH | 9.1736E-111 | 2.8764E-102 | 3.18924E-09 | 0.999999997 | 1.36399E-10 | rs114747872 | eQTLGen |
| DAPK2 | 4.35329E-07 | 8.84557E-08 | 0.814856781 | 0.165553383 | 0.019589312 | rs12912128 | GTEx(blood) |
| ICAM1 | 5.50138E-25 | 0.000407053 | 1.35092E-21 | 0.999559641 | 3.33058E-05 | rs34536443 | GTEx(blood) |
| ICAM5 | 1.0653E-22 | 0.078822609 | 1.23819E-21 | 0.91614271 | 0.005034681 | rs34536443 | GTEx(blood) |
| COMP | 0.1520093 | 0.664080943 | 0.028564507 | 0.124758782 | 0.030586469 | rs7257932 | GTEx(blood) |
| RARRES2 | 0.204406558 | 0.540045893 | 0.063205235 | 0.166964018 | 0.025378297 | rs883138 | GTEx(blood) |
| DDX58 | 3.02493E-08 | 0.571453777 | 2.10717E-08 | 0.398045694 | 0.030500479 | rs72712903 | GTEx(blood) |
| NFKB1 | 1.87132E-05 | 0.009224128 | 0.001931366 | 0.951972922 | 0.03685287 | rs1120986 | GTEx(blood) |
| CRTAM | 0.579639872 | 0.157107356 | 0.196036944 | 0.053120355 | 0.014095472 | rs7930461 | GTEx(blood) |
| IL23A | 1.29682E-13 | 0.652635277 | 1.9635E-14 | 0.098565959 | 0.248798764 | rs773664 | GTEx(blood) |
| ASF1A | 2.65143E-11 | 3.31693E-12 | 0.176696192 | 0.021302637 | 0.802001171 | rs147886588 | GTEx(blood) |
| GCA | 8.16046E-10 | 0.738756567 | 2.14444E-10 | 0.194066048 | 0.067177384 | rs2111485 | GTEx(blood) |
| TNFAIP3 | 6.27531E-10 | 0.7639355 | 1.69504E-10 | 0.206318583 | 0.029745916 | rs583522 | GTEx(blood) |
| LBP | 0.659047203 | 0.052063747 | 0.262734739 | 0.020750249 | 0.005404063 | rs111735129 | GTEx(blood) |
| IL2RA | 1.77438E-10 | 0.707247843 | 5.73601E-11 | 0.228565931 | 0.064186226 | rs61839660 | GTEx(blood) |
| XCL2 | 9.83393E-35 | 1.05186E-34 | 0.481145033 | 0.514641082 | 0.004213885 | rs61801331 | GTEx(blood) |
| TIMD4 | 0.180335431 | 0.446067039 | 0.097955691 | 0.242264016 | 0.033377823 | rs7705741 | GTEx(blood) |
| CTF1 | 1.34099E-05 | 0.874576846 | 1.37095E-06 | 0.089376017 | 0.036032356 | rs9938550 | GTEx(blood) |
| PRSS53 | 9.87823E-09 | 0.000644247 | 1.19545E-05 | 0.779437356 | 0.219906433 | rs7184567 | GTEx(blood) |
| CD8A | 0.532879899 | 0.046477126 | 0.374839824 | 0.032679943 | 0.013123208 | rs2367376 | GTEx(blood) |
| DBI | 0.618126696 | 0.148626627 | 0.1723735 | 0.041427222 | 0.019445955 | rs12612942 | GTEx(blood) |
| UBLCP1 | 8.48454E-74 | 0.640128744 | 4.39302E-74 | 0.331409411 | 0.028461845 | rs918519 | GTEx(blood) |
| IL7R | 0.03527989 | 0.766121716 | 0.007310362 | 0.158715863 | 0.032572169 | rs12516866 | GTEx(blood) |
| IL12A | 0.56595912 | 0.248808378 | 0.114415608 | 0.050279141 | 0.020537754 | rs9879379 | GTEx(blood) |
| B3GNT2 | 2.53284E-63 | 5.40571E-61 | 0.004655914 | 0.993685008 | 0.001659078 | rs11888817 | GTEx(blood) |
| SGSH | 2.29231E-11 | 0.006804248 | 2.09337E-09 | 0.621003597 | 0.372192153 | rs9914372 | GTEx(blood) |
| DAPK2 | 0.000352308 | 7.09151E-05 | 0.81607167 | 0.164245846 | 0.019259262 | rs332277 | GTEx(Skin_Sun_Exposured) |
| FAP | 1.46255E-10 | 0.132402967 | 9.50746E-10 | 0.860691711 | 0.00690532 | rs2111485 | GTEx(Skin_Sun_Exposured) |
| ICAM1 | 1.06266E-21 | 0.786275635 | 2.19939E-22 | 0.162684013 | 0.051040352 | rs34536443 | GTEx(Skin_Sun_Exposured) |
| ICAM5 | 1.33935E-30 | 9.91003E-10 | 1.35151E-21 | 0.999999999 | 2.33511E-10 | rs34536443 | GTEx(Skin_Sun_Exposured) |
| COMP | 0.072305472 | 0.315905679 | 0.110847472 | 0.484280651 | 0.016660726 | rs7257932 | GTEx(Skin_Sun_Exposured) |
| RARRES2 | 1.49758E-15 | 3.95222E-15 | 0.016820614 | 0.043451104 | 0.939728282 | rs57367026 | GTEx(Skin_Sun_Exposured) |
| DDX58 | 4.96832E-21 | 9.38591E-14 | 5.29339E-08 | 0.99999991 | 3.68402E-08 | rs6476363 | GTEx(Skin_Sun_Exposured) |
| NFKB1 | 0.001556528 | 0.767244613 | 0.00036255 | 0.178656331 | 0.052179977 | rs228614 | GTEx(Skin_Sun_Exposured) |
| CRTAM | 0.552391261 | 0.149048743 | 0.22565972 | 0.060876517 | 0.012023758 | rs11218864 | GTEx(Skin_Sun_Exposured) |
| IL23A | 3.33055E-14 | 0.167612614 | 2.1595E-14 | 0.107953894 | 0.724433492 | rs773664 | GTEx(Skin_Sun_Exposured) |
| ASF1A | 0.53837272 | 0.066684412 | 0.342806582 | 0.042451336 | 0.00968495 | rs147886588 | GTEx(Skin_Sun_Exposured) |
| GCA | 7.08572E-10 | 0.641461396 | 2.94266E-10 | 0.266303634 | 0.092234968 | rs2111485 | GTEx(Skin_Sun_Exposured) |
| TNFAIP3 | 6.19129E-10 | 0.753708172 | 1.77712E-10 | 0.216311287 | 0.029980541 | rs583522 | GTEx(Skin_Sun_Exposured) |
| LBP | 0.735265838 | 0.057353033 | 0.187407307 | 0.014612995 | 0.005360828 | rs111735129 | GTEx(Skin_Sun_Exposured) |
| IL2RA | 1.802E-10 | 0.718255506 | 5.72436E-11 | 0.228112123 | 0.053632371 | rs61839660 | GTEx(Skin_Sun_Exposured) |
| TIMD4 | 0.215925608 | 0.53408182 | 0.056569065 | 0.139867362 | 0.053556144 | rs7705706 | GTEx(Skin_Sun_Exposured) |
| CTF1 | 1.17887E-05 | 0.768845687 | 1.82038E-06 | 0.118610847 | 0.112529857 | rs8050588 | GTEx(Skin_Sun_Exposured) |
| PRSS53 | 2.19563E-13 | 1.43197E-08 | 1.11952E-05 | 0.729870976 | 0.270117815 | rs7184567 | GTEx(Skin_Sun_Exposured) |
| CD8A | 0.799610675 | 0.071916265 | 0.111911576 | 0.010058724 | 0.00650276 | rs150292736 | GTEx(Skin_Sun_Exposured) |
| DBI | 0.628276506 | 0.150823506 | 0.170091719 | 0.040822083 | 0.009986186 | rs62159782 | GTEx(Skin_Sun_Exposured) |
| UBLCP1 | 1.1245E-76 | 0.000848396 | 1.32421E-73 | 0.999069301 | 8.23031E-05 | rs918519 | GTEx(Skin_Sun_Exposured) |
| IL7R | 0.031957581 | 0.693892736 | 0.008966301 | 0.194614105 | 0.070569276 | rs12516866 | GTEx(Skin_Sun_Exposured) |
| IL12A | 0.403250155 | 0.177150737 | 0.278298882 | 0.12223967 | 0.019060556 | rs9879379 | GTEx(Skin_Sun_Exposured) |
| B3GNT2 | 0.003375275 | 0.720360156 | 0.001153363 | 0.246124816 | 0.02898639 | rs11679753 | GTEx(Skin_Sun_Exposured) |
| SGSH | 9.71236E-28 | 2.88292E-19 | 5.69024E-11 | 0.015906242 | 0.984093758 | rs9914372 | GTEx(Skin_Sun_Exposured) |
| DAPK2 | 0.635580886 | 0.127908507 | 0.186848215 | 0.0375905 | 0.012071891 | rs78964183 | GTEx(Skin_Not_Sun_Exposured) |
| FAP | 4.39055E-10 | 0.397471105 | 6.01457E-10 | 0.544433828 | 0.058095065 | rs2111485 | GTEx(Skin_Not_Sun_Exposured) |
| ICAM1 | 1.00674E-21 | 0.744900611 | 2.68985E-22 | 0.198968545 | 0.056130845 | rs34536443 | GTEx(Skin_Not_Sun_Exposured) |
| ICAM5 | 3.21465E-26 | 2.37855E-05 | 1.35148E-21 | 0.999974266 | 1.94893E-06 | rs34536443 | GTEx(Skin_Not_Sun_Exposured) |
| COMP | 0.138963963 | 0.60704054 | 0.042050389 | 0.183661721 | 0.028283388 | rs7257932 | GTEx(Skin_Not_Sun_Exposured) |
| RARRES2 | 1.71958E-09 | 4.5382E-09 | 0.016753196 | 0.04327382 | 0.939972977 | rs1047207 | GTEx(Skin_Not_Sun_Exposured) |
| DDX58 | 1.31545E-16 | 2.48508E-09 | 5.29339E-08 | 0.99999993 | 1.4538E-08 | rs10813829 | GTEx(Skin_Not_Sun_Exposured) |
| NFKB1 | 0.001028864 | 0.507152601 | 0.000950867 | 0.468683758 | 0.022183911 | rs228614 | GTEx(Skin_Not_Sun_Exposured) |
| CRTAM | 0.606493114 | 0.163192345 | 0.172225375 | 0.046329843 | 0.011759322 | rs7930461 | GTEx(Skin_Not_Sun_Exposured) |
| IL23A | 1.52454E-14 | 0.076723638 | 1.5646E-14 | 0.077894218 | 0.845382143 | rs2643623 | GTEx(Skin_Not_Sun_Exposured) |
| ASF1A | 0.496068703 | 0.061045987 | 0.382184798 | 0.047017803 | 0.013682709 | rs62422262 | GTEx(Skin_Not_Sun_Exposured) |
| GCA | 8.85089E-10 | 0.801260388 | 1.80154E-10 | 0.163055406 | 0.035684205 | rs2111485 | GTEx(Skin_Not_Sun_Exposured) |
| TNFAIP3 | 5.96538E-10 | 0.726206232 | 1.89426E-10 | 0.230557613 | 0.043236155 | rs583522 | GTEx(Skin_Not_Sun_Exposured) |
| LBP | 0.773901307 | 0.059993616 | 0.149438233 | 0.011579516 | 0.005087328 | rs111735129 | GTEx(Skin_Not_Sun_Exposured) |
| IL2RA | 1.5787E-10 | 0.629250995 | 6.36959E-11 | 0.253766999 | 0.116982005 | rs61839660 | GTEx(Skin_Not_Sun_Exposured) |
| TIMD4 | 0.223563064 | 0.553300059 | 0.053758863 | 0.133012366 | 0.036365647 | rs7705741 | GTEx(Skin_Not_Sun_Exposured) |
| CTF1 | 1.34442E-05 | 0.876814304 | 1.19824E-06 | 0.078102679 | 0.045068375 | rs11076 | GTEx(Skin_Not_Sun_Exposured) |
| PRSS53 | 1.03529E-05 | 0.675204412 | 2.42732E-06 | 0.158139452 | 0.166643356 | rs7184567 | GTEx(Skin_Not_Sun_Exposured) |
| CD8A | 0.780194915 | 0.069486343 | 0.132343966 | 0.01178073 | 0.006194046 | rs150292736 | GTEx(Skin_Not_Sun_Exposured) |
| DBI | 0.622877641 | 0.149585479 | 0.175405567 | 0.042114028 | 0.010017285 | rs62159782 | GTEx(Skin_Not_Sun_Exposured) |
| UBLCP1 | 3.94123E-75 | 0.029735165 | 1.28408E-73 | 0.968791916 | 0.00147292 | rs918519 | GTEx(Skin_Not_Sun_Exposured) |
| IL7R | 0.027673254 | 0.60083374 | 0.008650365 | 0.187639028 | 0.175203612 | rs12516866 | GTEx(Skin_Not_Sun_Exposured) |
| IL12A | 0.560112255 | 0.245620908 | 0.120680147 | 0.052900075 | 0.020686615 | rs9879379 | GTEx(Skin_Not_Sun_Exposured) |
| B3GNT2 | 0.003586796 | 0.765505041 | 0.000926428 | 0.197688911 | 0.032292824 | rs11679753 | GTEx(Skin_Not_Sun_Exposured) |
| SGSH | 3.3651E-27 | 9.98863E-19 | 5.58312E-11 | 0.01558795 | 0.98441205 | rs9914372 | GTEx(Skin_Not_Sun_Exposured) |

**Table S7. The GO terms of enrichment analysis.**

| **ONTOLOGY** | **ID** | **Description** | **GeneRatio** | **p.adjust** | **qvalue** | **geneID** |
| --- | --- | --- | --- | --- | --- | --- |
| BP | GO:0050691 | regulation of defense response to virus by host | 5/29 | 3.34131E-06 | 1.59303E-06 | IL12B/MMP12/TNFAIP3/IL23A/RIGI |
| BP | GO:0001910 | regulation of leukocyte mediated cytotoxicity | 6/29 | 3.34131E-06 | 1.59303E-06 | CRTAM/ICAM1/IL12A/IL12B/IL7R/IL23A |
| BP | GO:0031341 | regulation of cell killing | 6/29 | 4.98949E-06 | 2.37883E-06 | CRTAM/ICAM1/IL12A/IL12B/IL7R/IL23A |
| BP | GO:0050688 | regulation of defense response to virus | 5/29 | 1.04641E-05 | 4.98893E-06 | IL12B/MMP12/TNFAIP3/IL23A/RIGI |
| BP | GO:0002456 | T cell mediated immunity | 6/29 | 1.04641E-05 | 4.98893E-06 | CD8A/ICAM1/IL12A/IL12B/IL7R/IL23A |
| BP | GO:0001909 | leukocyte mediated cytotoxicity | 6/29 | 1.16276E-05 | 5.54365E-06 | CRTAM/ICAM1/IL12A/IL12B/IL7R/IL23A |
| BP | GO:0002703 | regulation of leukocyte mediated immunity | 7/29 | 1.16276E-05 | 5.54365E-06 | CRTAM/ICAM1/IL12A/IL12B/IL7R/IL23A/RIGI |
| BP | GO:0002687 | positive regulation of leukocyte migration | 6/29 | 1.16276E-05 | 5.54365E-06 | DAPK2/ICAM1/IL12A/LBP/RARRES2/IL23A |
| BP | GO:0050900 | leukocyte migration | 8/29 | 1.20896E-05 | 5.76393E-06 | CRTAM/DAPK2/ICAM1/IL12A/LBP/RARRES2/XCL2/IL23A |
| BP | GO:0002697 | regulation of immune effector process | 8/29 | 1.21821E-05 | 5.80801E-06 | CRTAM/ICAM1/IL12A/IL12B/IL7R/LBP/IL23A/RIGI |
| BP | GO:0002690 | positive regulation of leukocyte chemotaxis | 5/29 | 2.64672E-05 | 1.26187E-05 | DAPK2/IL12A/LBP/RARRES2/IL23A |
| BP | GO:0002420 | natural killer cell mediated cytotoxicity directed against tumor cell target | 3/29 | 2.80711E-05 | 1.33834E-05 | CRTAM/IL12A/IL12B |
| BP | GO:0002855 | regulation of natural killer cell mediated immune response to tumor cell | 3/29 | 2.80711E-05 | 1.33834E-05 | CRTAM/IL12A/IL12B |
| BP | GO:0002443 | leukocyte mediated immunity | 8/29 | 3.12859E-05 | 1.49161E-05 | CD8A/CRTAM/ICAM1/IL12A/IL12B/IL7R/IL23A/RIGI |
| BP | GO:0002423 | natural killer cell mediated immune response to tumor cell | 3/29 | 3.3417E-05 | 1.59322E-05 | CRTAM/IL12A/IL12B |
| BP | GO:0046006 | regulation of activated T cell proliferation | 4/29 | 3.41135E-05 | 1.62642E-05 | CRTAM/IL12B/IL2RA/IL23A |
| BP | GO:0070661 | leukocyte proliferation | 7/29 | 3.41135E-05 | 1.62642E-05 | CRTAM/IL12A/IL12B/IL7R/TNFAIP3/IL2RA/IL23A |
| BP | GO:0042742 | defense response to bacterium | 7/29 | 3.41135E-05 | 1.62642E-05 | IL12A/IL12B/IL7R/LBP/NFKB1/RARRES2/IL23A |
| BP | GO:0051133 | regulation of NK T cell activation | 3/29 | 3.51397E-05 | 1.67535E-05 | IL12A/IL12B/IL23A |
| BP | GO:0050798 | activated T cell proliferation | 4/29 | 3.57559E-05 | 1.70472E-05 | CRTAM/IL12B/IL2RA/IL23A |
| BP | GO:0001914 | regulation of T cell mediated cytotoxicity | 4/29 | 4.59332E-05 | 2.18994E-05 | IL12A/IL12B/IL7R/IL23A |
| BP | GO:0046634 | regulation of alpha-beta T cell activation | 5/29 | 4.59332E-05 | 2.18994E-05 | CRTAM/IL12A/IL12B/IL2RA/IL23A |
| BP | GO:0071622 | regulation of granulocyte chemotaxis | 4/29 | 4.59332E-05 | 2.18994E-05 | DAPK2/LBP/RARRES2/IL23A |
| BP | GO:0032725 | positive regulation of granulocyte macrophage colony-stimulating factor production | 3/29 | 4.59332E-05 | 2.18994E-05 | IL12B/IL23A/RIGI |
| BP | GO:0002688 | regulation of leukocyte chemotaxis | 5/29 | 4.67696E-05 | 2.22982E-05 | DAPK2/IL12A/LBP/RARRES2/IL23A |
| BP | GO:0002685 | regulation of leukocyte migration | 6/29 | 4.67696E-05 | 2.22982E-05 | DAPK2/ICAM1/IL12A/LBP/RARRES2/IL23A |
| BP | GO:0071621 | granulocyte chemotaxis | 5/29 | 4.67696E-05 | 2.22982E-05 | DAPK2/LBP/RARRES2/XCL2/IL23A |
| BP | GO:0030595 | leukocyte chemotaxis | 6/29 | 4.67696E-05 | 2.22982E-05 | DAPK2/IL12A/LBP/RARRES2/XCL2/IL23A |
| BP | GO:0002449 | lymphocyte mediated immunity | 7/29 | 4.67696E-05 | 2.22982E-05 | CD8A/CRTAM/ICAM1/IL12A/IL12B/IL7R/IL23A |
| BP | GO:0045089 | positive regulation of innate immune response | 7/29 | 4.67696E-05 | 2.22982E-05 | CRTAM/IL12A/IL12B/LBP/MMP12/TNFAIP3/RIGI |
| BP | GO:0007259 | cell surface receptor signaling pathway via JAK-STAT | 5/29 | 4.82167E-05 | 2.29881E-05 | CTF1/IL12A/IL12B/IL7R/IL23A |
| BP | GO:0002460 | adaptive immune response based on somatic recombination of immune receptors built from immunoglobulin superfamily domains | 7/29 | 5.46206E-05 | 2.60413E-05 | CD8A/ICAM1/IL12A/IL12B/IL7R/TNFAIP3/IL23A |
| BP | GO:0032604 | granulocyte macrophage colony-stimulating factor production | 3/29 | 5.89034E-05 | 2.80832E-05 | IL12B/IL23A/RIGI |
| BP | GO:0032645 | regulation of granulocyte macrophage colony-stimulating factor production | 3/29 | 5.89034E-05 | 2.80832E-05 | IL12B/IL23A/RIGI |
| BP | GO:0001913 | T cell mediated cytotoxicity | 4/29 | 5.89034E-05 | 2.80832E-05 | IL12A/IL12B/IL7R/IL23A |
| BP | GO:0002833 | positive regulation of response to biotic stimulus | 7/29 | 6.15293E-05 | 2.93352E-05 | CRTAM/IL12A/IL12B/LBP/MMP12/TNFAIP3/RIGI |
| BP | GO:0001906 | cell killing | 6/29 | 6.15293E-05 | 2.93352E-05 | CRTAM/ICAM1/IL12A/IL12B/IL7R/IL23A |
| BP | GO:0050921 | positive regulation of chemotaxis | 5/29 | 6.15293E-05 | 2.93352E-05 | DAPK2/IL12A/LBP/RARRES2/IL23A |
| BP | GO:0002836 | positive regulation of response to tumor cell | 3/29 | 6.15293E-05 | 2.93352E-05 | CRTAM/IL12A/IL12B |
| BP | GO:0002839 | positive regulation of immune response to tumor cell | 3/29 | 6.15293E-05 | 2.93352E-05 | CRTAM/IL12A/IL12B |
| BP | GO:0009615 | response to virus | 7/29 | 6.3713E-05 | 3.03763E-05 | IL12A/IL12B/MMP12/NFKB1/TNFAIP3/IL23A/RIGI |
| BP | GO:0070663 | regulation of leukocyte proliferation | 6/29 | 6.41411E-05 | 3.05804E-05 | CRTAM/IL12A/IL12B/TNFAIP3/IL2RA/IL23A |
| BP | GO:0002699 | positive regulation of immune effector process | 6/29 | 6.41411E-05 | 3.05804E-05 | CRTAM/IL12A/IL12B/LBP/IL23A/RIGI |
| BP | GO:0007159 | leukocyte cell-cell adhesion | 7/29 | 6.41411E-05 | 3.05804E-05 | CRTAM/ICAM1/IL12A/IL12B/IL7R/IL2RA/IL23A |
| BP | GO:0051132 | NK T cell activation | 3/29 | 6.48809E-05 | 3.09331E-05 | IL12A/IL12B/IL23A |
| BP | GO:0097530 | granulocyte migration | 5/29 | 7.39119E-05 | 3.52388E-05 | DAPK2/LBP/RARRES2/XCL2/IL23A |
| BP | GO:0002705 | positive regulation of leukocyte mediated immunity | 5/29 | 7.69978E-05 | 3.671E-05 | CRTAM/IL12A/IL12B/IL23A/RIGI |
| BP | GO:0001912 | positive regulation of leukocyte mediated cytotoxicity | 4/29 | 7.85466E-05 | 3.74484E-05 | CRTAM/IL12A/IL12B/IL23A |
| BP | GO:0097696 | cell surface receptor signaling pathway via STAT | 5/29 | 7.85466E-05 | 3.74484E-05 | CTF1/IL12A/IL12B/IL7R/IL23A |
| BP | GO:0032816 | positive regulation of natural killer cell activation | 3/29 | 9.16257E-05 | 4.36841E-05 | IL12A/IL12B/IL23A |
| BP | GO:0046651 | lymphocyte proliferation | 6/29 | 9.16257E-05 | 4.36841E-05 | CRTAM/IL12A/IL12B/IL7R/IL2RA/IL23A |
| BP | GO:0045088 | regulation of innate immune response | 7/29 | 9.74879E-05 | 4.6479E-05 | CRTAM/IL12A/IL12B/LBP/MMP12/TNFAIP3/RIGI |
| BP | GO:0032729 | positive regulation of type II interferon production | 4/29 | 9.91845E-05 | 4.72879E-05 | CRTAM/IL12A/IL12B/IL23A |
| BP | GO:0032943 | mononuclear cell proliferation | 6/29 | 0.000100638 | 4.79809E-05 | CRTAM/IL12A/IL12B/IL7R/IL2RA/IL23A |
| BP | GO:0031343 | positive regulation of cell killing | 4/29 | 0.000100638 | 4.79809E-05 | CRTAM/IL12A/IL12B/IL23A |
| BP | GO:0090023 | positive regulation of neutrophil chemotaxis | 3/29 | 0.00012299 | 5.86375E-05 | DAPK2/LBP/IL23A |
| BP | GO:0030217 | T cell differentiation | 6/29 | 0.000125665 | 5.9913E-05 | CD8A/CRTAM/IL12B/IL7R/IL2RA/IL23A |
| BP | GO:0060326 | cell chemotaxis | 6/29 | 0.000138747 | 6.61498E-05 | DAPK2/IL12A/LBP/RARRES2/XCL2/IL23A |
| BP | GO:0032640 | tumor necrosis factor production | 5/29 | 0.000138747 | 6.61498E-05 | IL12B/LBP/TNFAIP3/IL23A/RIGI |
| BP | GO:0032680 | regulation of tumor necrosis factor production | 5/29 | 0.000138747 | 6.61498E-05 | IL12B/LBP/TNFAIP3/IL23A/RIGI |
| BP | GO:0002837 | regulation of immune response to tumor cell | 3/29 | 0.000138747 | 6.61498E-05 | CRTAM/IL12A/IL12B |
| BP | GO:0042104 | positive regulation of activated T cell proliferation | 3/29 | 0.000138747 | 6.61498E-05 | IL12B/IL2RA/IL23A |
| BP | GO:0071624 | positive regulation of granulocyte chemotaxis | 3/29 | 0.000138747 | 6.61498E-05 | DAPK2/LBP/IL23A |
| BP | GO:0045580 | regulation of T cell differentiation | 5/29 | 0.000142549 | 6.79629E-05 | CRTAM/IL12B/IL7R/IL2RA/IL23A |
| BP | GO:0071706 | tumor necrosis factor superfamily cytokine production | 5/29 | 0.000145393 | 6.93185E-05 | IL12B/LBP/TNFAIP3/IL23A/RIGI |
| BP | GO:1903555 | regulation of tumor necrosis factor superfamily cytokine production | 5/29 | 0.000145393 | 6.93185E-05 | IL12B/LBP/TNFAIP3/IL23A/RIGI |
| BP | GO:0002834 | regulation of response to tumor cell | 3/29 | 0.000145969 | 6.95931E-05 | CRTAM/IL12A/IL12B |
| BP | GO:0046631 | alpha-beta T cell activation | 5/29 | 0.00015207 | 7.25021E-05 | CRTAM/IL12A/IL12B/IL2RA/IL23A |
| BP | GO:0002706 | regulation of lymphocyte mediated immunity | 5/29 | 0.000155674 | 7.42201E-05 | CRTAM/IL12A/IL12B/IL7R/IL23A |
| BP | GO:0045954 | positive regulation of natural killer cell mediated cytotoxicity | 3/29 | 0.000155674 | 7.42201E-05 | CRTAM/IL12A/IL12B |
| BP | GO:0002822 | regulation of adaptive immune response based on somatic recombination of immune receptors built from immunoglobulin superfamily domains | 5/29 | 0.00018529 | 8.834E-05 | IL12A/IL12B/IL7R/TNFAIP3/IL23A |
| BP | GO:0002230 | positive regulation of defense response to virus by host | 3/29 | 0.0002243 | 0.000106939 | IL12B/IL23A/RIGI |
| BP | GO:0050920 | regulation of chemotaxis | 5/29 | 0.0002243 | 0.000106939 | DAPK2/IL12A/LBP/RARRES2/IL23A |
| BP | GO:0002696 | positive regulation of leukocyte activation | 6/29 | 0.0002243 | 0.000106939 | IL12A/IL12B/IL7R/LBP/IL2RA/IL23A |
| BP | GO:0002418 | immune response to tumor cell | 3/29 | 0.000233611 | 0.000111378 | CRTAM/IL12A/IL12B |
| BP | GO:0090022 | regulation of neutrophil chemotaxis | 3/29 | 0.000233611 | 0.000111378 | DAPK2/LBP/IL23A |
| BP | GO:0002819 | regulation of adaptive immune response | 5/29 | 0.000239777 | 0.000114318 | IL12A/IL12B/IL7R/TNFAIP3/IL23A |
| BP | GO:0045619 | regulation of lymphocyte differentiation | 5/29 | 0.000239782 | 0.00011432 | CRTAM/IL12B/IL7R/IL2RA/IL23A |
| BP | GO:0002717 | positive regulation of natural killer cell mediated immunity | 3/29 | 0.000239782 | 0.00011432 | CRTAM/IL12A/IL12B |
| BP | GO:1902624 | positive regulation of neutrophil migration | 3/29 | 0.000239782 | 0.00011432 | DAPK2/LBP/IL23A |
| BP | GO:0050829 | defense response to Gram-negative bacterium | 4/29 | 0.000239782 | 0.00011432 | IL12B/LBP/RARRES2/IL23A |
| BP | GO:0002709 | regulation of T cell mediated immunity | 4/29 | 0.000242748 | 0.000115734 | IL12A/IL12B/IL7R/IL23A |
| BP | GO:0032760 | positive regulation of tumor necrosis factor production | 4/29 | 0.000242748 | 0.000115734 | IL12B/LBP/IL23A/RIGI |
| BP | GO:0001818 | negative regulation of cytokine production | 6/29 | 0.000254428 | 0.000121303 | IL12A/IL12B/LBP/NFKB1/TNFAIP3/IL23A |
| BP | GO:0050867 | positive regulation of cell activation | 6/29 | 0.000262579 | 0.000125189 | IL12A/IL12B/IL7R/LBP/IL2RA/IL23A |
| BP | GO:1903037 | regulation of leukocyte cell-cell adhesion | 6/29 | 0.000263283 | 0.000125525 | CRTAM/IL12A/IL12B/IL7R/IL2RA/IL23A |
| BP | GO:0050863 | regulation of T cell activation | 6/29 | 0.000264015 | 0.000125874 | CRTAM/IL12A/IL12B/IL7R/IL2RA/IL23A |
| BP | GO:1903557 | positive regulation of tumor necrosis factor superfamily cytokine production | 4/29 | 0.000264227 | 0.000125975 | IL12B/LBP/IL23A/RIGI |
| BP | GO:0009595 | detection of biotic stimulus | 3/29 | 0.000303105 | 0.000144511 | CRTAM/FAP/LBP |
| BP | GO:0050670 | regulation of lymphocyte proliferation | 5/29 | 0.00030479 | 0.000145314 | CRTAM/IL12A/IL12B/IL2RA/IL23A |
| BP | GO:0097529 | myeloid leukocyte migration | 5/29 | 0.000316671 | 0.000150978 | DAPK2/LBP/RARRES2/XCL2/IL23A |
| BP | GO:0032814 | regulation of natural killer cell activation | 3/29 | 0.000316671 | 0.000150978 | IL12A/IL12B/IL23A |
| BP | GO:0032944 | regulation of mononuclear cell proliferation | 5/29 | 0.000325545 | 0.000155209 | CRTAM/IL12A/IL12B/IL2RA/IL23A |
| BP | GO:0001916 | positive regulation of T cell mediated cytotoxicity | 3/29 | 0.000334058 | 0.000159268 | IL12A/IL12B/IL23A |
| BP | GO:0045582 | positive regulation of T cell differentiation | 4/29 | 0.000342509 | 0.000163297 | IL12B/IL7R/IL2RA/IL23A |
| BP | GO:0032609 | type II interferon production | 4/29 | 0.000357525 | 0.000170456 | CRTAM/IL12A/IL12B/IL23A |
| BP | GO:0032649 | regulation of type II interferon production | 4/29 | 0.000357525 | 0.000170456 | CRTAM/IL12A/IL12B/IL23A |
| BP | GO:0030098 | lymphocyte differentiation | 6/29 | 0.000366451 | 0.000174712 | CD8A/CRTAM/IL12B/IL7R/IL2RA/IL23A |
| BP | GO:0046640 | regulation of alpha-beta T cell proliferation | 3/29 | 0.000366451 | 0.000174712 | IL12B/IL2RA/IL23A |
| BP | GO:0050870 | positive regulation of T cell activation | 5/29 | 0.000402045 | 0.000191682 | IL12A/IL12B/IL7R/IL2RA/IL23A |
| BP | GO:0032102 | negative regulation of response to external stimulus | 6/29 | 0.000409078 | 0.000195035 | FAP/IL12B/MMP12/NFKB1/TNFAIP3/IL2RA |
| BP | GO:0050830 | defense response to Gram-positive bacterium | 4/29 | 0.000409078 | 0.000195035 | IL12A/IL7R/LBP/RARRES2 |
| BP | GO:0050727 | regulation of inflammatory response | 6/29 | 0.000409275 | 0.000195129 | IL12B/LBP/NFKB1/TNFAIP3/IL2RA/IL23A |
| BP | GO:0046633 | alpha-beta T cell proliferation | 3/29 | 0.000427762 | 0.000203943 | IL12B/IL2RA/IL23A |
| BP | GO:1902622 | regulation of neutrophil migration | 3/29 | 0.000452111 | 0.000215552 | DAPK2/LBP/IL23A |
| BP | GO:0002708 | positive regulation of lymphocyte mediated immunity | 4/29 | 0.000469501 | 0.000223843 | CRTAM/IL12A/IL12B/IL23A |
| BP | GO:0045621 | positive regulation of lymphocyte differentiation | 4/29 | 0.000478596 | 0.000228179 | IL12B/IL7R/IL2RA/IL23A |
| BP | GO:0032620 | interleukin-17 production | 3/29 | 0.000489347 | 0.000233305 | IL12A/IL12B/IL23A |
| BP | GO:0032660 | regulation of interleukin-17 production | 3/29 | 0.000489347 | 0.000233305 | IL12A/IL12B/IL23A |
| BP | GO:0042269 | regulation of natural killer cell mediated cytotoxicity | 3/29 | 0.000489347 | 0.000233305 | CRTAM/IL12A/IL12B |
| BP | GO:0002347 | response to tumor cell | 3/29 | 0.000515363 | 0.000245708 | CRTAM/IL12A/IL12B |
| BP | GO:1903039 | positive regulation of leukocyte cell-cell adhesion | 5/29 | 0.000545984 | 0.000260307 | IL12A/IL12B/IL7R/IL2RA/IL23A |
| BP | GO:0006935 | chemotaxis | 6/29 | 0.000560329 | 0.000267147 | DAPK2/IL12A/LBP/RARRES2/XCL2/IL23A |
| BP | GO:0050671 | positive regulation of lymphocyte proliferation | 4/29 | 0.000560329 | 0.000267147 | IL12A/IL12B/IL2RA/IL23A |
| BP | GO:0042330 | taxis | 6/29 | 0.000568462 | 0.000271024 | DAPK2/IL12A/LBP/RARRES2/XCL2/IL23A |
| BP | GO:0032946 | positive regulation of mononuclear cell proliferation | 4/29 | 0.000596296 | 0.000284294 | IL12A/IL12B/IL2RA/IL23A |
| BP | GO:0031348 | negative regulation of defense response | 5/29 | 0.000645581 | 0.000307792 | IL12B/MMP12/NFKB1/TNFAIP3/IL2RA |
| BP | GO:0002715 | regulation of natural killer cell mediated immunity | 3/29 | 0.000645581 | 0.000307792 | CRTAM/IL12A/IL12B |
| BP | GO:0032819 | positive regulation of natural killer cell proliferation | 2/29 | 0.00080824 | 0.000385342 | IL12B/IL23A |
| BP | GO:0051607 | defense response to virus | 5/29 | 0.0008917 | 0.000425133 | IL12B/MMP12/TNFAIP3/IL23A/RIGI |
| BP | GO:0070665 | positive regulation of leukocyte proliferation | 4/29 | 0.0008917 | 0.000425133 | IL12A/IL12B/IL2RA/IL23A |
| BP | GO:0071349 | cellular response to interleukin-12 | 2/29 | 0.000962645 | 0.000458957 | IL12A/IL12B |
| BP | GO:0032615 | interleukin-12 production | 3/29 | 0.001012408 | 0.000482683 | IL12B/NFKB1/IL23A |
| BP | GO:0032655 | regulation of interleukin-12 production | 3/29 | 0.001012408 | 0.000482683 | IL12B/NFKB1/IL23A |
| BP | GO:0022409 | positive regulation of cell-cell adhesion | 5/29 | 0.001070572 | 0.000510414 | IL12A/IL12B/IL7R/IL2RA/IL23A |
| BP | GO:0051251 | positive regulation of lymphocyte activation | 5/29 | 0.001070572 | 0.000510414 | IL12A/IL12B/IL7R/IL2RA/IL23A |
| BP | GO:1902105 | regulation of leukocyte differentiation | 5/29 | 0.001074791 | 0.000512425 | CRTAM/IL12B/IL7R/IL2RA/IL23A |
| BP | GO:0032817 | regulation of natural killer cell proliferation | 2/29 | 0.001074791 | 0.000512425 | IL12B/IL23A |
| BP | GO:0043380 | regulation of memory T cell differentiation | 2/29 | 0.001074791 | 0.000512425 | IL12B/IL23A |
| BP | GO:0070671 | response to interleukin-12 | 2/29 | 0.001074791 | 0.000512425 | IL12A/IL12B |
| BP | GO:2000321 | positive regulation of T-helper 17 cell differentiation | 2/29 | 0.001074791 | 0.000512425 | IL12B/IL23A |
| BP | GO:0042129 | regulation of T cell proliferation | 4/29 | 0.001165088 | 0.000555476 | CRTAM/IL12B/IL2RA/IL23A |
| BP | GO:0048662 | negative regulation of smooth muscle cell proliferation | 3/29 | 0.001176847 | 0.000561082 | IL12A/IL12B/TNFAIP3 |
| BP | GO:0043379 | memory T cell differentiation | 2/29 | 0.001240594 | 0.000591475 | IL12B/IL23A |
| BP | GO:0032496 | response to lipopolysaccharide | 5/29 | 0.00127455 | 0.000607664 | IL12A/IL12B/LBP/NFKB1/TNFAIP3 |
| BP | GO:0048771 | tissue remodeling | 4/29 | 0.001360836 | 0.000648802 | IL12B/NFKB1/TNFAIP3/IL23A |
| BP | GO:0002711 | positive regulation of T cell mediated immunity | 3/29 | 0.001384018 | 0.000659854 | IL12A/IL12B/IL23A |
| BP | GO:0046635 | positive regulation of alpha-beta T cell activation | 3/29 | 0.001384018 | 0.000659854 | IL12A/IL12B/IL23A |
| BP | GO:0090715 | immunological memory formation process | 2/29 | 0.001384018 | 0.000659854 | IL12B/IL23A |
| BP | GO:2000328 | regulation of T-helper 17 cell lineage commitment | 2/29 | 0.001384018 | 0.000659854 | IL12B/IL23A |
| BP | GO:1902107 | positive regulation of leukocyte differentiation | 4/29 | 0.001410957 | 0.000672698 | IL12B/IL7R/IL2RA/IL23A |
| BP | GO:1903708 | positive regulation of hemopoiesis | 4/29 | 0.001410957 | 0.000672698 | IL12B/IL7R/IL2RA/IL23A |
| BP | GO:0001787 | natural killer cell proliferation | 2/29 | 0.001550583 | 0.000739267 | IL12B/IL23A |
| BP | GO:0042267 | natural killer cell mediated cytotoxicity | 3/29 | 0.001550583 | 0.000739267 | CRTAM/IL12A/IL12B |
| BP | GO:0002237 | response to molecule of bacterial origin | 5/29 | 0.001550583 | 0.000739267 | IL12A/IL12B/LBP/NFKB1/TNFAIP3 |
| BP | GO:0050728 | negative regulation of inflammatory response | 4/29 | 0.001600171 | 0.000762909 | IL12B/NFKB1/TNFAIP3/IL2RA |
| BP | GO:0002260 | lymphocyte homeostasis | 3/29 | 0.001640969 | 0.00078236 | IL7R/TNFAIP3/IL2RA |
| BP | GO:0050777 | negative regulation of immune response | 4/29 | 0.001700493 | 0.000810739 | IL12B/IL7R/MMP12/TNFAIP3 |
| BP | GO:0002228 | natural killer cell mediated immunity | 3/29 | 0.001790438 | 0.000853622 | CRTAM/IL12A/IL12B |
| BP | GO:2000514 | regulation of CD4-positive, alpha-beta T cell activation | 3/29 | 0.001790438 | 0.000853622 | IL12B/IL2RA/IL23A |
| BP | GO:0030593 | neutrophil chemotaxis | 3/29 | 0.001841761 | 0.000878092 | DAPK2/LBP/IL23A |
| BP | GO:0010455 | positive regulation of cell fate commitment | 2/29 | 0.001887301 | 0.000899803 | IL12B/IL23A |
| BP | GO:1900747 | negative regulation of vascular endothelial growth factor signaling pathway | 2/29 | 0.001887301 | 0.000899803 | IL12A/IL12B |
| BP | GO:0042098 | T cell proliferation | 4/29 | 0.001887994 | 0.000900134 | CRTAM/IL12B/IL2RA/IL23A |
| BP | GO:0034103 | regulation of tissue remodeling | 3/29 | 0.001987315 | 0.000947487 | IL12B/TNFAIP3/IL23A |
| BP | GO:0098586 | cellular response to virus | 3/29 | 0.002041362 | 0.000973255 | IL12A/MMP12/NFKB1 |
| BP | GO:0002827 | positive regulation of T-helper 1 type immune response | 2/29 | 0.002067159 | 0.000985554 | IL12B/IL23A |
| BP | GO:1902548 | negative regulation of cellular response to vascular endothelial growth factor stimulus | 2/29 | 0.002284856 | 0.001089345 | IL12A/IL12B |
| BP | GO:0071222 | cellular response to lipopolysaccharide | 4/29 | 0.002284856 | 0.001089345 | IL12B/LBP/NFKB1/TNFAIP3 |
| BP | GO:0071674 | mononuclear cell migration | 4/29 | 0.002385732 | 0.001137439 | CRTAM/ICAM1/IL12A/RARRES2 |
| BP | GO:0034393 | positive regulation of smooth muscle cell apoptotic process | 2/29 | 0.002483121 | 0.001183871 | IL12A/IL12B |
| BP | GO:2000318 | positive regulation of T-helper 17 type immune response | 2/29 | 0.002483121 | 0.001183871 | IL12B/IL23A |
| BP | GO:0070664 | negative regulation of leukocyte proliferation | 3/29 | 0.002655657 | 0.00126613 | CRTAM/TNFAIP3/IL2RA |
| BP | GO:1903706 | regulation of hemopoiesis | 5/29 | 0.002655657 | 0.00126613 | CRTAM/IL12B/IL7R/IL2RA/IL23A |
| BP | GO:0010224 | response to UV-B | 2/29 | 0.002675833 | 0.00127575 | IL12A/IL12B |
| BP | GO:0090713 | immunological memory process | 2/29 | 0.002675833 | 0.00127575 | IL12B/IL23A |
| BP | GO:0071219 | cellular response to molecule of bacterial origin | 4/29 | 0.002724725 | 0.00129906 | IL12B/LBP/NFKB1/TNFAIP3 |
| BP | GO:0030101 | natural killer cell activation | 3/29 | 0.002827985 | 0.001348291 | IL12A/IL12B/IL23A |
| BP | GO:0042102 | positive regulation of T cell proliferation | 3/29 | 0.002975491 | 0.001418617 | IL12B/IL2RA/IL23A |
| BP | GO:0032700 | negative regulation of interleukin-17 production | 2/29 | 0.003105398 | 0.001480552 | IL12A/IL12B |
| BP | GO:0034105 | positive regulation of tissue remodeling | 2/29 | 0.003105398 | 0.001480552 | IL12B/IL23A |
| BP | GO:0071359 | cellular response to dsRNA | 2/29 | 0.003105398 | 0.001480552 | NFKB1/RIGI |
| BP | GO:1990266 | neutrophil migration | 3/29 | 0.003245351 | 0.001547277 | DAPK2/LBP/IL23A |
| BP | GO:0046641 | positive regulation of alpha-beta T cell proliferation | 2/29 | 0.00332648 | 0.001585957 | IL12B/IL23A |
| BP | GO:0072540 | T-helper 17 cell lineage commitment | 2/29 | 0.00332648 | 0.001585957 | IL12B/IL23A |
| BP | GO:0030203 | glycosaminoglycan metabolic process | 3/29 | 0.003455324 | 0.001647385 | B3GNT2/NFKB1/SGSH |
| BP | GO:0032693 | negative regulation of interleukin-10 production | 2/29 | 0.003531595 | 0.001683749 | IL12B/IL23A |
| BP | GO:0032727 | positive regulation of interferon-alpha production | 2/29 | 0.003531595 | 0.001683749 | MMP12/RIGI |
| BP | GO:0042531 | positive regulation of tyrosine phosphorylation of STAT protein | 2/29 | 0.003531595 | 0.001683749 | CTF1/IL12A |
| BP | GO:0001776 | leukocyte homeostasis | 3/29 | 0.003558903 | 0.001696768 | IL7R/TNFAIP3/IL2RA |
| BP | GO:0071216 | cellular response to biotic stimulus | 4/29 | 0.003738726 | 0.001782502 | IL12B/LBP/NFKB1/TNFAIP3 |
| BP | GO:0045624 | positive regulation of T-helper cell differentiation | 2/29 | 0.003738726 | 0.001782502 | IL12B/IL23A |
| BP | GO:1903588 | negative regulation of blood vessel endothelial cell proliferation involved in sprouting angiogenesis | 2/29 | 0.003738726 | 0.001782502 | IL12A/IL12B |
| BP | GO:0050673 | epithelial cell proliferation | 5/29 | 0.003849961 | 0.001835535 | FAP/IL12A/IL12B/MMP12/TNFAIP3 |
| BP | GO:0048245 | eosinophil chemotaxis | 2/29 | 0.003990392 | 0.001902488 | DAPK2/XCL2 |
| BP | GO:0002295 | T-helper cell lineage commitment | 2/29 | 0.004224769 | 0.002014232 | IL12B/IL23A |
| BP | GO:0032740 | positive regulation of interleukin-17 production | 2/29 | 0.004224769 | 0.002014232 | IL12B/IL23A |
| BP | GO:2000319 | regulation of T-helper 17 cell differentiation | 2/29 | 0.004224769 | 0.002014232 | IL12B/IL23A |
| BP | GO:0006022 | aminoglycan metabolic process | 3/29 | 0.004234767 | 0.002018998 | B3GNT2/NFKB1/SGSH |
| BP | GO:0045785 | positive regulation of cell adhesion | 5/29 | 0.004357787 | 0.00207765 | IL12A/IL12B/IL7R/IL2RA/IL23A |
| BP | GO:0002507 | tolerance induction | 2/29 | 0.00470256 | 0.002242027 | TNFAIP3/IL2RA |
| BP | GO:0042509 | regulation of tyrosine phosphorylation of STAT protein | 2/29 | 0.00470256 | 0.002242027 | CTF1/IL12A |
| BP | GO:0045672 | positive regulation of osteoclast differentiation | 2/29 | 0.00470256 | 0.002242027 | IL12B/IL23A |
| BP | GO:1900746 | regulation of vascular endothelial growth factor signaling pathway | 2/29 | 0.00470256 | 0.002242027 | IL12A/IL12B |
| BP | GO:0035710 | CD4-positive, alpha-beta T cell activation | 3/29 | 0.00482414 | 0.002299992 | IL12B/IL2RA/IL23A |
| BP | GO:0002825 | regulation of T-helper 1 type immune response | 2/29 | 0.004871455 | 0.00232255 | IL12B/IL23A |
| BP | GO:0032607 | interferon-alpha production | 2/29 | 0.004871455 | 0.00232255 | MMP12/RIGI |
| BP | GO:0032647 | regulation of interferon-alpha production | 2/29 | 0.004871455 | 0.00232255 | MMP12/RIGI |
| BP | GO:0043373 | CD4-positive, alpha-beta T cell lineage commitment | 2/29 | 0.004871455 | 0.00232255 | IL12B/IL23A |
| BP | GO:0072677 | eosinophil migration | 2/29 | 0.004871455 | 0.00232255 | DAPK2/XCL2 |
| BP | GO:0002824 | positive regulation of adaptive immune response based on somatic recombination of immune receptors built from immunoglobulin superfamily domains | 3/29 | 0.005111789 | 0.002437133 | IL12A/IL12B/IL23A |
| BP | GO:1902042 | negative regulation of extrinsic apoptotic signaling pathway via death domain receptors | 2/29 | 0.005114585 | 0.002438466 | ICAM1/TNFAIP3 |
| BP | GO:1902547 | regulation of cellular response to vascular endothelial growth factor stimulus | 2/29 | 0.005114585 | 0.002438466 | IL12A/IL12B |
| BP | GO:0038061 | non-canonical NF-kappaB signal transduction | 3/29 | 0.005146685 | 0.002453771 | IL12B/NFKB1/IL23A |
| BP | GO:0002286 | T cell activation involved in immune response | 3/29 | 0.005345171 | 0.002548402 | ICAM1/IL12B/IL23A |
| BP | GO:0002363 | alpha-beta T cell lineage commitment | 2/29 | 0.005360193 | 0.002555564 | IL12B/IL23A |
| BP | GO:0002821 | positive regulation of adaptive immune response | 3/29 | 0.005635109 | 0.002686635 | IL12A/IL12B/IL23A |
| BP | GO:0006516 | glycoprotein catabolic process | 2/29 | 0.005635109 | 0.002686635 | MMP12/SGSH |
| BP | GO:0002366 | leukocyte activation involved in immune response | 4/29 | 0.005820904 | 0.002775216 | ICAM1/IL12B/LBP/IL23A |
| BP | GO:0007260 | tyrosine phosphorylation of STAT protein | 2/29 | 0.005886083 | 0.002806291 | CTF1/IL12A |
| BP | GO:0043369 | CD4-positive or CD8-positive, alpha-beta T cell lineage commitment | 2/29 | 0.005886083 | 0.002806291 | IL12B/IL23A |
| BP | GO:0002263 | cell activation involved in immune response | 4/29 | 0.006011683 | 0.002866174 | ICAM1/IL12B/LBP/IL23A |
| BP | GO:0001782 | B cell homeostasis | 2/29 | 0.006110589 | 0.002913328 | IL7R/TNFAIP3 |
| BP | GO:0034143 | regulation of toll-like receptor 4 signaling pathway | 2/29 | 0.006110589 | 0.002913328 | LBP/TNFAIP3 |
| BP | GO:2000352 | negative regulation of endothelial cell apoptotic process | 2/29 | 0.006110589 | 0.002913328 | ICAM1/TNFAIP3 |
| BP | GO:0010661 | positive regulation of muscle cell apoptotic process | 2/29 | 0.006423779 | 0.003062647 | IL12A/IL12B |
| BP | GO:0034391 | regulation of smooth muscle cell apoptotic process | 2/29 | 0.006681446 | 0.003185495 | IL12A/IL12B |
| BP | GO:0043372 | positive regulation of CD4-positive, alpha-beta T cell differentiation | 2/29 | 0.006681446 | 0.003185495 | IL12B/IL23A |
| BP | GO:2000316 | regulation of T-helper 17 type immune response | 2/29 | 0.006681446 | 0.003185495 | IL12B/IL23A |
| BP | GO:0034390 | smooth muscle cell apoptotic process | 2/29 | 0.006978694 | 0.003327213 | IL12A/IL12B |
| BP | GO:0002429 | immune response-activating cell surface receptor signaling pathway | 4/29 | 0.006978694 | 0.003327213 | CD8A/LBP/NFKB1/TNFAIP3 |
| BP | GO:0072539 | T-helper 17 cell differentiation | 2/29 | 0.007299269 | 0.003480052 | IL12B/IL23A |
| BP | GO:0002360 | T cell lineage commitment | 2/29 | 0.007631958 | 0.003638668 | IL12B/IL23A |
| BP | GO:0032733 | positive regulation of interleukin-10 production | 2/29 | 0.007970241 | 0.00379995 | IL12B/IL23A |
| BP | GO:0050729 | positive regulation of inflammatory response | 3/29 | 0.00804376 | 0.003835001 | IL12B/LBP/IL23A |
| BP | GO:0032735 | positive regulation of interleukin-12 production | 2/29 | 0.008663187 | 0.004130324 | IL12B/IL23A |
| BP | GO:0051250 | negative regulation of lymphocyte activation | 3/29 | 0.008978107 | 0.004280467 | CRTAM/TNFAIP3/IL2RA |
| BP | GO:0030574 | collagen catabolic process | 2/29 | 0.008978107 | 0.004280467 | FAP/MMP12 |
| BP | GO:0002768 | immune response-regulating cell surface receptor signaling pathway | 4/29 | 0.009477303 | 0.004518467 | CD8A/LBP/NFKB1/TNFAIP3 |
| BP | GO:0048660 | regulation of smooth muscle cell proliferation | 3/29 | 0.009643745 | 0.004597822 | IL12A/IL12B/TNFAIP3 |
| BP | GO:0007157 | heterophilic cell-cell adhesion via plasma membrane cell adhesion molecules | 2/29 | 0.009824105 | 0.004683811 | CRTAM/ICAM1 |
| BP | GO:0010453 | regulation of cell fate commitment | 2/29 | 0.009824105 | 0.004683811 | IL12B/IL23A |
| BP | GO:0045622 | regulation of T-helper cell differentiation | 2/29 | 0.009824105 | 0.004683811 | IL12B/IL23A |
| BP | GO:1903587 | regulation of blood vessel endothelial cell proliferation involved in sprouting angiogenesis | 2/29 | 0.009824105 | 0.004683811 | IL12A/IL12B |
| BP | GO:2000516 | positive regulation of CD4-positive, alpha-beta T cell activation | 2/29 | 0.009824105 | 0.004683811 | IL12B/IL23A |
| BP | GO:0032635 | interleukin-6 production | 3/29 | 0.009824105 | 0.004683811 | LBP/TNFAIP3/RIGI |
| BP | GO:0032675 | regulation of interleukin-6 production | 3/29 | 0.009824105 | 0.004683811 | LBP/TNFAIP3/RIGI |
| BP | GO:0048659 | smooth muscle cell proliferation | 3/29 | 0.009940362 | 0.004739239 | IL12A/IL12B/TNFAIP3 |
| BP | GO:0042088 | T-helper 1 type immune response | 2/29 | 0.010051541 | 0.004792245 | IL12B/IL23A |
| BP | GO:0043029 | T cell homeostasis | 2/29 | 0.010051541 | 0.004792245 | IL7R/IL2RA |
| BP | GO:1902041 | regulation of extrinsic apoptotic signaling pathway via death domain receptors | 2/29 | 0.010051541 | 0.004792245 | ICAM1/TNFAIP3 |
| BP | GO:0043368 | positive T cell selection | 2/29 | 0.01041724 | 0.004966599 | IL12B/IL23A |
| BP | GO:0038084 | vascular endothelial growth factor signaling pathway | 2/29 | 0.010743404 | 0.005122103 | IL12A/IL12B |
| BP | GO:1900047 | negative regulation of hemostasis | 2/29 | 0.010743404 | 0.005122103 | FAP/COMP |
| BP | GO:0072538 | T-helper 17 type immune response | 2/29 | 0.011974686 | 0.005709138 | IL12B/IL23A |
| BP | GO:0002043 | blood vessel endothelial cell proliferation involved in sprouting angiogenesis | 2/29 | 0.01236436 | 0.005894922 | IL12A/IL12B |
| BP | GO:0050678 | regulation of epithelial cell proliferation | 4/29 | 0.012403693 | 0.005913674 | IL12A/IL12B/MMP12/TNFAIP3 |
| BP | GO:0002695 | negative regulation of leukocyte activation | 3/29 | 0.012523399 | 0.005970746 | CRTAM/TNFAIP3/IL2RA |
| BP | GO:0046638 | positive regulation of alpha-beta T cell differentiation | 2/29 | 0.012605231 | 0.006009761 | IL12B/IL23A |
| BP | GO:1904645 | response to amyloid-beta | 2/29 | 0.012605231 | 0.006009761 | ICAM1/MMP12 |
| BP | GO:0060759 | regulation of response to cytokine stimulus | 3/29 | 0.012913999 | 0.006156972 | MMP12/TNFAIP3/RIGI |
| BP | GO:0043331 | response to dsRNA | 2/29 | 0.012947688 | 0.006173034 | NFKB1/RIGI |
| BP | GO:0031663 | lipopolysaccharide-mediated signaling pathway | 2/29 | 0.013309517 | 0.006345542 | LBP/TNFAIP3 |
| BP | GO:0002520 | immune system development | 3/29 | 0.013309517 | 0.006345542 | IL7R/TNFAIP3/IL2RA |
| BP | GO:0070231 | T cell apoptotic process | 2/29 | 0.014616062 | 0.006968459 | IL7R/IL2RA |
| BP | GO:0043370 | regulation of CD4-positive, alpha-beta T cell differentiation | 2/29 | 0.01503007 | 0.007165845 | IL12B/IL23A |
| BP | GO:0045058 | T cell selection | 2/29 | 0.015447868 | 0.007365037 | IL12B/IL23A |
| BP | GO:0002763 | positive regulation of myeloid leukocyte differentiation | 2/29 | 0.01574732 | 0.007507806 | IL12B/IL23A |
| BP | GO:0034142 | toll-like receptor 4 signaling pathway | 2/29 | 0.01574732 | 0.007507806 | LBP/TNFAIP3 |
| BP | GO:2000351 | regulation of endothelial cell apoptotic process | 2/29 | 0.01574732 | 0.007507806 | ICAM1/TNFAIP3 |
| BP | GO:0042060 | wound healing | 4/29 | 0.016046752 | 0.007650566 | FAP/MMP12/TNFAIP3/COMP |
| BP | GO:0022617 | extracellular matrix disassembly | 2/29 | 0.016046752 | 0.007650566 | FAP/MMP12 |
| BP | GO:0032757 | positive regulation of interleukin-8 production | 2/29 | 0.016046752 | 0.007650566 | LBP/RIGI |
| BP | GO:0050866 | negative regulation of cell activation | 3/29 | 0.016055836 | 0.007654896 | CRTAM/TNFAIP3/IL2RA |
| BP | GO:0032613 | interleukin-10 production | 2/29 | 0.016284923 | 0.007764118 | IL12B/IL23A |
| BP | GO:0032653 | regulation of interleukin-10 production | 2/29 | 0.016284923 | 0.007764118 | IL12B/IL23A |
| BP | GO:1901224 | positive regulation of non-canonical NF-kappaB signal transduction | 2/29 | 0.016284923 | 0.007764118 | IL12B/IL23A |
| BP | GO:0060760 | positive regulation of response to cytokine stimulus | 2/29 | 0.017197642 | 0.008199272 | MMP12/RIGI |
| BP | GO:0002285 | lymphocyte activation involved in immune response | 3/29 | 0.017413788 | 0.008302324 | ICAM1/IL12B/IL23A |
| BP | GO:0010720 | positive regulation of cell development | 4/29 | 0.017696411 | 0.008437069 | IL12B/IL7R/IL2RA/IL23A |
| BP | GO:0072577 | endothelial cell apoptotic process | 2/29 | 0.017996095 | 0.008579949 | ICAM1/TNFAIP3 |
| BP | GO:0006024 | glycosaminoglycan biosynthetic process | 2/29 | 0.018364727 | 0.0087557 | B3GNT2/NFKB1 |
| BP | GO:1900046 | regulation of hemostasis | 2/29 | 0.018364727 | 0.0087557 | FAP/COMP |
| BP | GO:0140895 | cell surface toll-like receptor signaling pathway | 2/29 | 0.018802748 | 0.008964534 | LBP/TNFAIP3 |
| BP | GO:0097191 | extrinsic apoptotic signaling pathway | 3/29 | 0.018975866 | 0.009047071 | ICAM1/IL12A/TNFAIP3 |
| BP | GO:0045670 | regulation of osteoclast differentiation | 2/29 | 0.019174218 | 0.009141639 | IL12B/IL23A |
| BP | GO:0006909 | phagocytosis | 3/29 | 0.019509774 | 0.009301621 | ICAM5/LBP/TIMD4 |
| BP | GO:0042130 | negative regulation of T cell proliferation | 2/29 | 0.019546615 | 0.009319185 | CRTAM/IL2RA |
| BP | GO:0006023 | aminoglycan biosynthetic process | 2/29 | 0.019919899 | 0.009497155 | B3GNT2/NFKB1 |
| BP | GO:0032481 | positive regulation of type I interferon production | 2/29 | 0.019919899 | 0.009497155 | MMP12/RIGI |
| BP | GO:0050709 | negative regulation of protein secretion | 2/29 | 0.020366254 | 0.009709963 | IL12A/IL12B |
| BP | GO:0035924 | cellular response to vascular endothelial growth factor stimulus | 2/29 | 0.021343215 | 0.010175746 | IL12A/IL12B |
| BP | GO:0001937 | negative regulation of endothelial cell proliferation | 2/29 | 0.021646665 | 0.010320421 | IL12A/IL12B |
| BP | GO:0002752 | cell surface pattern recognition receptor signaling pathway | 2/29 | 0.021646665 | 0.010320421 | LBP/TNFAIP3 |
| BP | GO:0032720 | negative regulation of tumor necrosis factor production | 2/29 | 0.021646665 | 0.010320421 | LBP/TNFAIP3 |
| BP | GO:0033002 | muscle cell proliferation | 3/29 | 0.021872386 | 0.010428037 | IL12A/IL12B/TNFAIP3 |
| BP | GO:0046637 | regulation of alpha-beta T cell differentiation | 2/29 | 0.022026218 | 0.010501379 | IL12B/IL23A |
| BP | GO:0042093 | T-helper cell differentiation | 2/29 | 0.022406423 | 0.010682648 | IL12B/IL23A |
| BP | GO:1903556 | negative regulation of tumor necrosis factor superfamily cytokine production | 2/29 | 0.022406423 | 0.010682648 | LBP/TNFAIP3 |
| BP | GO:0002294 | CD4-positive, alpha-beta T cell differentiation involved in immune response | 2/29 | 0.023408342 | 0.01116033 | IL12B/IL23A |
| BP | GO:0002287 | alpha-beta T cell activation involved in immune response | 2/29 | 0.023711399 | 0.011304818 | IL12B/IL23A |
| BP | GO:0002293 | alpha-beta T cell differentiation involved in immune response | 2/29 | 0.023711399 | 0.011304818 | IL12B/IL23A |
| BP | GO:0070227 | lymphocyte apoptotic process | 2/29 | 0.023711399 | 0.011304818 | IL7R/IL2RA |
| BP | GO:0002437 | inflammatory response to antigenic stimulus | 2/29 | 0.02417737 | 0.011526978 | IL12B/IL2RA |
| BP | GO:0008625 | extrinsic apoptotic signaling pathway via death domain receptors | 2/29 | 0.025202027 | 0.012015501 | ICAM1/TNFAIP3 |
| BP | GO:0050672 | negative regulation of lymphocyte proliferation | 2/29 | 0.026813485 | 0.012783791 | CRTAM/IL2RA |
| BP | GO:1903510 | mucopolysaccharide metabolic process | 2/29 | 0.027298002 | 0.013014793 | B3GNT2/NFKB1 |
| BP | GO:0002221 | pattern recognition receptor signaling pathway | 3/29 | 0.027475849 | 0.013099584 | LBP/TNFAIP3/RIGI |
| BP | GO:0002292 | T cell differentiation involved in immune response | 2/29 | 0.027599573 | 0.013158572 | IL12B/IL23A |
| BP | GO:0032945 | negative regulation of mononuclear cell proliferation | 2/29 | 0.027599573 | 0.013158572 | CRTAM/IL2RA |
| BP | GO:0045824 | negative regulation of innate immune response | 2/29 | 0.028085988 | 0.013390479 | MMP12/TNFAIP3 |
| BP | GO:0071677 | positive regulation of mononuclear cell migration | 2/29 | 0.028574612 | 0.013623439 | IL12A/RARRES2 |
| BP | GO:0010660 | regulation of muscle cell apoptotic process | 2/29 | 0.029065407 | 0.013857434 | IL12A/IL12B |
| BP | GO:0002220 | innate immune response activating cell surface receptor signaling pathway | 2/29 | 0.031965771 | 0.015240232 | LBP/TNFAIP3 |
| BP | GO:0002758 | innate immune response-activating signaling pathway | 3/29 | 0.032128935 | 0.015318023 | LBP/TNFAIP3/RIGI |
| BP | GO:0010657 | muscle cell apoptotic process | 2/29 | 0.032367922 | 0.015431964 | IL12A/IL12B |
| BP | GO:0043367 | CD4-positive, alpha-beta T cell differentiation | 2/29 | 0.032770044 | 0.015623683 | IL12B/IL23A |
| BP | GO:2001237 | negative regulation of extrinsic apoptotic signaling pathway | 2/29 | 0.032770044 | 0.015623683 | ICAM1/TNFAIP3 |
| BP | GO:0032755 | positive regulation of interleukin-6 production | 2/29 | 0.033172123 | 0.015815381 | LBP/RIGI |
| BP | GO:1990830 | cellular response to leukemia inhibitory factor | 2/29 | 0.033172123 | 0.015815381 | B3GNT2/ICAM1 |
| BP | GO:0032637 | interleukin-8 production | 2/29 | 0.03408464 | 0.016250439 | LBP/RIGI |
| BP | GO:0032677 | regulation of interleukin-8 production | 2/29 | 0.03408464 | 0.016250439 | LBP/RIGI |
| BP | GO:1990823 | response to leukemia inhibitory factor | 2/29 | 0.03408464 | 0.016250439 | B3GNT2/ICAM1 |
| BP | GO:1901222 | regulation of non-canonical NF-kappaB signal transduction | 2/29 | 0.034596917 | 0.016494676 | IL12B/IL23A |
| BP | GO:0045639 | positive regulation of myeloid cell differentiation | 2/29 | 0.035110941 | 0.016739746 | IL12B/IL23A |
| BP | GO:0007162 | negative regulation of cell adhesion | 3/29 | 0.036037071 | 0.017181294 | CRTAM/MMP12/IL2RA |
| BP | GO:0072676 | lymphocyte migration | 2/29 | 0.036737663 | 0.017515314 | CRTAM/ICAM1 |
| BP | GO:0002218 | activation of innate immune response | 3/29 | 0.036737663 | 0.017515314 | LBP/TNFAIP3/RIGI |
| BP | GO:0032963 | collagen metabolic process | 2/29 | 0.037183882 | 0.017728057 | FAP/MMP12 |
| BP | GO:0030316 | osteoclast differentiation | 2/29 | 0.037706173 | 0.017977068 | IL12B/IL23A |
| BP | GO:1905954 | positive regulation of lipid localization | 2/29 | 0.038230024 | 0.018226823 | NFKB1/DBI |
| BP | GO:0019882 | antigen processing and presentation | 2/29 | 0.041251293 | 0.019667265 | CD8A/ICAM1 |
| BP | GO:0050731 | positive regulation of peptidyl-tyrosine phosphorylation | 2/29 | 0.041251293 | 0.019667265 | CTF1/IL12A |
| BP | GO:0051224 | negative regulation of protein transport | 2/29 | 0.042664095 | 0.020340842 | IL12A/IL12B |
| BP | GO:0030198 | extracellular matrix organization | 3/29 | 0.042664095 | 0.020340842 | FAP/MMP12/COMP |
| BP | GO:0048872 | homeostasis of number of cells | 3/29 | 0.042664095 | 0.020340842 | IL7R/TNFAIP3/IL2RA |
| BP | GO:0043062 | extracellular structure organization | 3/29 | 0.042664095 | 0.020340842 | FAP/MMP12/COMP |
| BP | GO:0071887 | leukocyte apoptotic process | 2/29 | 0.042664095 | 0.020340842 | IL7R/IL2RA |
| BP | GO:0045229 | external encapsulating structure organization | 3/29 | 0.042664095 | 0.020340842 | FAP/MMP12/COMP |
| BP | GO:0001915 | negative regulation of T cell mediated cytotoxicity | 1/29 | 0.042664095 | 0.020340842 | IL7R |
| BP | GO:0002536 | respiratory burst involved in inflammatory response | 1/29 | 0.042664095 | 0.020340842 | LBP |
| BP | GO:0006924 | activation-induced cell death of T cells | 1/29 | 0.042664095 | 0.020340842 | IL2RA |
| BP | GO:0032353 | negative regulation of hormone biosynthetic process | 1/29 | 0.042664095 | 0.020340842 | NFKB1 |
| BP | GO:0042368 | vitamin D biosynthetic process | 1/29 | 0.042664095 | 0.020340842 | NFKB1 |
| BP | GO:0044650 | adhesion of symbiont to host cell | 1/29 | 0.042664095 | 0.020340842 | ICAM1 |
| BP | GO:0060054 | positive regulation of epithelial cell proliferation involved in wound healing | 1/29 | 0.042664095 | 0.020340842 | MMP12 |
| BP | GO:0070432 | regulation of nucleotide-binding oligomerization domain containing 2 signaling pathway | 1/29 | 0.042664095 | 0.020340842 | TNFAIP3 |
| BP | GO:0140367 | antibacterial innate immune response | 1/29 | 0.042664095 | 0.020340842 | NFKB1 |
| BP | GO:1905918 | regulation of CoA-transferase activity | 1/29 | 0.042664095 | 0.020340842 | DBI |
| BP | GO:2000508 | regulation of dendritic cell chemotaxis | 1/29 | 0.042664095 | 0.020340842 | IL12A |
| BP | GO:0002832 | negative regulation of response to biotic stimulus | 2/29 | 0.044776372 | 0.021347907 | MMP12/TNFAIP3 |
| BP | GO:0022612 | gland morphogenesis | 2/29 | 0.044776372 | 0.021347907 | NFKB1/TNFAIP3 |
| BP | GO:0032479 | regulation of type I interferon production | 2/29 | 0.04504132 | 0.021474225 | MMP12/RIGI |
| BP | GO:0032606 | type I interferon production | 2/29 | 0.04504132 | 0.021474225 | MMP12/RIGI |
| BP | GO:0002158 | osteoclast proliferation | 1/29 | 0.04504132 | 0.021474225 | TNFAIP3 |
| BP | GO:0002732 | positive regulation of dendritic cell cytokine production | 1/29 | 0.04504132 | 0.021474225 | RIGI |
| BP | GO:0030167 | proteoglycan catabolic process | 1/29 | 0.04504132 | 0.021474225 | SGSH |
| BP | GO:0033089 | positive regulation of T cell differentiation in thymus | 1/29 | 0.04504132 | 0.021474225 | IL7R |
| BP | GO:0035871 | protein K11-linked deubiquitination | 1/29 | 0.04504132 | 0.021474225 | TNFAIP3 |
| BP | GO:0070391 | response to lipoteichoic acid | 1/29 | 0.04504132 | 0.021474225 | LBP |
| BP | GO:0070669 | response to interleukin-2 | 1/29 | 0.04504132 | 0.021474225 | IL2RA |
| BP | GO:0071223 | cellular response to lipoteichoic acid | 1/29 | 0.04504132 | 0.021474225 | LBP |
| BP | GO:1903059 | regulation of protein lipidation | 1/29 | 0.04504132 | 0.021474225 | DBI |
| BP | GO:2000347 | positive regulation of hepatocyte proliferation | 1/29 | 0.04504132 | 0.021474225 | TNFAIP3 |
| BP | GO:0002761 | regulation of myeloid leukocyte differentiation | 2/29 | 0.045079829 | 0.021492585 | IL12B/IL23A |
| BP | GO:0046632 | alpha-beta T cell differentiation | 2/29 | 0.046927804 | 0.022373639 | IL12B/IL23A |
| BP | GO:0050868 | negative regulation of T cell activation | 2/29 | 0.047460829 | 0.022627768 | CRTAM/IL2RA |
| BP | GO:0019732 | antifungal humoral response | 1/29 | 0.047863382 | 0.022819692 | RARRES2 |
| BP | GO:0030656 | regulation of vitamin metabolic process | 1/29 | 0.047863382 | 0.022819692 | NFKB1 |
| BP | GO:0032351 | negative regulation of hormone metabolic process | 1/29 | 0.047863382 | 0.022819692 | NFKB1 |
| BP | GO:0034135 | regulation of toll-like receptor 2 signaling pathway | 1/29 | 0.047863382 | 0.022819692 | TNFAIP3 |
| BP | GO:0034139 | regulation of toll-like receptor 3 signaling pathway | 1/29 | 0.047863382 | 0.022819692 | TNFAIP3 |
| BP | GO:0097084 | vascular associated smooth muscle cell development | 1/29 | 0.047863382 | 0.022819692 | COMP |
| BP | GO:1904950 | negative regulation of establishment of protein localization | 2/29 | 0.047863382 | 0.022819692 | IL12A/IL12B |
| BP | GO:0009306 | protein secretion | 3/29 | 0.048634338 | 0.023187259 | IL12A/IL12B/COMP |
| BP | GO:0035592 | establishment of protein localization to extracellular region | 3/29 | 0.049458125 | 0.023580013 | IL12A/IL12B/COMP |
| BP | GO:0090288 | negative regulation of cellular response to growth factor stimulus | 2/29 | 0.049458125 | 0.023580013 | IL12A/IL12B |
| BP | GO:0061041 | regulation of wound healing | 2/29 | 0.049991653 | 0.023834382 | FAP/TNFAIP3 |
| CC | GO:0009897 | external side of plasma membrane | 5/29 | 0.013911407 | 0.01004952 | CD8A/ICAM1/IL12B/IL7R/IL2RA |
| CC | GO:0005788 | endoplasmic reticulum lumen | 4/29 | 0.022499403 | 0.016253439 | IL12A/IL12B/DBI/IL23A |
| CC | GO:0031904 | endosome lumen | 2/29 | 0.022499403 | 0.016253439 | IL12A/IL12B |
| CC | GO:0005775 | vacuolar lumen | 3/29 | 0.022499403 | 0.016253439 | DAPK2/GCA/SGSH |
| CC | GO:0001772 | immunological synapse | 2/29 | 0.022499403 | 0.016253439 | CRTAM/ICAM1 |
| MF | GO:0005125 | cytokine activity | 5/29 | 0.002486614 | 0.001846264 | CTF1/IL12A/IL12B/XCL2/IL23A |
| MF | GO:0005126 | cytokine receptor binding | 5/29 | 0.002486614 | 0.001846264 | CTF1/IL12A/IL12B/XCL2/IL23A |
| MF | GO:0019955 | cytokine binding | 4/29 | 0.002486614 | 0.001846264 | IL12A/IL12B/COMP/IL2RA |
| MF | GO:0005178 | integrin binding | 4/29 | 0.002953584 | 0.002192981 | FAP/ICAM1/ICAM5/COMP |
| MF | GO:0004896 | cytokine receptor activity | 3/29 | 0.009983713 | 0.007412719 | IL12B/IL7R/IL2RA |
| MF | GO:0002020 | protease binding | 3/29 | 0.022219743 | 0.016497742 | FAP/TNFAIP3/COMP |
| MF | GO:0140375 | immune receptor activity | 3/29 | 0.027212123 | 0.02020449 | IL12B/IL7R/IL2RA |
| MF | GO:0004252 | serine-type endopeptidase activity | 3/29 | 0.035959775 | 0.026699457 | FAP/MMP12/PRSS53 |
| MF | GO:0008236 | serine-type peptidase activity | 3/29 | 0.039403883 | 0.029256642 | FAP/MMP12/PRSS53 |
| MF | GO:0017171 | serine hydrolase activity | 3/29 | 0.039403883 | 0.029256642 | FAP/MMP12/PRSS53 |
| MF | GO:0015026 | coreceptor activity | 2/29 | 0.040727096 | 0.030239103 | CD8A/LBP |
| MF | GO:0005518 | collagen binding | 2/29 | 0.04749514 | 0.035264248 | MMP12/COMP |

The table showed the GO terms and the corresponding genes in enrichment analysis for each category of biological process (BP), cellular component (CC), and molecular function (MF).

**Table S8. The KEGG pathways of enrichment analysis.**

| **Category** | **subcategory** | **ID** | **Description** | **GeneRatio** | **p.adjust** | **qvalue** | **geneID** |
| --- | --- | --- | --- | --- | --- | --- | --- |
| Human Diseases | Infectious disease: viral | hsa05162 | Measles | 6/19 | 2.32985E-05 | 1.42106E-05 | IL12A/IL12B/NFKB1/TNFAIP3/IL2RA/RIGI |
| Environmental Information Processing | Signal transduction | hsa04630 | JAK-STAT signaling pathway | 6/19 | 3.57481E-05 | 2.1804E-05 | CTF1/IL12A/IL12B/IL7R/IL2RA/IL23A |
| Environmental Information Processing | Signaling molecules and interaction | hsa04060 | Cytokine-cytokine receptor interaction | 7/19 | 3.89344E-05 | 2.37475E-05 | CTF1/IL12A/IL12B/IL7R/XCL2/IL2RA/IL23A |
| Environmental Information Processing | Signal transduction | hsa04064 | NF-kappa B signaling pathway | 5/19 | 4.34707E-05 | 2.65144E-05 | ICAM1/LBP/NFKB1/TNFAIP3/RIGI |
| Human Diseases | Immune disease | hsa05321 | Inflammatory bowel disease | 4/19 | 0.000170074 | 0.000103734 | IL12A/IL12B/NFKB1/IL23A |
| Organismal Systems | Immune system | hsa04622 | RIG-I-like receptor signaling pathway | 4/19 | 0.00020076 | 0.000122451 | IL12A/IL12B/NFKB1/RIGI |
| Human Diseases | Infectious disease: bacterial | hsa05133 | Pertussis | 4/19 | 0.000236763 | 0.00014441 | IL12A/IL12B/NFKB1/IL23A |
| Human Diseases | Infectious disease: viral | hsa05164 | Influenza A | 5/19 | 0.000251834 | 0.000153603 | ICAM1/IL12A/IL12B/NFKB1/RIGI |
| Human Diseases | Infectious disease: bacterial | hsa05152 | Tuberculosis | 5/19 | 0.000286053 | 0.000174474 | IL12A/IL12B/LBP/NFKB1/IL23A |
| Organismal Systems | Immune system | hsa04658 | Th1 and Th2 cell differentiation | 4/19 | 0.000332779 | 0.000202974 | IL12A/IL12B/NFKB1/IL2RA |
| Organismal Systems | Immune system | hsa04625 | C-type lectin receptor signaling pathway | 4/19 | 0.000443711 | 0.000270635 | IL12A/IL12B/NFKB1/IL23A |
| Human Diseases | Infectious disease: parasitic | hsa05143 | African trypanosomiasis | 3/19 | 0.000443711 | 0.000270635 | ICAM1/IL12A/IL12B |
| Human Diseases | Cardiovascular disease | hsa05417 | Lipid and atherosclerosis | 5/19 | 0.000443711 | 0.000270635 | ICAM1/IL12A/IL12B/LBP/NFKB1 |
| Organismal Systems | Immune system | hsa04620 | Toll-like receptor signaling pathway | 4/19 | 0.000443711 | 0.000270635 | IL12A/IL12B/LBP/NFKB1 |
| Human Diseases | Infectious disease: parasitic | hsa05144 | Malaria | 3/19 | 0.000922793 | 0.000562844 | ICAM1/IL12A/COMP |
| Human Diseases | Endocrine and metabolic disease | hsa04936 | Alcoholic liver disease | 4/19 | 0.001142755 | 0.000697007 | IL12A/IL12B/LBP/NFKB1 |
| Human Diseases | Infectious disease: bacterial | hsa05134 | Legionellosis | 3/19 | 0.001142755 | 0.000697007 | IL12A/IL12B/NFKB1 |
| Human Diseases | Infectious disease: viral | hsa05168 | Herpes simplex virus 1 infection | 4/19 | 0.002497872 | 0.001523542 | IL12A/IL12B/NFKB1/RIGI |
| Human Diseases | Infectious disease: parasitic | hsa05140 | Leishmaniasis | 3/19 | 0.002832368 | 0.001727563 | IL12A/IL12B/NFKB1 |
| Human Diseases | Infectious disease: viral | hsa05169 | Epstein-Barr virus infection | 4/19 | 0.003461946 | 0.002111566 | ICAM1/NFKB1/TNFAIP3/RIGI |
| Organismal Systems | Immune system | hsa04640 | Hematopoietic cell lineage | 3/19 | 0.005074594 | 0.003095178 | CD8A/IL7R/IL2RA |
| Human Diseases | Infectious disease: parasitic | hsa05142 | Chagas disease | 3/19 | 0.005074594 | 0.003095178 | IL12A/IL12B/NFKB1 |
| Human Diseases | Infectious disease: parasitic | hsa05146 | Amoebiasis | 3/19 | 0.005074594 | 0.003095178 | IL12A/IL12B/NFKB1 |
| Human Diseases | Infectious disease: viral | hsa05171 | Coronavirus disease - COVID-19 | 4/19 | 0.005137599 | 0.003133607 | IL12A/IL12B/NFKB1/RIGI |
| Organismal Systems | Immune system | hsa04659 | Th17 cell differentiation | 3/19 | 0.005499642 | 0.00335443 | NFKB1/IL2RA/IL23A |
| Human Diseases | Infectious disease: parasitic | hsa05145 | Toxoplasmosis | 3/19 | 0.005719207 | 0.003488351 | IL12A/IL12B/NFKB1 |
| Environmental Information Processing | Signal transduction | hsa04668 | TNF signaling pathway | 3/19 | 0.006557554 | 0.003999688 | ICAM1/NFKB1/TNFAIP3 |
| Human Diseases | Immune disease | hsa05340 | Primary immunodeficiency | 2/19 | 0.009963849 | 0.006077311 | CD8A/IL7R |
| Human Diseases | Immune disease | hsa05330 | Allograft rejection | 2/19 | 0.010128082 | 0.006177482 | IL12A/IL12B |
| Human Diseases | Endocrine and metabolic disease | hsa04940 | Type I diabetes mellitus | 2/19 | 0.012424052 | 0.007577877 | IL12A/IL12B |
| Environmental Information Processing | Signal transduction | hsa04151 | PI3K-Akt signaling pathway | 4/19 | 0.018299086 | 0.011161273 | IL7R/NFKB1/COMP/IL2RA |
| Human Diseases | Infectious disease: viral | hsa05166 | Human T-cell leukemia virus 1 infection | 3/19 | 0.032658746 | 0.019919747 | ICAM1/NFKB1/IL2RA |
| Organismal Systems | Immune system | hsa04623 | Cytosolic DNA-sensing pathway | 2/19 | 0.038769398 | 0.023646854 | NFKB1/RIGI |
| Organismal Systems | Immune system | hsa04657 | IL-17 signaling pathway | 2/19 | 0.047276419 | 0.028835592 | NFKB1/TNFAIP3 |
| Human Diseases | Immune disease | hsa05323 | Rheumatoid arthritis | 2/19 | 0.047276419 | 0.028835592 | ICAM1/IL23A |

**Table S9. The results of PPI analysis.**

| **novel target** | **drug target** | **coexpression** | **experimentally determined interaction** | **database annotated** | **automated textmining** | **combined score** | **drug** | **max phase** |
| --- | --- | --- | --- | --- | --- | --- | --- | --- |
| ASF1A | SIRT1 | 0.109 | 0.198 | 0 | 0.639 | 0.719 | srt-2104 | 2 |
| CD8A | SIRT1 | 0.043 | 0 | 0 | 0.4 | 0.401 | srt-2104 | 2 |
| CD8A | IL23A | 0.056 | 0 | 0 | 0.507 | 0.514 | risankizumab | 4 |
| CD8A | IL23A | 0.056 | 0 | 0 | 0.507 | 0.514 | briakinumab | 3 |
| CD8A | IL23A | 0.056 | 0 | 0 | 0.507 | 0.514 | tildrakizumab | 4 |
| CD8A | IL23A | 0.056 | 0 | 0 | 0.507 | 0.514 | ustekinumab | 4 |
| CD8A | IL23A | 0.056 | 0 | 0 | 0.507 | 0.514 | guselkumab | 4 |
| CD8A | CD80 | 0.055 | 0 | 0 | 0.959 | 0.959 | abatacept | 2 |
| CD8A | CD86 | 0.164 | 0 | 0 | 0.923 | 0.933 | abatacept | 2 |
| CD8A | IL17F | 0 | 0 | 0 | 0.661 | 0.661 | bimekizumab | 4 |
| CD8A | ITGAL | 0.285 | 0 | 0 | 0.798 | 0.849 | efalizumab | 4 |
| CD8A | JAK2 | 0.097 | 0.045 | 0 | 0.596 | 0.621 | tofacitinib | 3 |
| CD8A | JAK2 | 0.097 | 0.045 | 0 | 0.596 | 0.621 | peficitinib | 2 |
| CD8A | TYK2 | 0.067 | 0.045 | 0 | 0.506 | 0.521 | tofacitinib | 3 |
| CD8A | TYK2 | 0.067 | 0.045 | 0 | 0.506 | 0.521 | peficitinib | 2 |
| CD8A | JAK3 | 0.133 | 0.045 | 0 | 0.581 | 0.623 | tofacitinib | 3 |
| CD8A | JAK3 | 0.133 | 0.045 | 0 | 0.581 | 0.623 | peficitinib | 2 |
| CD8A | JAK1 | 0.087 | 0.045 | 0 | 0.622 | 0.641 | tofacitinib | 3 |
| CD8A | JAK1 | 0.087 | 0.045 | 0 | 0.622 | 0.641 | peficitinib | 2 |
| CD8A | IL17A | 0 | 0 | 0 | 0.936 | 0.936 | ixekizumab | 4 |
| CD8A | IL17A | 0 | 0 | 0 | 0.936 | 0.936 | secukinumab | 4 |
| CD8A | IL17A | 0 | 0 | 0 | 0.936 | 0.936 | bimekizumab | 4 |
| CD8A | IL17A | 0 | 0 | 0 | 0.936 | 0.936 | brodalumab | 4 |
| CD8A | TNF | 0.073 | 0 | 0 | 0.957 | 0.958 | certolizumab pegol | 3 |
| CD8A | TNF | 0.073 | 0 | 0 | 0.957 | 0.958 | etanercept | 4 |
| COMP | TNF | 0.06 | 0.071 | 0 | 0.419 | 0.448 | certolizumab pegol | 3 |
| COMP | TNF | 0.06 | 0.071 | 0 | 0.419 | 0.448 | etanercept | 4 |
| CRTAM | IL17A | 0.042 | 0 | 0 | 0.424 | 0.424 | ixekizumab | 4 |
| CRTAM | IL17A | 0.042 | 0 | 0 | 0.424 | 0.424 | secukinumab | 4 |
| CRTAM | IL17A | 0.042 | 0 | 0 | 0.424 | 0.424 | bimekizumab | 4 |
| CRTAM | IL17A | 0.042 | 0 | 0 | 0.424 | 0.424 | brodalumab | 4 |
| CTF1 | TYK2 | 0.047 | 0 | 0.5 | 0.359 | 0.667 | tofacitinib | 3 |
| CTF1 | TYK2 | 0.047 | 0 | 0.5 | 0.359 | 0.667 | peficitinib | 2 |
| CTF1 | JAK1 | 0 | 0 | 0.5 | 0.403 | 0.688 | tofacitinib | 3 |
| CTF1 | JAK1 | 0 | 0 | 0.5 | 0.403 | 0.688 | peficitinib | 2 |
| CTF1 | JAK2 | 0 | 0 | 0.5 | 0.425 | 0.7 | tofacitinib | 3 |
| CTF1 | JAK2 | 0 | 0 | 0.5 | 0.425 | 0.7 | peficitinib | 2 |
| DAPK2 | TNF | 0.07 | 0 | 0 | 0.484 | 0.5 | certolizumab pegol | 3 |
| DAPK2 | TNF | 0.07 | 0 | 0 | 0.484 | 0.5 | etanercept | 4 |
| DDX58 | CD80 | 0.11 | 0.052 | 0 | 0.486 | 0.529 | abatacept | 2 |
| DDX58 | CD86 | 0.123 | 0.047 | 0 | 0.479 | 0.526 | abatacept | 2 |
| DDX58 | IL17A | 0.054 | 0 | 0 | 0.507 | 0.513 | ixekizumab | 4 |
| DDX58 | IL17A | 0.054 | 0 | 0 | 0.507 | 0.513 | secukinumab | 4 |
| DDX58 | IL17A | 0.054 | 0 | 0 | 0.507 | 0.513 | bimekizumab | 4 |
| DDX58 | IL17A | 0.054 | 0 | 0 | 0.507 | 0.513 | brodalumab | 4 |
| DDX58 | JAK2 | 0.225 | 0 | 0 | 0.414 | 0.527 | tofacitinib | 3 |
| DDX58 | JAK2 | 0.225 | 0 | 0 | 0.414 | 0.527 | peficitinib | 2 |
| DDX58 | TYK2 | 0.091 | 0 | 0 | 0.583 | 0.604 | tofacitinib | 3 |
| DDX58 | TYK2 | 0.091 | 0 | 0 | 0.583 | 0.604 | peficitinib | 2 |
| DDX58 | PPIA | 0 | 0.319 | 0 | 0.552 | 0.682 | cyclosporine | 4 |
| DDX58 | TNF | 0.131 | 0 | 0 | 0.712 | 0.739 | certolizumab pegol | 3 |
| DDX58 | TNF | 0.131 | 0 | 0 | 0.712 | 0.739 | etanercept | 4 |
| DDX58 | JAK1 | 0.096 | 0 | 0.4 | 0.582 | 0.753 | tofacitinib | 3 |
| DDX58 | JAK1 | 0.096 | 0 | 0.4 | 0.582 | 0.753 | peficitinib | 2 |
| ICAM1 | SIRT1 | 0 | 0 | 0 | 0.494 | 0.494 | srt-2104 | 2 |
| ICAM1 | NR3C1 | 0.073 | 0.292 | 0 | 0.318 | 0.514 | hydrocortisone butyrate | 1 |
| ICAM1 | NR3C1 | 0.073 | 0.292 | 0 | 0.318 | 0.514 | betamethasone valerate | 1 |
| ICAM1 | NR3C1 | 0.073 | 0.292 | 0 | 0.318 | 0.514 | triamcinolone | 2 |
| ICAM1 | NR3C1 | 0.073 | 0.292 | 0 | 0.318 | 0.514 | betamethasone dipropionate | 4 |
| ICAM1 | NR3C1 | 0.073 | 0.292 | 0 | 0.318 | 0.514 | fluocinolone acetonide | 4 |
| ICAM1 | NR3C1 | 0.073 | 0.292 | 0 | 0.318 | 0.514 | clobetasol propionate | 4 |
| ICAM1 | NR3C1 | 0.073 | 0.292 | 0 | 0.318 | 0.514 | desoximetasone | 4 |
| ICAM1 | NR3C1 | 0.073 | 0.292 | 0 | 0.318 | 0.514 | halobetasol propionate | 4 |
| ICAM1 | RARA | 0.083 | 0 | 0 | 0.508 | 0.53 | alitretinoin | 2 |
| ICAM1 | RARA | 0.083 | 0 | 0 | 0.508 | 0.53 | etretinate | 4 |
| ICAM1 | RARA | 0.083 | 0 | 0 | 0.508 | 0.53 | tazarotene | 4 |
| ICAM1 | RARA | 0.083 | 0 | 0 | 0.508 | 0.53 | acitretin | 4 |
| ICAM1 | CD80 | 0.124 | 0 | 0.4 | 0.997 | 0.998 | abatacept | 2 |
| ICAM1 | JAK2 | 0.09 | 0 | 0 | 0.487 | 0.513 | tofacitinib | 3 |
| ICAM1 | JAK2 | 0.09 | 0 | 0 | 0.487 | 0.513 | peficitinib | 2 |
| ICAM1 | JAK1 | 0.055 | 0 | 0 | 0.597 | 0.603 | tofacitinib | 3 |
| ICAM1 | JAK1 | 0.055 | 0 | 0 | 0.597 | 0.603 | peficitinib | 2 |
| ICAM1 | IL17A | 0.044 | 0 | 0 | 0.697 | 0.697 | ixekizumab | 4 |
| ICAM1 | IL17A | 0.044 | 0 | 0 | 0.697 | 0.697 | secukinumab | 4 |
| ICAM1 | IL17A | 0.044 | 0 | 0 | 0.697 | 0.697 | bimekizumab | 4 |
| ICAM1 | IL17A | 0.044 | 0 | 0 | 0.697 | 0.697 | brodalumab | 4 |
| ICAM1 | TNF | 0.255 | 0 | 0 | 0.929 | 0.944 | certolizumab pegol | 3 |
| ICAM1 | TNF | 0.255 | 0 | 0 | 0.929 | 0.944 | etanercept | 4 |
| ICAM1 | CD86 | 0.149 | 0 | 0.4 | 0.974 | 0.985 | abatacept | 2 |
| ICAM1 | ITGAL | 0.124 | 0.983 | 0.9 | 0.997 | 0.999 | efalizumab | 4 |
| ICAM5 | ITGAL | 0 | 0.946 | 0.5 | 0.875 | 0.996 | efalizumab | 4 |
| IL12A | IL23A | 0.061 | 0 | 0 | 0.395 | 0.408 | risankizumab | 4 |
| IL12A | IL23A | 0.061 | 0 | 0 | 0.395 | 0.408 | briakinumab | 3 |
| IL12A | IL23A | 0.061 | 0 | 0 | 0.395 | 0.408 | tildrakizumab | 4 |
| IL12A | IL23A | 0.061 | 0 | 0 | 0.395 | 0.408 | ustekinumab | 4 |
| IL12A | IL23A | 0.061 | 0 | 0 | 0.395 | 0.408 | guselkumab | 4 |
| IL12A | IL12B | 0.062 | 0.977 | 0.9 | 0.878 | 0.999 | risankizumab | 4 |
| IL12A | IL12B | 0.062 | 0.977 | 0.9 | 0.878 | 0.999 | briakinumab | 3 |
| IL12A | IL12B | 0.062 | 0.977 | 0.9 | 0.878 | 0.999 | tildrakizumab | 4 |
| IL12A | IL12B | 0.062 | 0.977 | 0.9 | 0.878 | 0.999 | ustekinumab | 4 |
| IL12A | JAK1 | 0 | 0 | 0.5 | 0.129 | 0.546 | tofacitinib | 3 |
| IL12A | JAK1 | 0 | 0 | 0.5 | 0.129 | 0.546 | peficitinib | 2 |
| IL12A | TYK2 | 0 | 0 | 0.54 | 0.11 | 0.573 | tofacitinib | 3 |
| IL12A | TYK2 | 0 | 0 | 0.54 | 0.11 | 0.573 | peficitinib | 2 |
| IL12A | JAK2 | 0.049 | 0 | 0.54 | 0.108 | 0.576 | tofacitinib | 3 |
| IL12A | JAK2 | 0.049 | 0 | 0.54 | 0.108 | 0.576 | peficitinib | 2 |
| IL12A | TNF | 0.083 | 0 | 0.4 | 0.296 | 0.579 | certolizumab pegol | 3 |
| IL12A | TNF | 0.083 | 0 | 0.4 | 0.296 | 0.579 | etanercept | 4 |
| IL2RA | IL23A | 0.072 | 0 | 0.65 | 0.181 | 0.71 | risankizumab | 4 |
| IL2RA | IL23A | 0.072 | 0 | 0.65 | 0.181 | 0.71 | briakinumab | 3 |
| IL2RA | IL23A | 0.072 | 0 | 0.65 | 0.181 | 0.71 | tildrakizumab | 4 |
| IL2RA | IL23A | 0.072 | 0 | 0.65 | 0.181 | 0.71 | ustekinumab | 4 |
| IL2RA | IL23A | 0.072 | 0 | 0.65 | 0.181 | 0.71 | guselkumab | 4 |
| IL2RA | IL12B | 0.154 | 0 | 0.65 | 0.3 | 0.828 | risankizumab | 4 |
| IL2RA | IL12B | 0.154 | 0 | 0.65 | 0.3 | 0.828 | briakinumab | 3 |
| IL2RA | IL12B | 0.154 | 0 | 0.65 | 0.3 | 0.828 | tildrakizumab | 4 |
| IL2RA | IL12B | 0.154 | 0 | 0.65 | 0.3 | 0.828 | ustekinumab | 4 |
| IL2RA | CD80 | 0.168 | 0 | 0 | 0.591 | 0.646 | abatacept | 2 |
| IL2RA | CD86 | 0.144 | 0 | 0 | 0.551 | 0.599 | abatacept | 2 |
| IL2RA | IL17F | 0.123 | 0 | 0 | 0.422 | 0.471 | bimekizumab | 4 |
| IL2RA | ITGAL | 0.085 | 0 | 0 | 0.389 | 0.417 | efalizumab | 4 |
| IL2RA | TYK2 | 0.06 | 0 | 0.65 | 0.42 | 0.792 | tofacitinib | 3 |
| IL2RA | TYK2 | 0.06 | 0 | 0.65 | 0.42 | 0.792 | peficitinib | 2 |
| IL2RA | IL17A | 0.085 | 0 | 0 | 0.796 | 0.805 | ixekizumab | 4 |
| IL2RA | IL17A | 0.085 | 0 | 0 | 0.796 | 0.805 | secukinumab | 4 |
| IL2RA | IL17A | 0.085 | 0 | 0 | 0.796 | 0.805 | bimekizumab | 4 |
| IL2RA | IL17A | 0.085 | 0 | 0 | 0.796 | 0.805 | brodalumab | 4 |
| IL2RA | TNF | 0.12 | 0 | 0 | 0.865 | 0.876 | certolizumab pegol | 3 |
| IL2RA | TNF | 0.12 | 0 | 0 | 0.865 | 0.876 | etanercept | 4 |
| IL2RA | JAK2 | 0.084 | 0 | 0.8 | 0.396 | 0.879 | tofacitinib | 3 |
| IL2RA | JAK2 | 0.084 | 0 | 0.8 | 0.396 | 0.879 | peficitinib | 2 |
| IL2RA | JAK1 | 0.068 | 0 | 0.9 | 0.79 | 0.978 | tofacitinib | 3 |
| IL2RA | JAK1 | 0.068 | 0 | 0.9 | 0.79 | 0.978 | peficitinib | 2 |
| IL2RA | JAK3 | 0.159 | 0 | 0.9 | 0.957 | 0.996 | tofacitinib | 3 |
| IL2RA | JAK3 | 0.159 | 0 | 0.9 | 0.957 | 0.996 | peficitinib | 2 |
| IL7R | IL23A | 0.103 | 0 | 0.65 | 0.263 | 0.748 | risankizumab | 4 |
| IL7R | IL23A | 0.103 | 0 | 0.65 | 0.263 | 0.748 | briakinumab | 3 |
| IL7R | IL23A | 0.103 | 0 | 0.65 | 0.263 | 0.748 | tildrakizumab | 4 |
| IL7R | IL23A | 0.103 | 0 | 0.65 | 0.263 | 0.748 | ustekinumab | 4 |
| IL7R | IL23A | 0.103 | 0 | 0.65 | 0.263 | 0.748 | guselkumab | 4 |
| IL7R | IL12B | 0.048 | 0 | 0.65 | 0.082 | 0.667 | risankizumab | 4 |
| IL7R | IL12B | 0.048 | 0 | 0.65 | 0.082 | 0.667 | briakinumab | 3 |
| IL7R | IL12B | 0.048 | 0 | 0.65 | 0.082 | 0.667 | tildrakizumab | 4 |
| IL7R | IL12B | 0.048 | 0 | 0.65 | 0.082 | 0.667 | ustekinumab | 4 |
| IL7R | CD80 | 0.162 | 0 | 0 | 0.595 | 0.647 | abatacept | 2 |
| IL7R | IL17F | 0 | 0 | 0 | 0.453 | 0.453 | bimekizumab | 4 |
| IL7R | ITGAL | 0.204 | 0 | 0 | 0.525 | 0.606 | efalizumab | 4 |
| IL7R | CD86 | 0.166 | 0 | 0 | 0.599 | 0.651 | abatacept | 2 |
| IL7R | TNF | 0.115 | 0 | 0 | 0.694 | 0.717 | certolizumab pegol | 3 |
| IL7R | TNF | 0.115 | 0 | 0 | 0.694 | 0.717 | etanercept | 4 |
| IL7R | IL17A | 0 | 0 | 0 | 0.813 | 0.813 | ixekizumab | 4 |
| IL7R | IL17A | 0 | 0 | 0 | 0.813 | 0.813 | secukinumab | 4 |
| IL7R | IL17A | 0 | 0 | 0 | 0.813 | 0.813 | bimekizumab | 4 |
| IL7R | IL17A | 0 | 0 | 0 | 0.813 | 0.813 | brodalumab | 4 |
| IL7R | TYK2 | 0.059 | 0.091 | 0.65 | 0.53 | 0.84 | tofacitinib | 3 |
| IL7R | TYK2 | 0.059 | 0.091 | 0.65 | 0.53 | 0.84 | peficitinib | 2 |
| IL7R | JAK2 | 0.098 | 0.046 | 0.8 | 0.489 | 0.9 | tofacitinib | 3 |
| IL7R | JAK2 | 0.098 | 0.046 | 0.8 | 0.489 | 0.9 | peficitinib | 2 |
| IL7R | JAK3 | 0.128 | 0.295 | 0.8 | 0.919 | 0.988 | tofacitinib | 3 |
| IL7R | JAK3 | 0.128 | 0.295 | 0.8 | 0.919 | 0.988 | peficitinib | 2 |
| IL7R | JAK1 | 0.121 | 0.095 | 0.9 | 0.942 | 0.994 | tofacitinib | 3 |
| IL7R | JAK1 | 0.121 | 0.095 | 0.9 | 0.942 | 0.994 | peficitinib | 2 |
| LBP | IL23A | 0 | 0 | 0.4 | 0 | 0.4 | risankizumab | 4 |
| LBP | IL23A | 0 | 0 | 0.4 | 0 | 0.4 | briakinumab | 3 |
| LBP | IL23A | 0 | 0 | 0.4 | 0 | 0.4 | tildrakizumab | 4 |
| LBP | IL23A | 0 | 0 | 0.4 | 0 | 0.4 | ustekinumab | 4 |
| LBP | IL23A | 0 | 0 | 0.4 | 0 | 0.4 | guselkumab | 4 |
| LBP | IL17F | 0.042 | 0 | 0.4 | 0.097 | 0.435 | bimekizumab | 4 |
| LBP | IL17A | 0 | 0 | 0.4 | 0.275 | 0.546 | ixekizumab | 4 |
| LBP | IL17A | 0 | 0 | 0.4 | 0.275 | 0.546 | secukinumab | 4 |
| LBP | IL17A | 0 | 0 | 0.4 | 0.275 | 0.546 | bimekizumab | 4 |
| LBP | IL17A | 0 | 0 | 0.4 | 0.275 | 0.546 | brodalumab | 4 |
| LBP | TNF | 0 | 0 | 0 | 0.689 | 0.689 | certolizumab pegol | 3 |
| LBP | TNF | 0 | 0 | 0 | 0.689 | 0.689 | etanercept | 4 |
| MMP12 | TNF | 0.087 | 0 | 0 | 0.711 | 0.725 | certolizumab pegol | 3 |
| MMP12 | TNF | 0.087 | 0 | 0 | 0.711 | 0.725 | etanercept | 4 |
| MMP12 | IL17A | 0.094 | 0 | 0 | 0.589 | 0.611 | ixekizumab | 4 |
| MMP12 | IL17A | 0.094 | 0 | 0 | 0.589 | 0.611 | secukinumab | 4 |
| MMP12 | IL17A | 0.094 | 0 | 0 | 0.589 | 0.611 | bimekizumab | 4 |
| MMP12 | IL17A | 0.094 | 0 | 0 | 0.589 | 0.611 | brodalumab | 4 |
| NFKB1 | SIRT1 | 0.049 | 0.33 | 0.9 | 0.768 | 0.983 | srt-2104 | 2 |
| NFKB1 | IL17F | 0.098 | 0 | 0 | 0.371 | 0.409 | bimekizumab | 4 |
| NFKB1 | NTRK1 | 0 | 0.095 | 0 | 0.391 | 0.425 | pegcantratinib | 2 |
| NFKB1 | JAK3 | 0.109 | 0.095 | 0 | 0.409 | 0.482 | tofacitinib | 3 |
| NFKB1 | JAK3 | 0.109 | 0.095 | 0 | 0.409 | 0.482 | peficitinib | 2 |
| NFKB1 | TYK2 | 0.084 | 0.095 | 0 | 0.446 | 0.501 | tofacitinib | 3 |
| NFKB1 | TYK2 | 0.084 | 0.095 | 0 | 0.446 | 0.501 | peficitinib | 2 |
| NFKB1 | RXRA | 0.065 | 0.292 | 0 | 0.371 | 0.548 | alitretinoin | 2 |
| NFKB1 | RXRA | 0.065 | 0.292 | 0 | 0.371 | 0.548 | etretinate | 4 |
| NFKB1 | RXRA | 0.065 | 0.292 | 0 | 0.371 | 0.548 | tazarotene | 4 |
| NFKB1 | RXRA | 0.065 | 0.292 | 0 | 0.371 | 0.548 | acitretin | 4 |
| NFKB1 | JAK2 | 0.128 | 0.095 | 0 | 0.601 | 0.657 | tofacitinib | 3 |
| NFKB1 | JAK2 | 0.128 | 0.095 | 0 | 0.601 | 0.657 | peficitinib | 2 |
| NFKB1 | JAK1 | 0.154 | 0.235 | 0 | 0.537 | 0.675 | tofacitinib | 3 |
| NFKB1 | JAK1 | 0.154 | 0.235 | 0 | 0.537 | 0.675 | peficitinib | 2 |
| NFKB1 | CD80 | 0.13 | 0 | 0 | 0.675 | 0.705 | abatacept | 2 |
| NFKB1 | CD86 | 0.13 | 0 | 0 | 0.688 | 0.717 | abatacept | 2 |
| NFKB1 | IL17A | 0.051 | 0 | 0 | 0.789 | 0.791 | ixekizumab | 4 |
| NFKB1 | IL17A | 0.051 | 0 | 0 | 0.789 | 0.791 | secukinumab | 4 |
| NFKB1 | IL17A | 0.051 | 0 | 0 | 0.789 | 0.791 | bimekizumab | 4 |
| NFKB1 | IL17A | 0.051 | 0 | 0 | 0.789 | 0.791 | brodalumab | 4 |
| NFKB1 | NR3C1 | 0.126 | 0.51 | 0 | 0.74 | 0.878 | hydrocortisone butyrate | 1 |
| NFKB1 | NR3C1 | 0.126 | 0.51 | 0 | 0.74 | 0.878 | betamethasone valerate | 1 |
| NFKB1 | NR3C1 | 0.126 | 0.51 | 0 | 0.74 | 0.878 | triamcinolone | 2 |
| NFKB1 | NR3C1 | 0.126 | 0.51 | 0 | 0.74 | 0.878 | betamethasone dipropionate | 4 |
| NFKB1 | NR3C1 | 0.126 | 0.51 | 0 | 0.74 | 0.878 | fluocinolone acetonide | 4 |
| NFKB1 | NR3C1 | 0.126 | 0.51 | 0 | 0.74 | 0.878 | clobetasol propionate | 4 |
| NFKB1 | NR3C1 | 0.126 | 0.51 | 0 | 0.74 | 0.878 | desoximetasone | 4 |
| NFKB1 | NR3C1 | 0.126 | 0.51 | 0 | 0.74 | 0.878 | halobetasol propionate | 4 |
| NFKB1 | TNF | 0.194 | 0 | 0.9 | 0.995 | 0.999 | certolizumab pegol | 3 |
| NFKB1 | TNF | 0.194 | 0 | 0.9 | 0.995 | 0.999 | etanercept | 4 |
| RARRES2 | TNF | 0 | 0 | 0 | 0.522 | 0.522 | certolizumab pegol | 3 |
| RARRES2 | TNF | 0 | 0 | 0 | 0.522 | 0.522 | etanercept | 4 |
| TIMD4 | CD80 | 0.146 | 0.126 | 0 | 0.355 | 0.476 | abatacept | 2 |
| TIMD4 | CD86 | 0.15 | 0 | 0 | 0.369 | 0.441 | abatacept | 2 |
| TIMD4 | TNF | 0.078 | 0 | 0 | 0.556 | 0.573 | certolizumab pegol | 3 |
| TIMD4 | TNF | 0.078 | 0 | 0 | 0.556 | 0.573 | etanercept | 4 |
| TNFAIP3 | JAK2 | 0.14 | 0.066 | 0 | 0.331 | 0.416 | tofacitinib | 3 |
| TNFAIP3 | JAK2 | 0.14 | 0.066 | 0 | 0.331 | 0.416 | peficitinib | 2 |
| TNFAIP3 | TNF | 0.479 | 0.994 | 0.5 | 0.664 | 0.999 | certolizumab pegol | 3 |
| TNFAIP3 | TNF | 0.479 | 0.994 | 0.5 | 0.664 | 0.999 | etanercept | 4 |
| TNFAIP3 | TYK2 | 0.117 | 0.066 | 0 | 0.434 | 0.492 | tofacitinib | 3 |
| TNFAIP3 | TYK2 | 0.117 | 0.066 | 0 | 0.434 | 0.492 | peficitinib | 2 |
| TNFAIP3 | IL17A | 0.065 | 0 | 0 | 0.471 | 0.484 | ixekizumab | 4 |
| TNFAIP3 | IL17A | 0.065 | 0 | 0 | 0.471 | 0.484 | secukinumab | 4 |
| TNFAIP3 | IL17A | 0.065 | 0 | 0 | 0.471 | 0.484 | bimekizumab | 4 |
| TNFAIP3 | IL17A | 0.065 | 0 | 0 | 0.471 | 0.484 | brodalumab | 4 |
